# Exosomal microRNA-107 reverses chemotherapeutic drug resistance of gastric cancer cells through HMGA2/mTOR/P-gp pathway

**DOI:** 10.1186/s12885-021-09020-y

**Published:** 2021-12-02

**Authors:** Lu Jiang, Yan Zhang, Linghui Guo, Chaoyang Liu, Pan Wang, Weihong Ren

**Affiliations:** 1 Henan University of Chinese Medicine, 156 Jinshui East Road, Zhengzhou, 450046 China; 2 School of Life and Health Sciences, The Chinese University of Hong Kong (Shenzhen), 2001 Longxiang Ave, Shenzhen, 518172 China; 3 Department of Clinical Laboratory, The First Affiliated Hospital of Henan University of Chinese Medicine, 19 Renmin Road, Zhengzhou, 450000 China

**Keywords:** Gastric cancer, Reverse drug resistance, Exosomal miR-107, HMGA2/mTOR/P-gp

## Abstract

**Background:**

RNA cargo in exosomes, especially microRNAs (miRNAs), play an important role in the chemotherapy drug resistance of human cancers. However, the role and mechanism of exosomal miR-107 on multidrug resistance of gastric cancer cells was still not clear. In this study, we sought to explore whether exosomal miR-107 could reverse the resistance of gastric cancer cells to the chemotherapy drugs.

**Methods:**

We extracted exosomes from sensitive (SGC-7901, MGC-803) and resistant (SGC-7901/5-FU) gastric cancer cells by ultracentrifugation and the isolated exosomes were identified using transmission electron microscopy (TEM) and dynamic light scattering analysis (DLS). The expression of miR-107 and high mobility group A2 (HMGA2) were detected by real-time quantitative PCR (RT-qPCR). MTT assay was used to investigate the effect of exosomes on gastric cancer cells growth in vitro. The uptake of exosomes by recipient cells were observed using a fluorescence microscope. The predicted target relationship between miR-107 and HMGA2 was verified by *gauss*-luciferase reporter assay. The expression of HMGA2, p-mTOR/mTOR, P-gp and other exosomal indicated marker proteins was detected by western blot.

**Results:**

Our results indicated that the isolated exosomes were typically cup-like lipid bilayer membranes structure. SGC-7901/5-FU cells were cross-resistant to chemotherapy drug cisplatin (CDDP), and the sensitive cells-secreted exosomes drastically reversed the resistance of the resistant GC cells to the chemotherapeutic drugs, which was verified by exosomal inhibitor GW4896. Mechanistically, the reversal effect was mainly mediated by exosome-secreted miR-107 through downregulating the expression of target molecular HMGA2 and inhibiting HMGA2/mTOR/P-gp pathway, which were supported by results from luciferase reporter assay and rescue assay.

**Conclusions:**

These findings demonstrated that exosome-transmitted miR-107 significantly enhanced the sensitivity of resistant gastric cancer cells to chemotherapeutic agents by mediating the HMGA2/mTOR/P-gp axis and exosomal miR-107 may be a novel target in gastric cancers treatment.

**Supplementary Information:**

The online version contains supplementary material available at 10.1186/s12885-021-09020-y.

## Background

Gastric cancer (GC) is the fifth most common malignant tumor in the world and the third leading cause of cancer-related deaths, posing a serious threat to human life and health [1]. Due to the lack of effective biomolecular markers, GC is usually diagnosed at an advanced stage, and its 5-year survival rate is about 20-30% [2, 3]. Various factors including genetics, epigenetics and environment could affect the occurrence and progression of GC [4]. At the present stage, there is still lacking of effective treatment methods in the clinical treatment of advanced gastric cancer, although targeted therapy or a combination of targeted therapy and chemotherapy could improve the effect to a certain extent, surgery and chemotherapy are still the major approaches for treatment. The chemotherapy drugs 5-Fluorouracil, cisplatin, doxorubicin and paclitaxel are commonly used for GC treatment, which inevitably led to chemotherapy drug resistance [5]. Therefore, exploring the mechanism of drug resistance in GC is urgently needed clinically, and would make it possible for development of clinical treatment of resistant GC.

Exosomes are a class of extracellular vesicles with 30-150 nm in diameter, and have a lipid bilayer structure, which are similar to plasma membranes. The membrane has a variety of proteins (CD9, CD63, CD81 and integrin, etc.) and lipids (ceramide, phosphatidylethanolamine, phosphatidylserine, etc.), and there are also a variety of nucleic acids (mRNA, miRNA, LncRNA, circRNA and DNA) and proteins (TSG101, Alix, Hsc70 and Hsp90, etc.) inside of exosomes [6]. Exosomes could transport a variety of biologically active molecules (such as nucleic acids, lipids, and proteins) to the recipient cells by binding to the recipient cells. Exosomes mediate the information exchange between cells, and regulate a variety of cellular physiological and pathological processes [7]. Expecially, due to the lipid membranes, the exosomal miRNAs are protected from being damaged by cellular environment. Many studies have reported cancer cells-derived exosomal miRNAs played important roles in mediating cellular immune response and tumor angiogenesis, drug resistance and metastasis [8, 9]. Some literatures demonstrated that a variety of exosomal miRNAs could regulate chemotherapy resistance of cancer cells [10, 11].

MicroRNA-107 was a new non-coding RNA discovered in recent years, several studies have illustrated that miRNA-107 played an important role in various diseases process, such as cancers, Alzheimer’s disease, and osteoarthritis [12–14]. Although there were studies showing that the sensitivity of resistant non-small cell lung cancer cells and breast cancer cells to chemotherapy drug paclitaxel could be regulated by miRNA-107 [15, 16], also the expression level of miRNA-107 might be an effective biomarker for poor prognosis of GC patients [17], whether exosomal miR-107 also regulates on multidrug resistance of cancer cells has not been elucidated, and the detailed underlying mechanisms of how exosomal miR-107 regulates the sensitivity of cancer cells to the chemotherapeutic drugs remain largely unknown.

In this study, we sought to explore the effects of exosomal miR-107 on chemotherapeutic drug-resistance and found that exosomal miR-107 extracted from sensitive GC cells could increase the sensitivity of resistant GC cells. We also identified that the target molecules of exosomal miR-107 was HMGA2, which was a small non-histone nuclear protein, and a framework transcription factor. HMGA2 could change the DNA conformation, or directly interact with related proteins, and enhance or inhibit the transcription of the genes through binding to the chromatin enriched AT sequences via its AT hook structure [18]. Our results showed that the reversal effect of exosomal miR-107 on resistant GC was mediated by regulating the expression of target molecular HMGA2, the activity of mTOR and the expression of P-gp.

## Materials and methods

### Cell viability analysis

5-FU sensitive/resistant human gastric cancer cell line SGC-7901 and CDDP sensitive/resistant human gastric cancer cell line SGC-7901 (both from Huiying BioTech), MGC-803 human gastric cancer and HEK 293 T cell lines (both from Beina BioTech), were maintained in RPMI-1640 and DMEM-H medium (Sigma-Aldrich), supplemented with 10% FBS at 37 °C in a humidified atmosphere with 5% CO2, respectively. Different concentrations of 5-FU or cisplatin (Meilun BioTech) were used to treat the cells. After 48 h incubation, cells were subjected to MTT analysis and the absorbance at 570 nm was recorded by a Spectra Max i3 microplate reader (Molecular Devices Corp., Sunnyvale, CA, USA).

### Exosome isolation

The SGC-7901 and SGC-7901/5-FU GC cells were cultured in 1640 medium containing 10% exosome-free fetal bovine serum (FBS), and exosomes were extracted by ultracentrifugation method. The collected cell supernatant was centrifuged at 300×g for 10 min, 2000×g for 20 min, 10,000×g for 20 min, respectively. Then it was filtered by a 0.22 μM filter and centrifuged at 3500×g for 5 min in ultrafiltration tubes. Gradient centrifugation was performed for the concentrated supernatant: 80,000×g/40 min, 80,000×g/80 min, 110,000×g/40 min, 110,000×g/80 min, 110,000×g/120 min, 140,000×g/40 min, 140,000×g/80 min, 140,000×g/120 min at 4 °C. Finally, exosomes were washed and resuspended by PBS. The diameter of exosomes was detected by dynamic light scattering (DLS) as described below. The concentration of exosomes was measured by BCA protein assay kit.

### Transmission electron microscopy

The morphology and size of exosomes was observed by a transmission electron microscopy (JEM-1400, Tokyo, Japan). The experiment was performed by a professional technician from the Electron Microscopy Center of Henan University of Traditional Chinese Medicine. Fifteen microliters of the prepared exosomes, positive (pure milk) and negative (ddH_2_O) controls were pipetted onto carbon-coated copper grids, incubated for 150 s and excess fluids were wiped off. The absorbed exosomes were stained with 3% uranyl acetate for 3 min, washed with ddH_2_O, air-dried for 2 h, and analyzed with a transmission electron microscope at 80-120 kV voltage.

### Dynamic light scattering analysis

A NanoBrook Zetasizer 90Plus PALS (Nano ZS) (Malvern, UK) was applied for dynamic light scattering. Isolated exosome samples were diluted in PBS. All samples were measured with parameters of using a helium/neon laser (640 nm) at 220 V voltage, a temperature of 25 °C and an angle of 90°. The exosome size refers to the scattering intensity distribution (z-average) and effective diameter size calculated based on scattering intensity.

### Exosomal uptake

#### PKH26 staining

Exosomes derived from SGC-7901 cells were labelled with PKH26 kit (PKH26 Red Fluorescent Cell Linker Kit, Sigma, USA). 50 μL ultracentrifugation exosomes suspended in PBS were added with 100 μL Solution C. 0.5 μL PKH26 was dissolved in another 100 μL solution C. The diluted exosomes were added to the diluted PKH26 rapidly, mixed and incubated for 5 min, and 250 μL sterile FBS was added to stop staining. The exosomes labelled with PKH26 were isolated using ExoQuick™ Exosome Isolation Reagent (SBI, USA). 200 μg exosomes labelled with PKH26 were added to SGC-7901/ADR cells. After 24 h, the SGC-7901/ADR cells were fixed by 4% paraformaldehyde, stained with DAPI, and washed with PBS for three times. Fluorescence signal of PKH26 was observed under a Carl Zeiss LSM710 laser scanning confocal microscope (Oberkochen, Germany).

### Transwell coculture

A stable MGC-803 cell line with exosomes labeled with green fluorescence (MGC-803/pLVX-CD63-AcGFP1) was constructed by our laboratory. MGC-803 cells line or MGC-803-pLVX stable cells line (4 × 10^5^/well) were seeded in the upper chamber of a coculture system with a 0.4 μm pore membrane, and the recipient SGC-7901/5-FU (2 × 10^5^/well) were placed in the lower chamber. All cells were incubated in medium with 10% exosome-free FBS. After 48 h of coculture, SGC-7901/5-FU cells were observed under a fluorescence microscope, or collected, washed with PBS, resuspended in 500 μl of PBS and the fluorescence signal were detected by FACS Aria II flow cytometry (Becton Dickinson, USA).

### GW4869 treatment

GW4869, an inhibitor for the exosome formation and release [19, 20], was firstly dissolved in DMSO into a stock solution of 1.5 mM, then added with 5% methanesulfonic acid to increase the solubility in DMSO. SGC7901 cells were treated with GW4869 at a final concentration of 10 μM for 24 h.

### miRNA inhibitor, siRNA and plasmid vectors transfection

SGC-7901 cells were transfected with a miR-107 inhibitor (the sequences were 5′-UGAUAGCCCUGUACAAUGCUGCU-3′) or miR-107 inhibitor negative control (the sequences were 5′-CAGUACUUUUGUGUAGUACAA-3′) at 100 nM with Lipofectamine 2000 (invitrogen, CA, USA). Three siRNAs targeting HMGA2 and negative control were synthesized. The siRNA with the highest gene silencing efficacy was chosen for further use. All the miRNA (inhibitor or mimic) and siRNA were synthesized by Shanghai Sangon Biotech (Shanghai, China). Co-transfection of 50 nM miR-107 inhibitor and 50 nM HMGA2 siRNA were included. pEX-HMGA2-WT and HMGA2 deletion mutant pEX-HMGA2-MUT43 (43-109 aa were deleted, including the central and the last AT-hook DNA binding domains) plasmid vectors [21] were synthesized by Shanghai Gene Pharma Co. Ltd. (Shanghai, China).

### Luciferase reporter assay

The targeted binding site of miR-107 to HMGA2 was predicted using TargetScan (http://www.targetscan.org/vert_72/). HMGA2 wild-type (insert sequence: 5′-gtcTAGTACTTATTAC-ATGCTGCt-3′) and mutant-type (insert sequence: 5′-gtcTAGTACTTATTACTACGACGt-3′) expression vectors were cloned into a *Gaussia luciferase* (GLuc) reporter vector (pEZX-MT05, Genecopoeia), which contains a reference gene called secreted alkaline phosphatase (SeAP). The HEK 293 T cells in 24-well plates were co-transfected with the above wild or mutant reporter vectors with miR-107 mimic or NC using transfection reagent Lipofectamine 2000 for 48 h. The activities of GLuc and SeAP were quantified with the secrete-pair dual luminescence assay kit (Genecopoeia).

### Western blotting analysis

Protein extraction of exosomes and cells as well as Western blot analysis were performed according to our previous study [22]. The following antibodies were used: anti-Lamin B1 (#13435, 1:1000), anti-HSP 70 (#4872, 1:1000), anti-p-mTOR (#5536, 1:1000), anti-mTOR (#2983, 1:1000), anti-P-gp (#13342, 1:500) (All from Cell Signaling); anti-CD63 (D360973, 1:500) and anti-HMGA2 (D160487, 1:500) (Both from BBI) and anti-GAPDH (CW0100, 1:1000, Beijing Com Win).

### Quantitative PCR analysis

mRNA extraction of cells as well as the relative mRNA level of HMGA2 gene analyzed by quantitative PCR were performed according to our previous study [22]. The primer sequences for HMGA2 were 5′-TGGGAGGAGCGAAATCTAA-3′ (sense) and 5′-GGTGAACTCAAGCCGAAG-3′ (antisense) and the primer sequences for the control gene GAPDH were 5′-CGCTGAGTACGTCGTGGAGTC-3′ (sense) and 5′-GCTGATGATCTTGAGGCTGTTGTC-3′ (antisense). Total microRNA was extracted from cells and exosomes using miRNA Purification kit (CW0627S, Beijing Com Win), microRNA (100 ~ 300 ng) was reverse transcribed into cDNA by using miRNA cDNA Synthesis kit (CW2141S, Beijing Com Win), and the relative level of miR-107 was determined by qPCR using miRNA qPCR Assay kit (CW2142S, Beijing Com Win). The relative expression of miR-107 was normalized to U6, and the antisense primers of miR-107 and U6 were provided by the above kit. The sense primer sequences for miR-107 were 5′- CGCAGCAGCATTGTACAGGGCTATCA-3′ and the sense primer sequences for U6 were 5′-CCGAGAGAAGATTAGCATGGCCCCTG-3′.

### Statistics

All experiments were repeated at least three times except that some WB experiments were repeated twice. A one-way analysis of variance (ANOVA) followed by Dunnett’s test was used for multiple comparisons.

Values of *P < 0.05* were considered significant, and values of *P < 0.01* were considered extremely significant. All data expressed as mean ± SD unless otherwise indicated.

## Results

Isolation and Characterization of exosomes derived from SGC-7901 and SGC-7901/5-FU GC cells were isolated and confirmed.

**Fig. 1:**
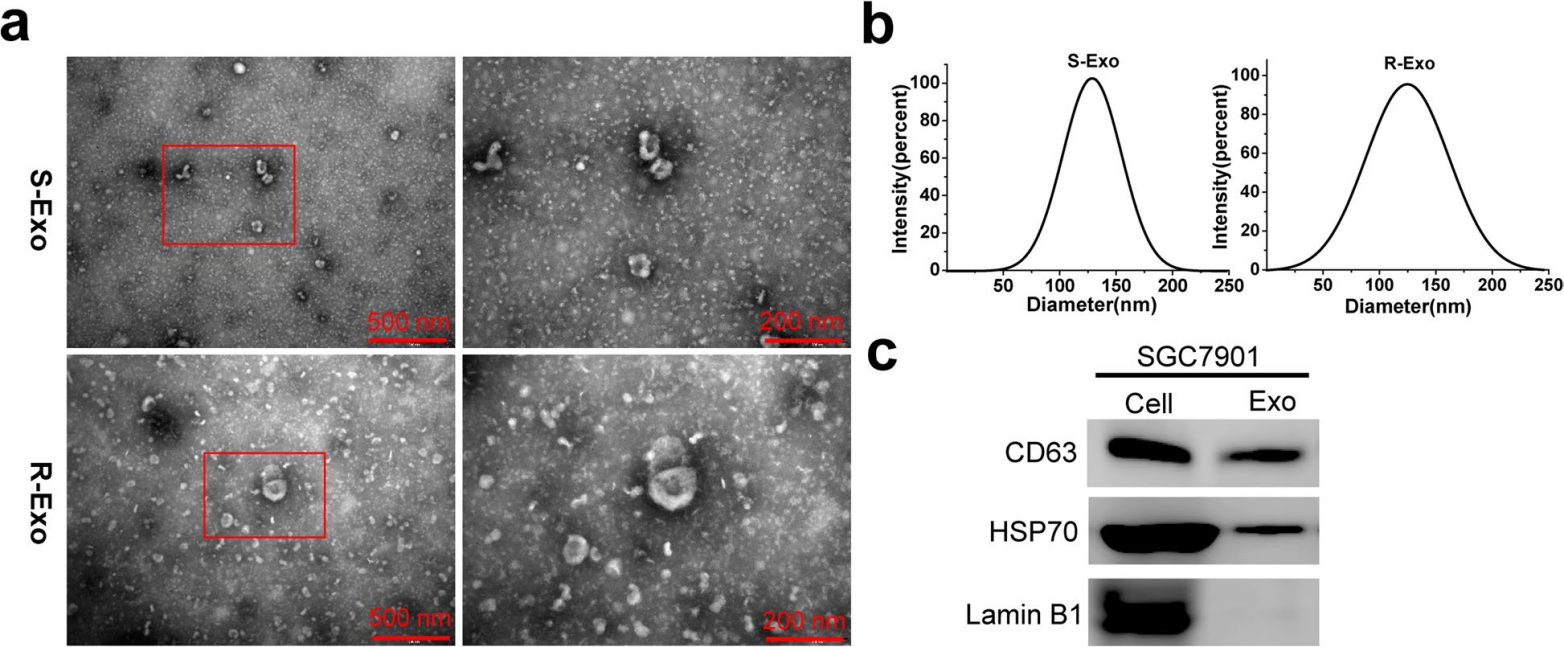
Characteristics of exosomes derived from SGC-7901 and SGC-7901/5-FU GC cells. **a** The transmission electron micrograph showed round-shaped vesicles with bilayer membranes ranging from 30 nm to 180 nm in diameter released by SGC-7901 (S-Exo) and SGC-7901/5-FU (R-Exo) cells. Scale bar = 500 nm and 200 nm, respectively. **b** Dynamic light scattering analysis (DLS) indicated that the dominant size of S- and R-Exo was about 120 nm. **c** The positive markers of exosomes, CD63 and HSP70, were detected in S- and R-Exo by Western blot

In order to study the effects of exosomes on the sensitivity of SGC-7901 GC cells to chemotherapy, we used ultracentrifugation to extract the exosomes of SGC-7901 and SGC-7901/5-FU cells. It can be observed that the vesicles of sensitive and resistant SGC-7901 cells were goblet-shaped with bilayer membranes, and the diameter ranged from 30 nm to 180 nm (Fig. 1a). some proteins were also found in the background under the transmission electron microscope.

The size and diameter of dominant exosomes were further analyzed by DLS. The results showed that the particle sizes were normally distributed around 120 nm (Fig. 1b). Through western blot analysis, the exosomal marker proteins CD63 and HSP70 were detected in the exosomes, while the nuclear marker protein Lamin B1 was mainly enriched in the whole cell lysates (Fig. 1c). These results indicated that the vesicles extracted from sensitive and resistant SGC-7901 cells exhibited typical exosomal characteristics.

### SGC-7901/5-FU cells were resistant to chemotherapy drugs 5-fluorouracil and cisplatin

**Fig. 2:**
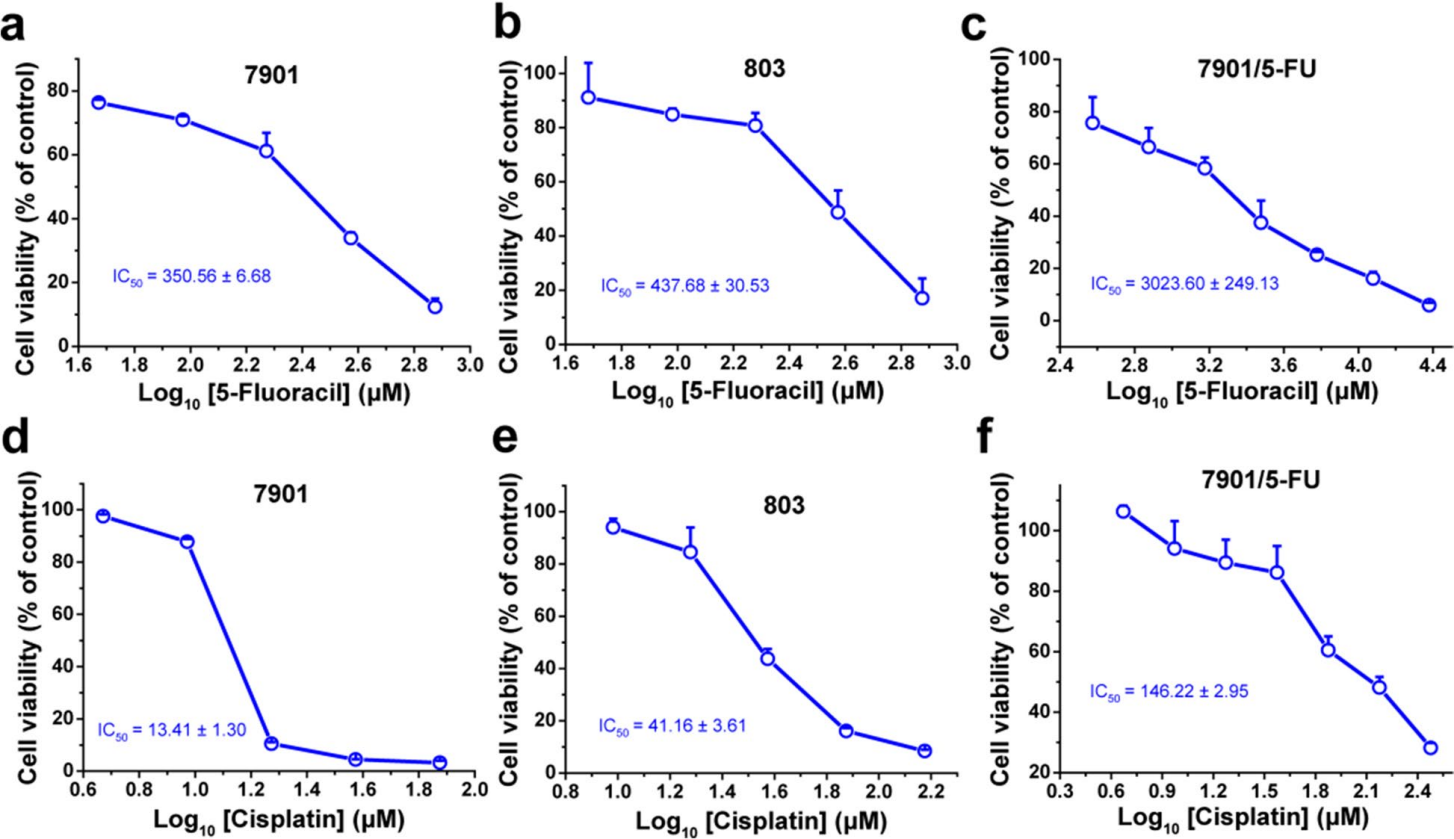
The sensitivity of GC cells to the chemotherapy drugs 5-FU and CDDP. The cell viability of SGC-7901 (**a** and **d**), MGC-803 (**b** and **e**) sensitive cell strains and SGC-7901/5-FU (**c** and **f**) resistant cell strains was determined after treated with 5-FU for 48 h (**a, b, c**) and CDDP (**d, e, f**) for 24 h, respectively. Cell viability was determined by MTT assay. Cells treated with vehicle serve as a blank control. All experiments were conducted in quintuplicates and data were expressed as the mean ± SD (*n* = 5)

We first determined the drug sensitivity of SGC-7901, MGC-803 sensitive cells and SGC-7901/5-FU resistant cells to the chemotherapy drugs 5-fluorouracil (5-FU) and cisplatin (CDDP). As shown in Fig. 2, the SGC-7901/5-FU cell lines were resistant to 5-FU and also cross-resistant to CDDP compared with SGC-7901 and MGC-803 sensitive cells. The IC_50_ values of 5-FU and CDDP in the SGC-7901/5-FU were about ten times more than that in the SGC-7901 and MGC-803 cells.

### The exosomes isolated from SGC-7901 and MGC-803 cells increased the sensitivity of SGC-7901/5-FU and SGC-7901/CDDP cells to 5-FU and CDDP

**Fig. 3:**
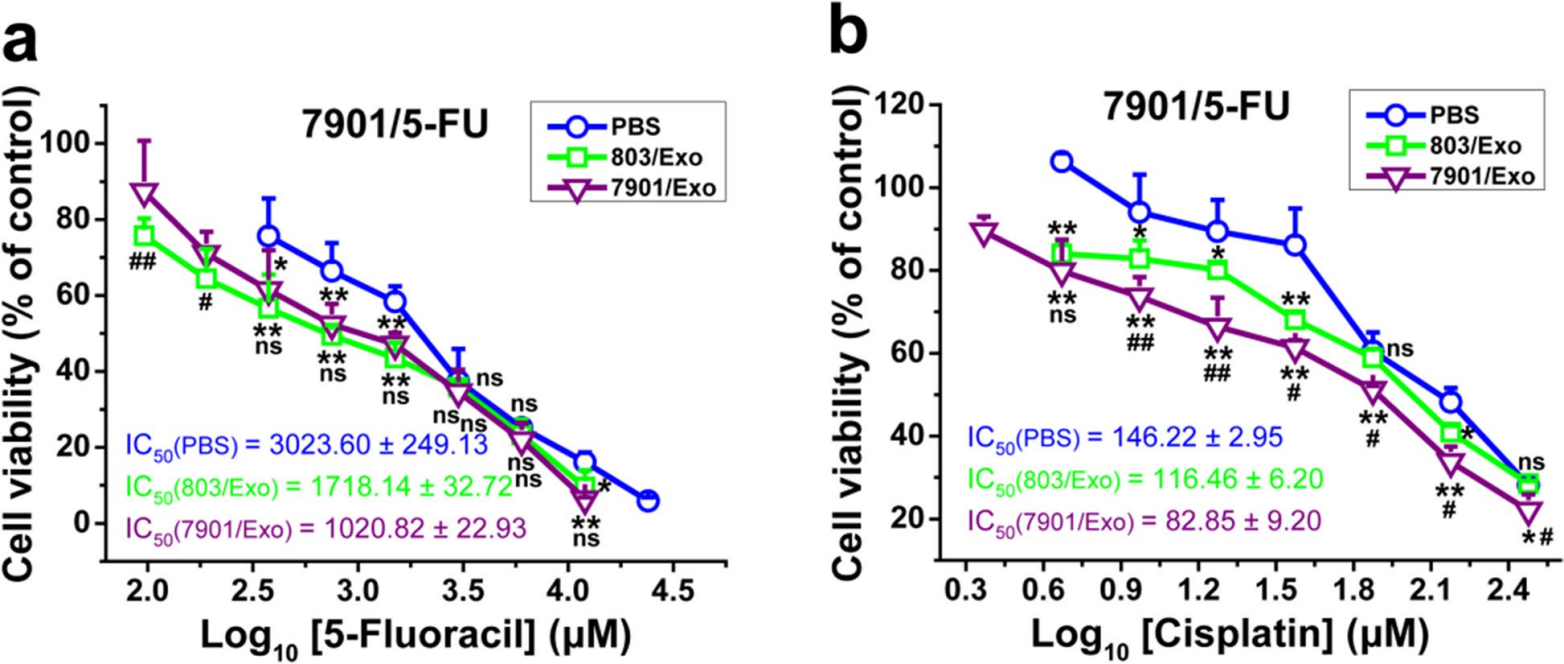
The exosomes isolated from sensitive GC cells increased drug sensitivity of drug-resistant GC cells. The cell viability of SGC-7901/5-FU cells after treated with 5-FU (**a**) or CDDP (**b**) with or without the exosomes isolated from SGC-7901 or MGC-803 cells for 48 or 24 h, respectively. Cell viability was determined by MTT assay. Cells treated with vehicle serve as a blank control. Abbreviations: Exo, exosomes. All experiments were conducted in quintuplicates and data were expressed as the mean ± SD (*n* = 5). Statistical significances were determined using one-way ANOVA followed by Dunnett’s test. ^*^P < 0.05,^**^P < 0.01, compared with the PBS control group;^*#*^*P < 0.05*,^*##*^*P < 0.01*, compared with the 803/Exo group; ns, no significance (*P > 0.05*), compared with the PBS control or 803/Exo groups

In order to explore whether exosomes could modulate the sensitivity of GC resistant cells to chemotherapy drug, we extracted the exosomes of SGC-7901 and MGC-803 sensitive cells, and treated SGC-7901/5-FU drug-resistant cells with the exosomes at a certain dose. The results showed that exosomes extracted from MGC-803 and SGC-7901 increased the sensitivity of SGC-7901/5-FU cells to 5-FU and CDDP. In the presence of MGC-803- and SGC-7901-secreted exosomes, the IC_50_ values (μM) of 5-FU in the SGC-7901/5-FU resistant cells decreased from 3023.60 to 1718.14 and 1020.82, respectively, and the IC_50_ values (μM) of CDDP in the SGC-7901/5-FU resistant cells decreased from 146.22 to 116.46 and 82.85, respectively. What’s more, the effects of exosomes from SGC-7901 cells were much stronger than those from MGC-803 cells. The IC_50_ values of 5-FU and CDDP of SGC-7901/5-FU cells treated with SGC-7901 exosomes were about 33 and 57% of those without exosomes treatment, respectively (Fig. 3).

In addition, we treated SGC-7901/CDDP drug-resistant cells with the exosomes isolated from SGC-7901 cells and the IC_50_ values of CDDP in the SGC-7901/CDDP resistant cells decreased from 169.01 μM to 89.66 μM (Additional file 1: Fig. S1). Altogether, the exosomes isolated from sensitive GC cells reversed the resistance of SGC-7901/5-FU and SGC-7901/CDDP cells to the chemotherapeutic agents.

### The SGC-7901/5-FU recipient cells absorbed exosomes from sensitive GC cells

**Fig. 4:**
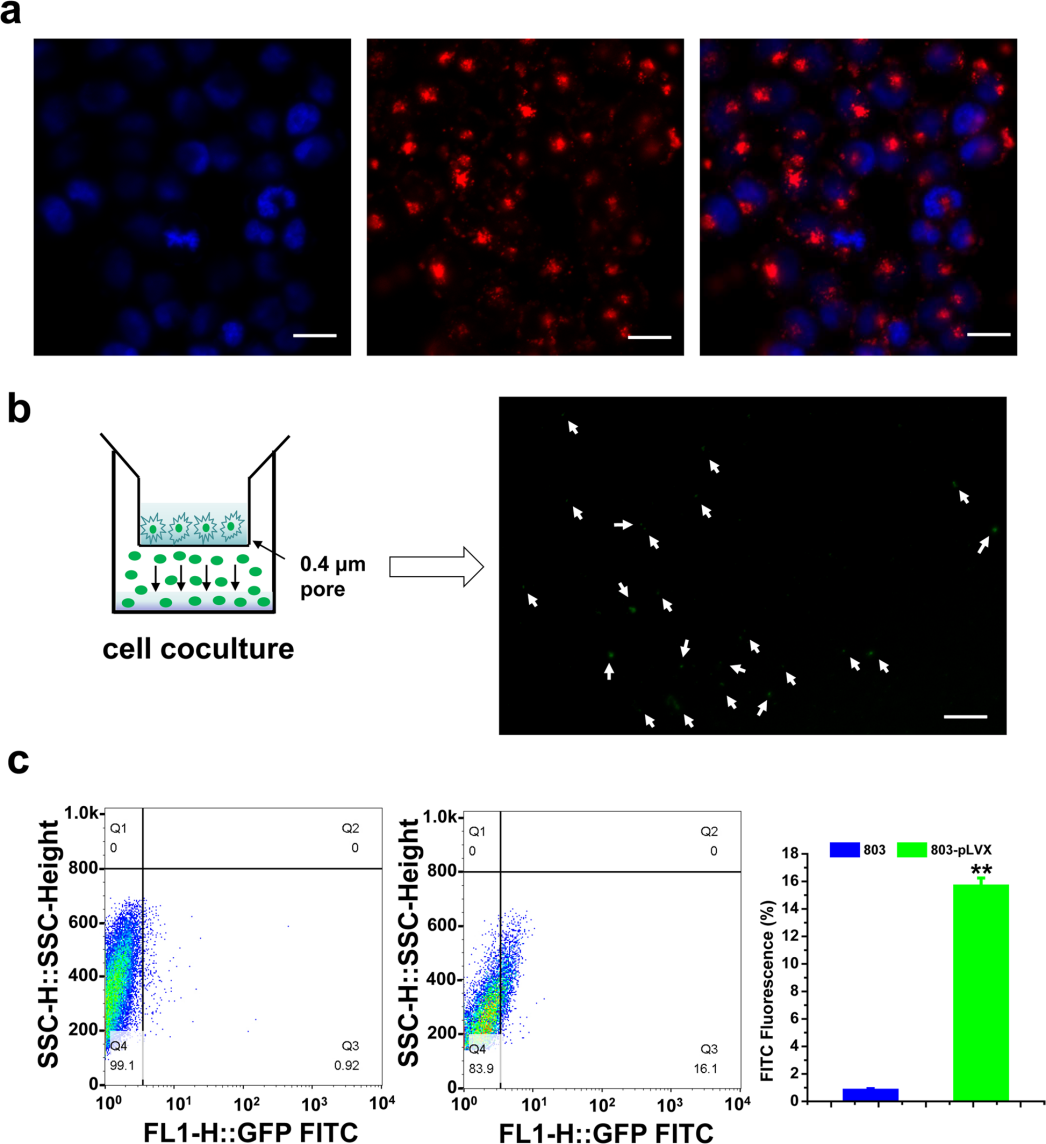
Exosomes derived from sensitive GC cells were taken up by SGC-7901/5-FU cells. **a** Confocal microscopy showed exosome internalization by SGC-7901/5-FU recipient cells after co-incubation with PKH26-labeled SGC-7901 exosomes. Red: exosomes stained with PKH26, blue: DAPI. Scale Bars = 20 μm. **b** The MGC-803-pLVX stable cells were placed in the upper chamber and coincubated with SGC-7901/5-FU cells seeded in the lower chamber in a coculture system with a 0.4 μm pore membrane. Green fluorescence was observed in the SGC-7901/5-FU recipient cells under the fluorescence microscope. Scale Bars = 35 μm. **c** The percentage of green fluorescence signals in SGC-7901/5-FU resistant GC cells were determined by flow cytometry. Data were expressed as the mean ± SD (*n* = 3). Statistical significances were determined using one-way ANOVA followed by Dunnett’s test. ^**^P < 0.01, compared with the group of MGC-803 cells

In order to visualize the absorption of exosomes, we labeled SGC-7901-secreted exosomes with PKH26, a red fluorescent tracer. After we incubated labeled exosomes with SGC-7901/5-FU cells, we observed strong red fluorescence in the cytoplasm of recipient cells and the labeled exosomes gathered around the nucleus in dots or clots under a confocal fluorescence microscope (Fig. 4a), which showed that the exosomes secreted by SGC-7901 were successfully taken up by SGC-7901/5-FU recipient cells. To visualize exosome transfer, MGC-803 cells or MGC-803-pLVX stable cells were replated in the upper chamber of a coculture system with 0.4 μm pores, which prevents direct contact between the cells and allow the transmission of exosomes but not cells. After 48 h of coculture, we also observed green fluorescence appeared in the cytoplasm of SGC-7901/5-FU cells, although the numbers of green vesicles were relatively weak (Fig. 4b). Also, compared with the group of MGC-803 normal cells, about 16% green fluorescence signal were detected by FACS analysis in MGC-803-pLVX stable cells group (Fig. 4c), which demonstrated that the exosomes labeled with green fluorescence and exosomal contents might be directly transferred from MGC-803 donor cells to SGC-7901/5-FU recipient cells. All together, these results indicated that exosomes could shuttle from the sensitive GC cells to the SGC-7901/5-FU resistant GC cells. Since exosomes could be taken up by SGC-7901/5-FU cells, we deduced that the contents of exosomes would also be uptaken by resistant cells.

### SGC-7901-secreted exosomal miR-107 reversed drug resistance of SGC-7901/5-FU cells by targeting HMGA2

**Fig. 5:**
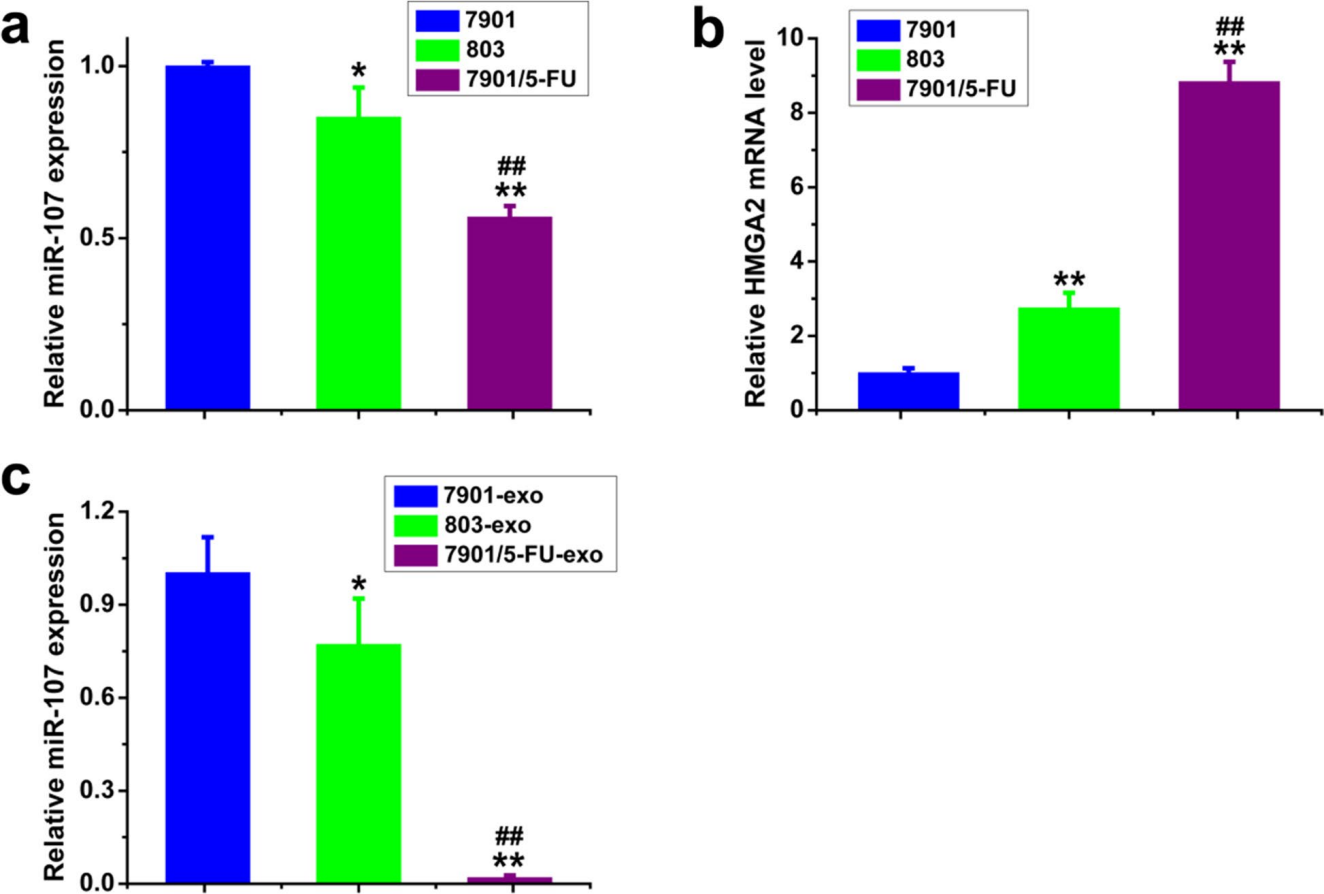
The expression levels of miR-107 and HMGA2 mRNA in SGC-7901, MGC-803 and SGC-7901/5-FU cells and exosomes were detected by qPCR. the expression levels of miR-107 (**a**) and HMGA2 mRNA (**b**) in SGC-7901, MGC-803 sensitive and SGC-7901/5-FU resistant cells; the expression levels of miR-107 (**c**) in SGC-7901, MGC-803 sensitive and SGC-7901/5-FU resistant exosomes. mRNA and miRNA levels were determined by qPCR using GAPDH and U6 as the internal control, respectively. Data were expressed as the mean ± SD (*n* = 3). Statistical significances were determined using one-way ANOVA followed by Dunnett’s test. ^*^P < 0.05, ^**^P < 0.01, compared with SGC-7901 cells/exosomes; ^*##*^*P < 0.01*, compared with MGC-803 cells/exosomes

We also tested the expression levels of miR-107 and HMGA2 mRNA in the SGC-7901, MGC-803 sensitive and SGC-7901/5-FU resistant cells and exosomes, and found that the expression levels of miR-107 in MGC-803 cells and exosomes were lower than that of SGC-7901 cells and exosomes. Moreover, the miR-107 levels in SGC-7901/5-FU resistant cells and exosomes were significantly lower than those of SGC-7901 and MGC-803 cells and exosomes (Fig. 5a and c). Simultaneously, the mRNA level of HMGA2 in MGC-803 was higher than that of SGC-7901 cells, and in SGC-7901/5-FU resistant cells, the HMGA2 mRNA level was significantly higher than that of SGC-7901 and MGC-803 cells (Fig. 5b). These results indicated that the expression of miR-107 in drug-resistant cells and exosomes were significantly different compared to sensitive cells and exosomes, and the target molecules of miR-107 may be HMGA2.

In order to study the effect of miR-107 on drug sensitivity of resistant GC cells, we transfected SGC-7901/5-FU and SGC-7901/CDDP cells with miR-107 mimic/NC and treated with 5-FU or CDDP. The results showed that miR-107 overexpression increased the sensitivity of SGC-7901/5-FU and SGC-7901/CDDP cells to 5-FU and CDDP (Additional file 1: Fig. S2). It is of note that SGC-7901/CDDP cell lines were resistant to CDDP and also cross-resistant to 5-FU compared with SGC-7901 sensitive cells (Additional file 1: Fig. S2). The IC_50_ values in the SGC-7901/5-FU cells decreased from 3668.70 μM to 1484.38 μM for 5-FU and from 131.52 μM to 80.68 μM for CDDP, respectively. The IC_50_ values in the SGC-7901/CDDP resistant cells decreased from 2718.31 μM to 1606.48 μM for 5-FU and from 236.51 μM to 89.76 μM for CDDP, respectively. Thus, we found that miR-107 from sensitive cells exosome may reverse chemotherapy drugs resistance of SGC-7901/5-FU cells.

### HMGA2 was the target gene of miR-107

**Fig. 6:**
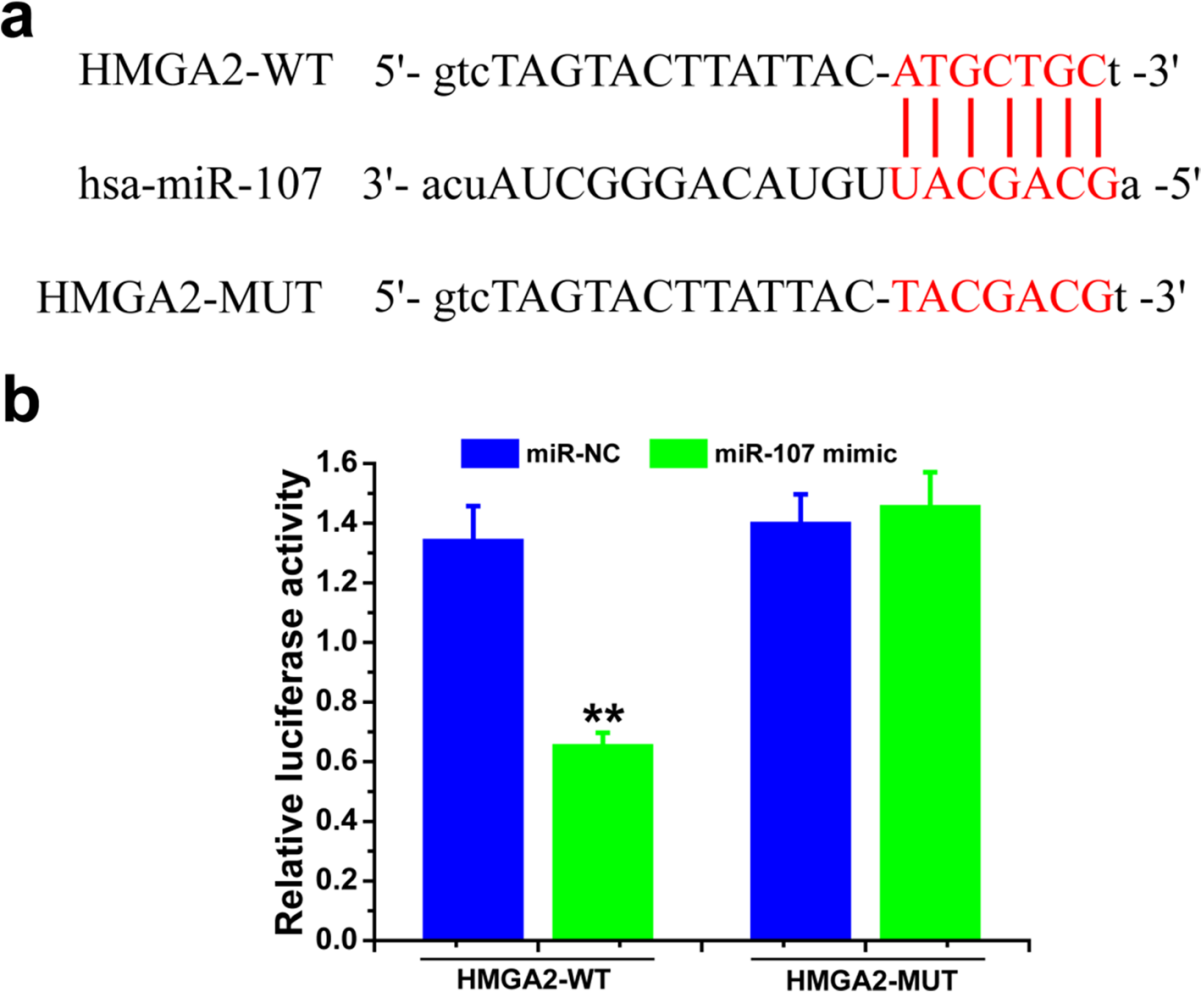
Downstream target molecular of miR-107 was HMGA2. **a** Predicted binding sites of miR-107 in HMGA2 3′-UTR. **b** Plasmid vectors of human HMGA2 3′-UTR or its mutation were transfected into 293 T cells together with miR-107 mimic or NC. Data were expressed as fold change of the luciferase activity over the control from the NC group (mean ± SD, *n* = 3). Statistical significances were determined using one-way ANOVA followed by Dunnett’s test. ^**^P < 0.01, compared with respective controls

According to the Targetscan software analysis, HMGA2 3′-UTR had a pseudo binding site with miR-107 (Fig. 6a). To verify this prediction, a luciferase reporter assay was performed. The results showed that the expression level of miR-107 in 293 T cells transfected with miR-107 mimic was 5.38 times higher than that transfected with miR-NC (Additional file 1: Fig. S3a). The overexpression of miR-107 reduced the luciferase activity in 293 T cells cotransfected with the pEZX-MT05-MT reporter plasmid containing wild type HMGA2 3′-UTR. However, there was no significant change of the luciferase activity in 293 T cells cotransfected with the pEZX-MT05-MUT reporter plasmid containing mutant HMGA2 3′-UTR (Fig. 6b). What’s more, we found that the protein expression level and mRNA level of HMGA2 were significantly up-regulated treated with miR-107 inhibitor (Figs. 9c,f and 10a). Also, we found that miR-107 overexpression downregulated the expression of HMGA2 (Additional file 1: Fig. S4). All together, these results demonstrated that HMGA2 was the target molecular of miR-107.

### The drug resistance of SGC-7901/5-FU cells was reversed via exosomes transfer of SGC-7901 cells

**Fig. 7:**
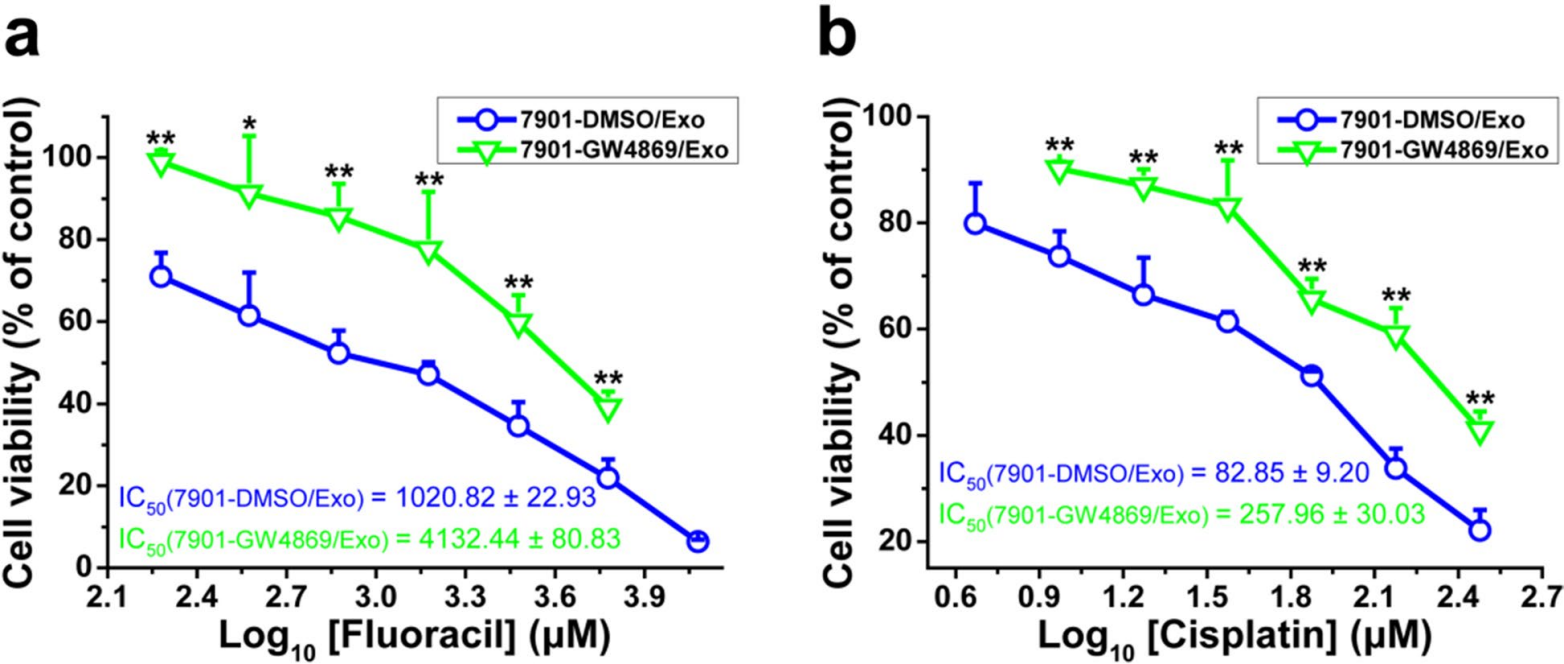
Exosomes transfer increased drug sensitivity of SGC-7901/5-FU cells. The cell viability of SGC-7901/5-FU cells after treated with 5-FU (**a**) or CDDP (**b**) combined with exosomes extracted from SGC-7901 cells (with or without the exosome inhibitor GW4869 treatment) for 48 or 24 h, respectively. Cell viability was determined by MTT assay. Cells treated with vehicle serve as a blank control. Abbreviations: Exo, exosomes. All experiments were conducted in quintuplicates and data were expressed as the mean ± SD (*n* = 5). Statistical significances were determined using one-way ANOVA followed by Dunnett’s test. ^*^P < 0.05,^**^P < 0.01, compared with the DMSO/Exo control group

In order to verified that the improved drug sensitivity of SGC-7901/5-FU cells was indeed mediated by sensitive cells-secreted exosomes, we treated SGC-7901 cells with the GW4869, which inhibits the synthesis and secretion of exosomes, and then treated SGC-7901/5-FU cells together with exosomes and 5-FU or CDDP. We found that the SGC-7901-secreted exosomes, which were treated with GW4869, could no longer increased the drug sensitivity of SGC-7901/5-FU cells to 5-FU and CDDP compared with SGC-7901-secreted exosomes, which were treated with DMSO (Fig. 7). These results showed that the drug resistance of SGC-7901/5-FU cells was reversed by exosomes transfer of SGC-7901 cells.

### Exosomal miR-107 secreted by SGC-7901 reversed drug resistance of SGC-7901/5-FU cells through targeting HMGA2

**Fig. 8:**
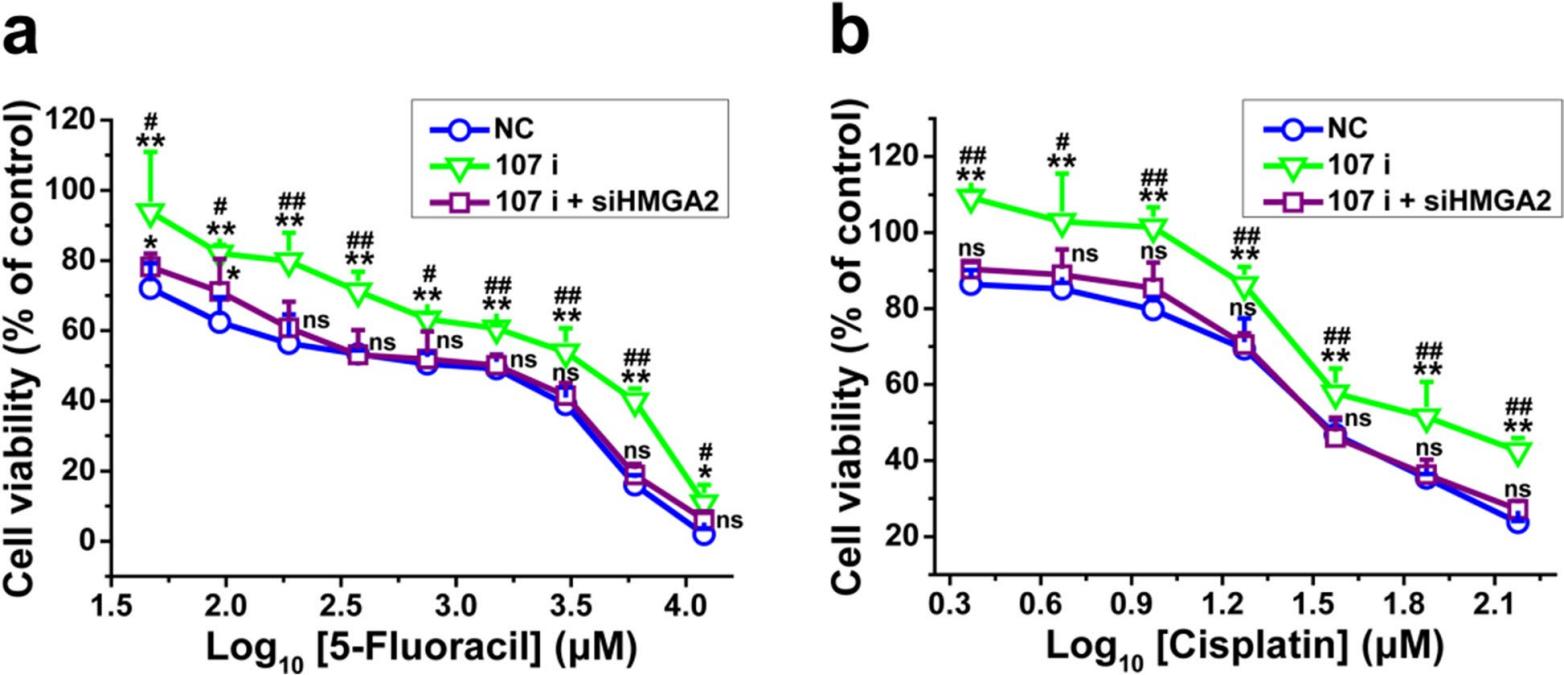
SGC-7901-secreted exosomal miR-107 reversed drug resistance of SGC-7901/5-FU cells by inhibiting HMGA2. The SGC-7901 cells were transfected with miRNA-107 inhibitor, co-transfection with miRNA-107 inhibitor and siRNA traget for HMGA2 and miRNA-107 inhibitor/siHMGA2 negative control for 24 h, and the cell viability of SGC-7901/5-FU cells after treated with different concentrations of 5-FU (**a**) or CDDP (**b**) combined with exosomes extracted from SGC-7901 (NC, 107 i, 107 i + siHMGA2) cells for 48 or 24 h, respectively. Cell viability was determined by MTT assay. Cells treated with vehicle serve as a blank control. Abbreviations: Exo, exosomes. All experiments were conducted in quintuplicates and data were expressed as the mean ± SD (*n* = 5). Statistical significances were determined using one-way ANOVA followed by Dunnett’s test. ^*^P < 0.05, ^**^P < 0.01, compared with negative control transfection group; ^*#*^*P < 0.05*, ^*##*^*P < 0.01*, compared with miRNA-107 inhibitor/siHMGA2 co-transfection group; ns, no significance (*P > 0.05*), compared with the negative control transfection and miRNA-107 inhibitor/siHMGA2 co-transfection groups

In order to evaluated whether exosomal miR-107 increased drug sensitivity in SGC7901/5-FU cells by targeting HMGA2, we transfected SGC-7901 cells with a miRNA-107 inhibitor (107 i) together with siRNA specific for HMGA2 (107i + siHMGA2) or negative control miRNA-107 inhibitor/siHMGA2 (NC). The expression levels of miR-107 and HMGA2 mRNA in SGC-7901 cells transfected with miR-107 inhibitor or siHMGA2 were 22 and 32% of those transfected with NC, respectively (Additional file 1: Fig. S3b and c). As shown in Fig. 8, SGC-7901 (NC) exosomes dramatically increased the drug sensitivity of SGC-7901/5-FU cells to 5-FU and CDDP, whereas no significant difference was seen for miR-107 silenced SGC-7901 (107 i) exosomes. What’s more, the exosomes isolated from SGC-7901 (107 i + siHMGA2) cells also reversed the resistance of SGC-7901/5-FU cells to 5-FU and CDDP. Taken together, these results suggested that the exosomes secreted by miR-107 knockdown sensitive GC cells no longer increased the sensitivity of SGC7901/5-FU cells to chemotherapy drugs 5-FU and CDDP, and reversed drug resistance can be achieved by exosomal transfer of miR-107 possibly through inhibiting HMGA2 expression in SGC-7901/5-FU cells.

### Exosomal miR-107 reversed drug resistance of SGC-7901/5-FU recipient cells through HMGA2/mTOR/P-gp pathway

**Fig. 9:**
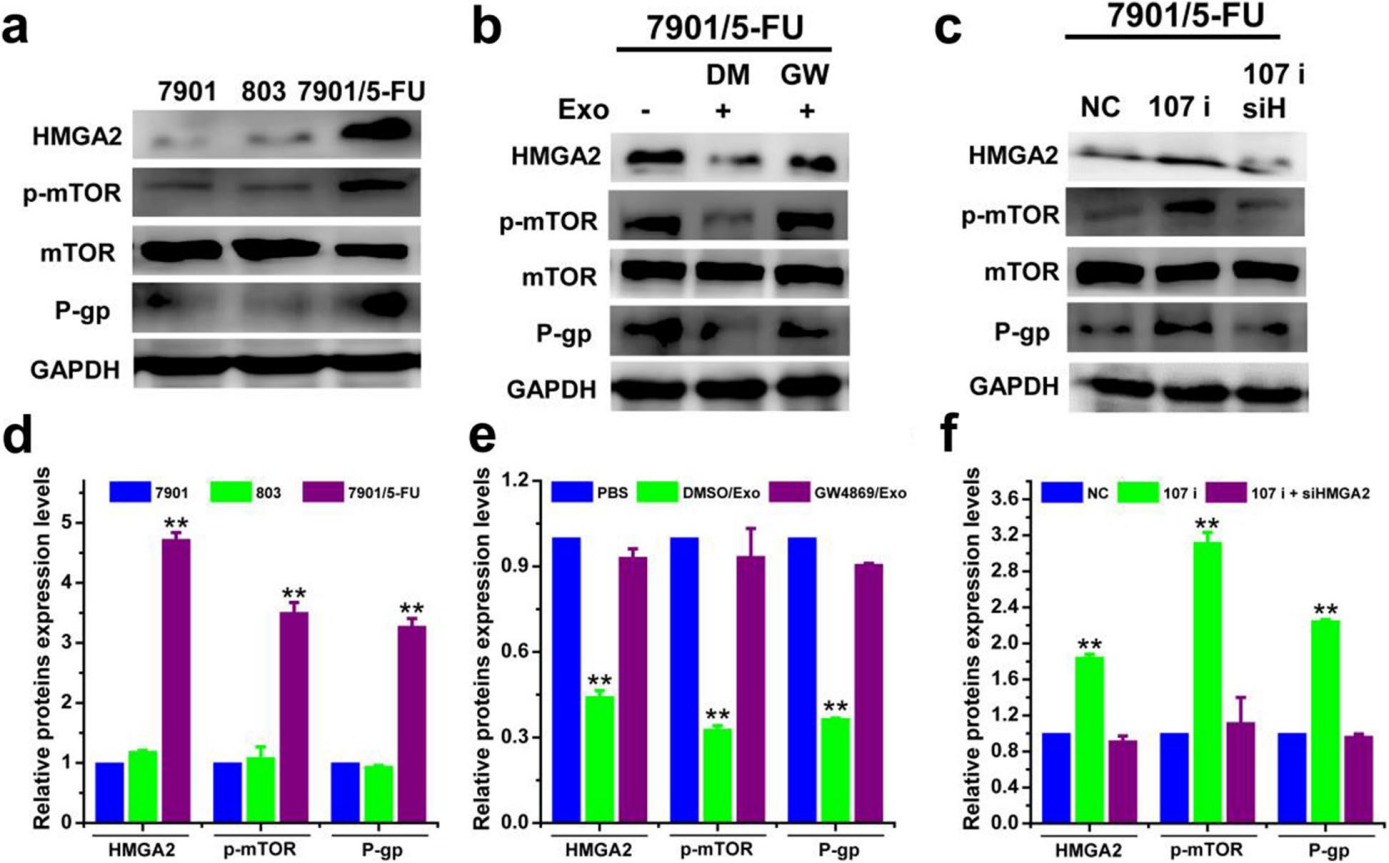
Exosomal miR-107 reversed drug resistance of SGC-7901/5-FU cells by downregulating HMGA2/mTOR/P-gp pathway. The protein expression levels of HMGA2, p-mTOR/mTOR, P-gp (**a-c**) and corresponding quantitative analysis (**d-f**) of SCG7901, MGC-803 and SCG7901/5-FU cells treated with or without exosomal inhibitor GW4869 and transfected with miR-107 inhibitor, co-transfected with miRNA-107 inhibitor/siHMGA2 or negative control were determined. The protein expression levels were detected by western blotting analysis using GAPDH as internal control. Cells treated with vehicle, or transfected with control miRNA or siRNA serve as control. Abbreviations: Exo, exosome; DM, DMSO; GW, GW4869; NC, negative control. Data in a-c are the representative of two independent experiments. Statistical significances in d-f were determined using one-way ANOVA followed by Dunnett’s test. ^**^P < 0.01, compared with the respective controls or sensitive cells

**Fig. 10:**
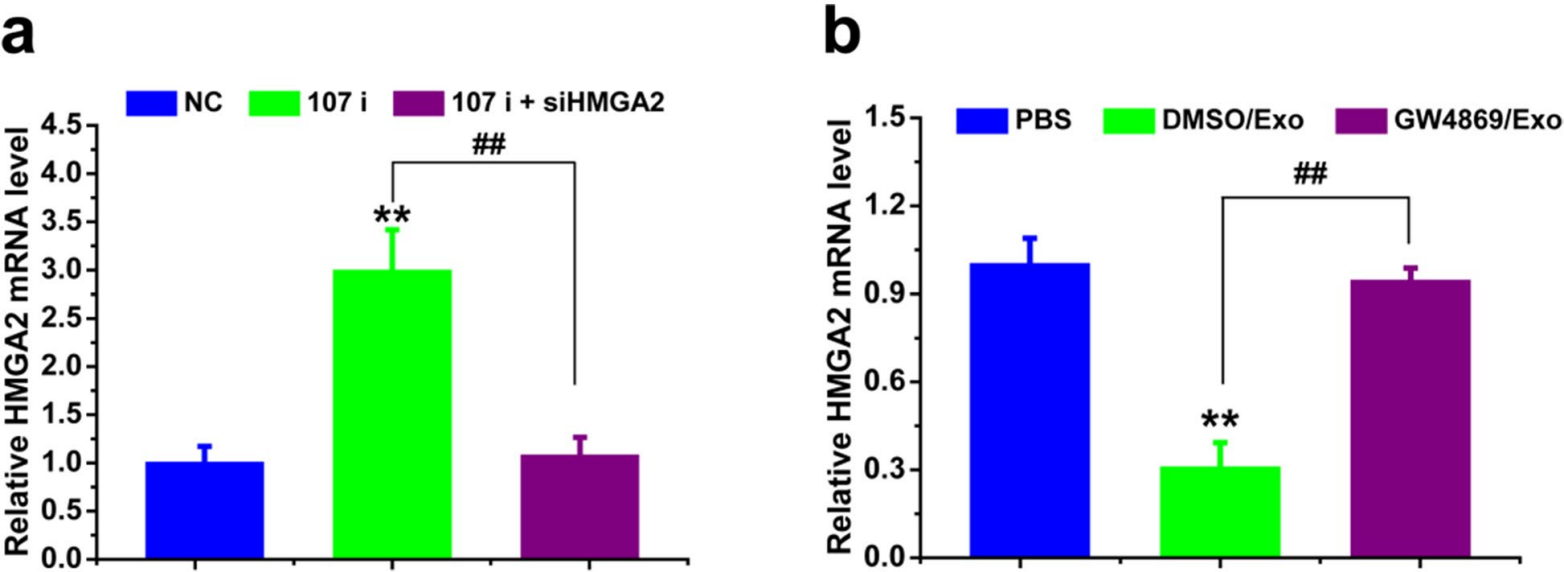
Exosomal miR-107 increased drug sensitivity of SCG-7901/5-FU cells by regulating the mRNA level of HMGA2. mRNA level of HMGA2 of SCG-7901/5-FU cells treated with exosomes isolated from SGC-7901 (transfected with NC, 107 i, 107 i + siHMGA2) cells (**a**) and treated by exosomes isolated from SGC-7901 cells (with or without exosomal inhibitor GW4869) (**b**) were detected. mRNA levels were determined by qPCR using GAPDH as the internal control. Cells treated with vehicle, or transfected with control miRNA or siRNA serve as control. Abbreviations: Exo, exosome; DM, DMSO; GW, GW4869; NC, negative control. Data were expressed as the mean ± SD (*n* = 3). Statistical significances were determined using one-way ANOVA followed by Dunnett’s test. ^**^P < 0.01, compared with the respective controls; ^*##*^*P < 0.01*, comparison between the two columns

We then sought to reveal the underlying molecular mechanism of how exosomal miR-107/HMGA2 axis reversed drug resistance of SGC-7901/5-FU cells. In our experiments, we found that the expression of HMGA2 and P-gp were significantly higher and mTOR was over-activated in SGC-7901/5-FU cells compared with the SGC-7901 and MGC-803 cells (Fig. 9a and d). As shown in Fig. 9b,c,e,f and Fig. 10, the protein expression levels of HMGA2, p-mTOR (Ser 2448), P-gp and the mRNA level of HMGA2 in SGC-7901/5-FU cells were significantly down-regulated by SGC7901 (transfected with NC or with 107 i + siHMGA2)-secreted exosomes treatment. Conversely, inhibition of exosome secretion or miR-107 knockdown in SGC-7901 exosomes increased the protein expression levels of HMGA2 p-mTOR, P-gp and the mRNA level of HMGA2 compared with control. Also, we found that miR-107 overexpression downregulated the expression of HMGA2, p-mTOR and P-gp, which indicated that miR-107 overexpression inhibited the activation of HMGA2/mTOR/P-gp pathway (Additional file 1: Fig. S4). In all, these results demonstrated that the increased sensitivity of cells to chemotherapeutic drugs by exosomal miR-107 was mediated by inhibiting the expression of HMGA2 and the activation of HMGA2/mTOR/P-gp axis.

**Fig. 11:**
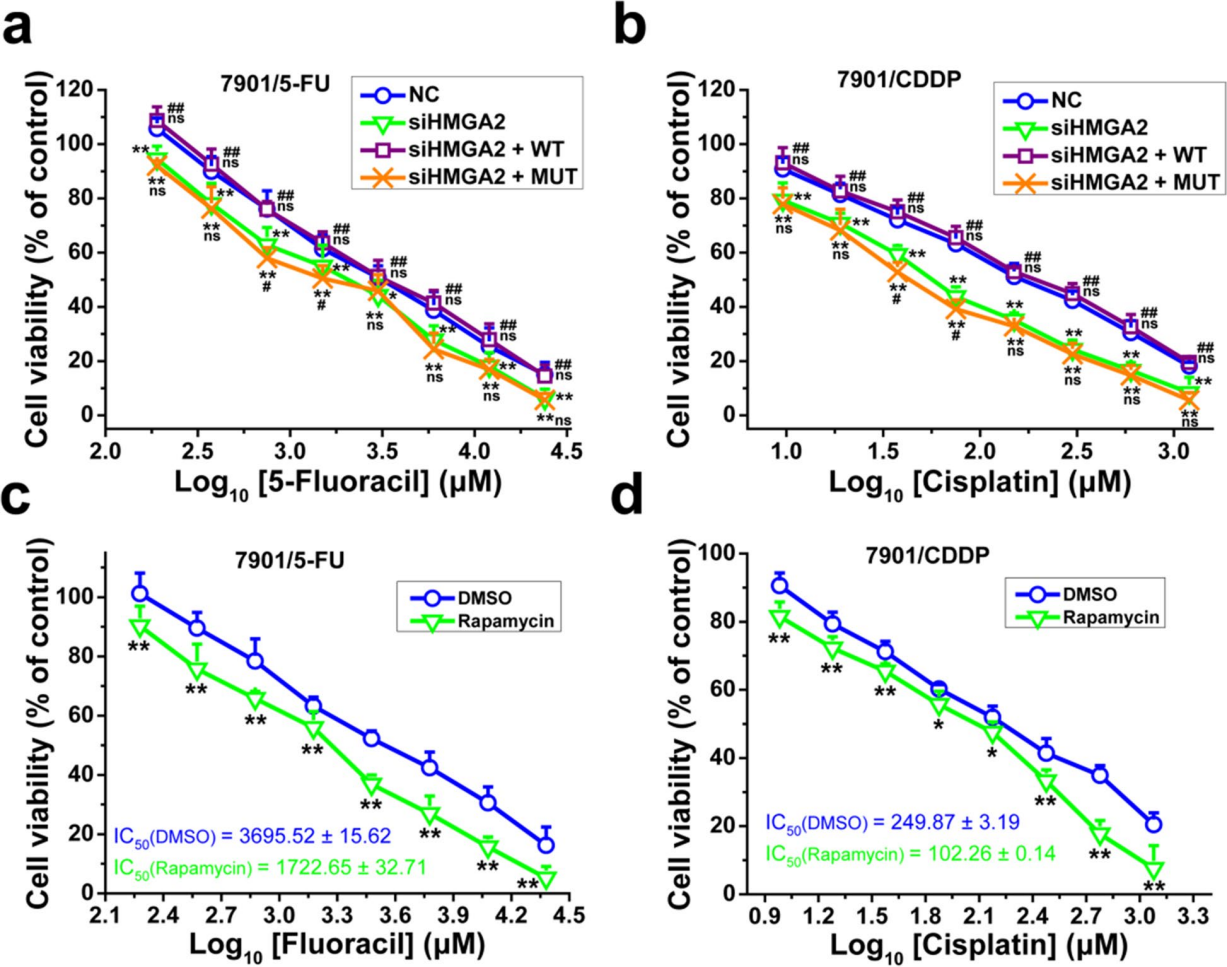
Inhibition of HMGA2/mTOR pathway increased drug sensitivity of SGC-7901/5-FU and SGC-7901/CDDP cells. The cell viability of SGC-7901/5-FU (**a** and **c**) and SGC-7901/CDDP (**b** and **d**) cells was determined after cells were transfected with siHMGA2/NC together with pEX-HMGA2-WT/MUT43 plasmid vectors (**a** and **b**) or treated with or without rapamycin (10 μM) (**c** and **d**) and then treated with 5-FU (**a** and **c**) or CDDP (**b** and **d**) for 48 or 24 h, respectively. Cell viability was determined by MTT assay. Cells treated with vehicle serve as a blank control. All experiments were conducted in quintuplicates and data were expressed as the mean ± SD (*n* = 5). Statistical significances were determined using one-way ANOVA followed by Dunnett’s test. ^*^P < 0.05, ^**^P < 0.01, compared with negative control transfection/DMSO group; ^*#*^*P < 0.05*, ^*##*^*P < 0.01*, compared with siHMGA2 transfection group; ns, no significance (*P > 0.05*), compared with the negative control transfection and siHMGA2 transfection groups

To further study the role of HMGA2/mTOR pathway on resistant GC cells to chemotherapy drugs, we transfected SGC-7901/5-FU and SGC-7901/CDDP cells with siHMGA2 with or without pEX-HMGA2-WT or pEX-HMGA2-MUT43. The expression level of HMGA2 in SGC-7901/5-FU cells transfected with siRNA for HMGA2 was only 26% of control (Additional file 1: Fig. S5a), and the expression level of HMGA2 in SGC-7901/5-FU cells transfected with HMGA2-WT was 2.68 times of control. The HMGA2 deletion mutant expressed by pEX-HMGA2-MUT43 was too small to detect (Additional file 1: Fig. S5b). The HMGA2 mutant overexpression with HMGA2 knockdown increased 5-FU and CDDP sensitivity of SGC-7901/5-FU and SGC-7901/CDDP cells. However, HMGA2-WT overexpression with HMGA2 knockdown could not increase the sensitivity of resistant cells (Fig. 11a-b). Meanwhile, we found that inhibiting mTOR activity by rapamycin also could increase sensitivity of SGC-7901/5-FU and SGC-7901/CDDP cells (Fig. 11c-d). Altogether, these results demonstrated that inhibiting HMGA2/mTOR pathway improved sensitivity of resistant GC cells to chemotherapy drugs, and HMGA2/mTOR pathway plays important roles in drug resistance.

## Discussion

**Fig. 12:**
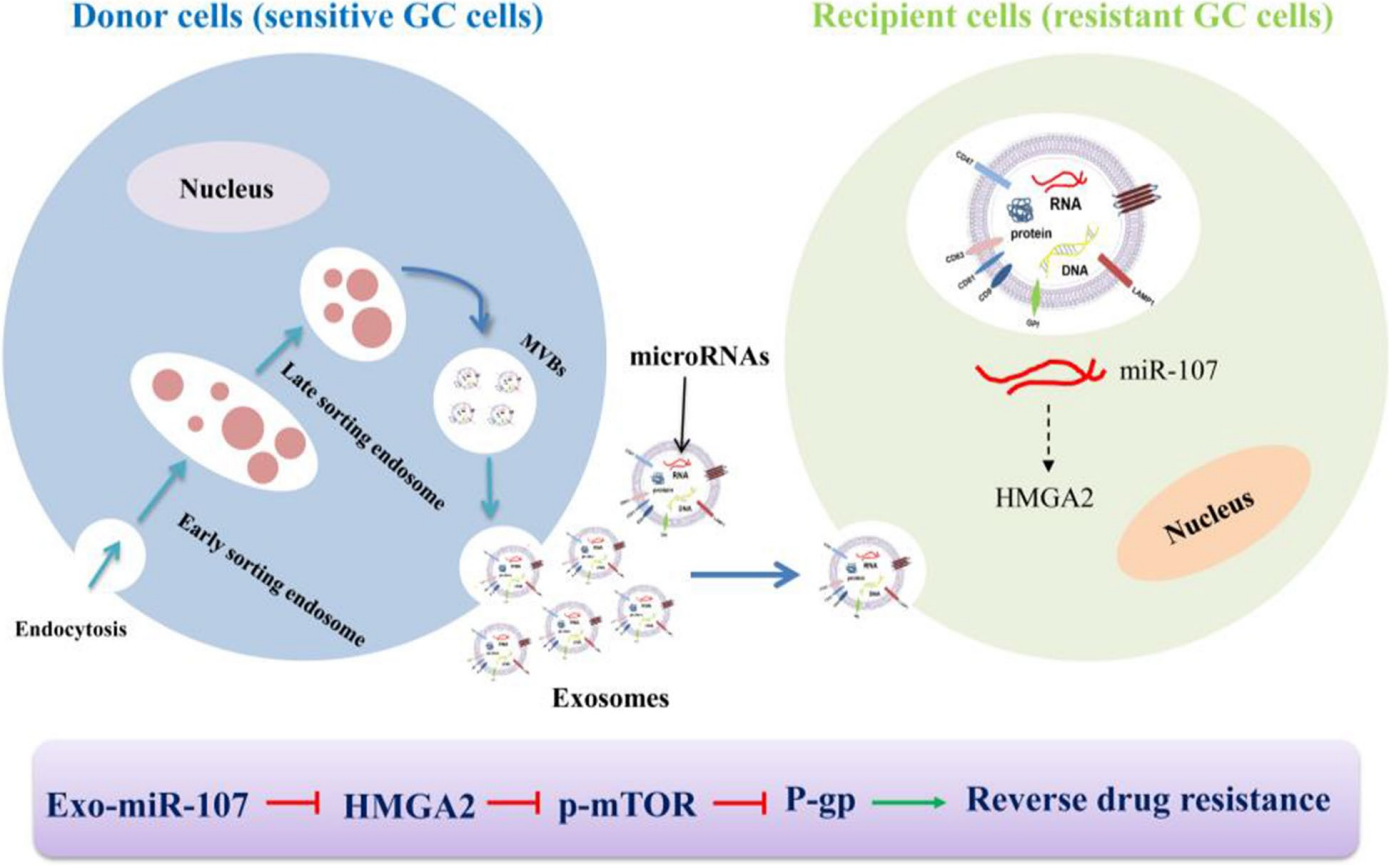
The possible mechanism of how exosomal miR-107 reversed chemotherapeutic drug resistance of SGC-7901/5-FU cells. Exosomal miR-107 was transmitted from sensitive GC cells to recipient resistant GC cells, and drug sensitivity of SGC-7901/5-FU cells was improved through inhibiting HMGA2/mTOR/P-gp signal

Chemotherapy resistance seriously affects the treatment of GC. In this study, we showed that exosomes derived from sensitive GC cells could increase the sensitivity of resistant GC cells to chemotherapeutic drugs. The reversal effects were mediated by exosomal miR-107 through targeting HMGA2 and regulating the activation of HMGA2/mTOR/P-gp pathway (Fig. 12).

Exosomes are a subgroup of extracellular vesicles, which serve as a carrier of cell contents (proteins, nucleic acid, lipids). In the past few years, it has been revealed that exosomes play an important role in mediating communication between cells, and its contents also play a certain role in mediating cancer cells resistance [23]. There are four ways for exosomes to mediate cell communication, which including 1, exosomes recognize the surface receptors by ligand-receptor binding; 2, exosomes directly fuse with target cells to achieve receptor transfer; 3, exosomes fuse with target cells and release content proteins; 4, exosomes transfer genetic information (mRNA, non-coding RNA, DNA) to target cells [24]. The interaction of exosomes and targets cells was a crucial step to induce biological effect. Thus we also used a variety of methods, such as PKH26 staining, transwell and flow cytometry, to prove that exosomes secreted by sensitive GC cells were uptaken by resistant GC cells. And the inhibitor GW4689, which inhibits the synthesis and release of exosomes, was used to prove that the exosomes from sensitive GC cells could reverse drug resistance of SGC-7901/5-FU cells.

There have been many reports showing that the regulatory role of exosomal miRNA could regulate chemotherapy resistance of cancer cells. For example, exosomal microRNA-32-5p induces multidrug resistance in hepatocellular carcinoma [25], macrophages derived exosomal miR-223 induced a chemoresistant phenotype of epithelial ovarian cancer cells through exosomal miR-223/PTEN-PI3K/AKT signaling pathway [26]. In GC cells, the MGC-803 sensitive cells became resistant to paclitaxel because exosomal miRNA-155-5p inhibited the expressions of GATA3 and TP53INP1 [27]. MGC803/CDDP-derived exosomes enhance CDDP resistance of MGC803 recipient cells via exosomal delivery of miR-500a-3p [28]. Although a variety of exosomal miRNAs have been reported to regulate chemotherapeutics sensitivity of cancer cells, the effect of exosomal miRNA-107 on the drug resistance of GC cells has not been reported. In our study, we first found that exosomal miR-107 from sensitive GC cells (SGC-7901 and MGC-803 cells) could increase the sensitivity of two resistant GC cell lines SGC-7901/5FU and SGC-7901/CDDP to chemotherapy drugs 5-FU and CDDP. Results from multiple sensitive and resistant GC cell lines support the notion that exosomes isolated from sensitive GC cells could reverse the drug resistance. Thus, exosomal miR-107 might provide a new target for improving the drug sensitivity of GC patients.

Some reports demonstrated that HMGA2 also had a certain effect on the chemotherapeutics sensitivity of cancer cells. HMGA2 regulated the progression and the sensitivity of acute myeloid leukemia to doxorubicin by activating the Wnt/β-catenin signaling pathway [29]. miR-26a could regulate the resistance of human non-small cell lung cancer to cisplatin by regulating the expression of HMGA2 through the E2F1-Akt pathway [30]. In GC cells, HMGA2 might be a potential target molecule of miR-33b-5p, and up-regulation of miR-33b-5p reduced the expression of HMGA2, inhibited the growth of GC cells, and improved the sensitivity of GC cells to chemotherapy drugs [31]. Some literature suggested that HMGA2 might be the target of miRNA-107, and miRNA-107 could inhibit the proliferation of liver cancer cells by targeting HMGA2 mRNA 3′-UTR [32]. LncRNA LINC00152 promoted the progression of glioblastoma by targeting miR-107 and regulating the expression of HMGA2 [33]. Consistently, in our study, we verified that HMGA2 was the target molecular of exosomal miR-107 using luciferase reporter assay, western blotting and qPCR assay. And exosomal miR-107 improved the sensitivity of resistant GC cells to chemotherapy drugs by targeting HMGA2.

Some studies indicated that HMGA2 could regulate the downstream mTOR signal [34]. Tan L et al. reported that aberrant expression of HMGA2 induced acute myeloid leukemia cell proliferation through the PI3K/Akt/mTOR signaling pathway [35]. miR-590 suppressed proliferation and induced apoptosis of pancreatic cancer by targeting HMGA2 and inhibiting the phosphorylation of mTOR [36]. It is well known that a major mechanism of resistance in cancer cells is the overexpression of P-gp, also known as multidrug resistance protein 1 (MDR1) or ABCB1, which belong to ATP-binding cassette (ABC) transporters and could efflux the chemotherapeutic agents out of cells [37]. Some studies showed that mTOR signal could regulate the expression of P-gp and affect the drug resistance of cancer cells [38–40]. In this study, we also found that the expression of P-gp could be regulated by mTOR signal, and exosomal miR-107 regulated the expression of P-gp mediated by HMGA2 and the activity of mTOR signal.

## Conclusion

In summary, our study reveals that exosome-transmitted miR-107 increased the sensitivity of resistant GC cells to the chemotherapeutic drugs. Mechanistically, the expression of target molecule HMGA2 and the activity of HMGA2/mTOR/P-gp signal were downregulated by horizontal transfer of exosomal miR-107. Therefore, we propose that exosomal miR-107 might be used as a potential diagnostic biomarker and therapeutic target for gastric cancers.

## Supplementary Information

**Additional file 1: Figure S1**. The exosomes isolated from SGC-7901 cells increased drug sensitivity of SGC-7901/CDDP cells. The cell viability of SGC-7901/CDDP cells was determined after cells were treated with CDDP with or without the exosomes isolated from SGC-7901 for 24 h. Cell viability was determined by MTT assay. Cells treated with vehicle serve as a blank control. Abbreviations: Exo, exosomes. All experiments were conducted in quintuplicates and data were expressed as the mean ± SD (*n* = 5). Statistical significances were determined using one-way ANOVA followed by Dunnett’s test. ^**^P < 0.01, compared with the PBS control group. **Figure S2**. miR-107 overexpression increased drug sensitivity of SGC-7901/5-FU and SGC-7901/CDDP cells. The cell viability of SGC-7901/5-FU (a and b) and SGC-7901/CDDP (c and d) cells was determined after cells were transfected with miR-107 mimic or NC and treated with 5-FU (a and c) or CDDP (b and d) for 48 or 24 h, respectively. Cell viability was determined by MTT assay. Cells treated with vehicle serve as a blank control. All experiments were conducted in quintuplicates and data were expressed as the mean ± SD (n = 5). Statistical significances were determined using one-way ANOVA followed by Dunnett’s test. ^**^P < 0.01, compared with the control group. **Figure S3**. The expression levels of miR-107 and HMGA2 mRNA in 293 T and SGC-7901 cells were detected. a The expression level of miR-107 in 293 T cells transfected with miR-107 mimic and miR-NC. b The expression level of miR-107 in SGC-7901 cells transfected with miR-107 inhibitor and miR-NC. c The mRNA level of HMGA2 in SGC-7901 cells transfected with siHMGA2 and NC. mRNA and miRNA levels were determined by qPCR using GAPDH and U6 as the internal control, respectively. Data were expressed as the mean ± SD (*n* = 3). Statistical significances were determined using one-way ANOVA followed by Dunnett’s test. ^**^P < 0.01, compared with the respective controls. **Figure S4**. miR-107 overexpression downregulated HMGA2/mTOR/P-gp pathway. The protein expression levels of HMGA2, p-mTOR/mTOR, P-gp (a) and corresponding quantitative analysis (b) in SCG7901/5-FU cells transfected with miR-107 mimic or NC were determined. The protein expression levels were detected by western blotting analysis using GAPDH as internal control. Cells transfected with control miRNA serve as control. Statistical significances in b were determined using one-way ANOVA followed by Dunnett’s test. ^**^P < 0.01, compared with the control. **Figure S5**. The expression level of HMGA2 in SGC-7901/5-FU cells. a The expression level of HMGA2 in SGC-7901/5-FU cells transfected with siRNA target for HMGA2. b The expression level of HMGA2 in SGC-7901/5-FU cells transfected with HMGA2-MUT43, HMGA2-WT vectors. The protein expression levels were detected by western blotting analysis using GAPDH as internal control. Abbreviations: NC: negative control; H-MUT, pEX-HMGA2-MUT43; H-WT, pEX-HMGA2-WT. The data represents the density of bands.

**Additional file 2.**

**Additional file 3.**

## Data Availability

All data generated or analysed during this study are included in this published article and its supplementary information files.

## Abbreviations

GC: Gastric cancer
TEM: Transmission electron microscopy
DLS: Dynamic light scattering
RT-qPCR: Real-time quantitative PCR
aa: amino acid
CDDP: cisplatin
HMGA2: High mobility group A2
P-gp: P-glycoprotein
Gluc: Gaussia luciferase
SeAP: secreted alkaline phosphatase
MDR1: Multidrug resistance protein 1
ABC: ATP-binding cassette
NC: Negative control
