# Supplementary material for: Exosomal microRNA-107 reverses chemotherapeutic drug resistance of gastric cancer cells through HMGA2/mTOR/P-gp pathway
Source: BMC Cancer. 2021 Dec 2;21:1290. doi: 10.1186/s12885-021-09020-y (PMC8638432; doi:10.1186/s12885-021-09020-y)
Supplement: Supplementary file 1 — Additional file 1: Figure S1. The exosomes isolated from SGC-7901 cells increased drug sensitivity of SGC-7901/CDDP cells. The cell viability of SGC-7901/CDDP cells was determined after cells were treated with CDDP with or without the exosomes isolated from SGC-7901 for 24 h. Cell viability was determined by MTT assay. Cells treated with vehicle serve as a blank control. Abbreviations: Exo, exosomes. All experiments were conducted in quintuplicates and data were expressed as the mean ± SD (n = 5). Statistical significances were determined using one-way ANOVA followed by Dunnett’s test. **P < 0.01, compared with the PBS control group. Figure S2. miR-107 overexpression increased drug sensitivity of SGC-7901/5-FU and SGC-7901/CDDP cells. The cell viability of SGC-7901/5-FU (a and b) and SGC-7901/CDDP (c and d) cells was determined after cells were transfected with miR-107 mimic or NC and treated with 5-FU (a and c) or CDDP (b and d) for 48 or 24 h, respectively. Cell viability was determined by MTT assay. Cells treated with vehicle serve as a blank control. All experiments were conducted in quintuplicates and data were expressed as the mean ± SD (n = 5). Statistical significances were determined using one-way ANOVA followed by Dunnett’s test. **P < 0.01, compared with the control group. Figure S3. The expression levels of miR-107 and HMGA2 mRNA in 293 T and SGC-7901 cells were detected. a The expression level of miR-107 in 293 T cells transfected with miR-107 mimic and miR-NC. b The expression level of miR-107 in SGC-7901 cells transfected with miR-107 inhibitor and miR-NC. c The mRNA level of HMGA2 in SGC-7901 cells transfected with siHMGA2 and NC. mRNA and miRNA levels were determined by qPCR using GAPDH and U6 as the internal control, respectively. Data were expressed as the mean ± SD (n = 3). Statistical significances were determined using one-way ANOVA followed by Dunnett’s test. **P < 0.01, compared with the respective controls. Figure S4. miR-107 overexpression [file 12885_2021_9020_MOESM1_ESM.docx]

**Additional file 1: Figure S1**

**
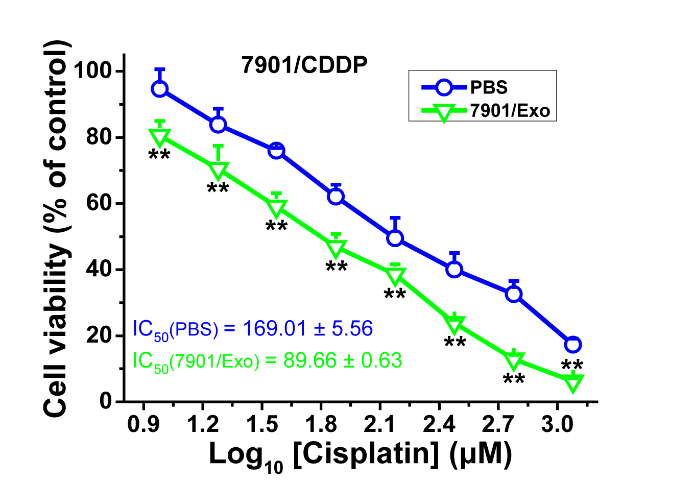
**

**Fig. S1** The exosomes isolated from SGC-7901 cells increased drug sensitivity of SGC-7901/CDDP cells. The cell viability of SGC-7901/CDDP cells was determined after cells were treated with CDDP with or without the exosomes isolated from SGC-7901 for 24 h. Cell viability was determined by MTT assay. Cells treated with vehicle serve as a blank control. Abbreviations: Exo, exosomes. All experiments were conducted in quintuplicates and data were expressed as the mean ± SD (n = 5). Statistical significances were determined using one-way ANOVA followed by Dunnett’s test. *^**^P < 0.01*, compared with the PBS control group.

**Additional file 1: Figure S2**


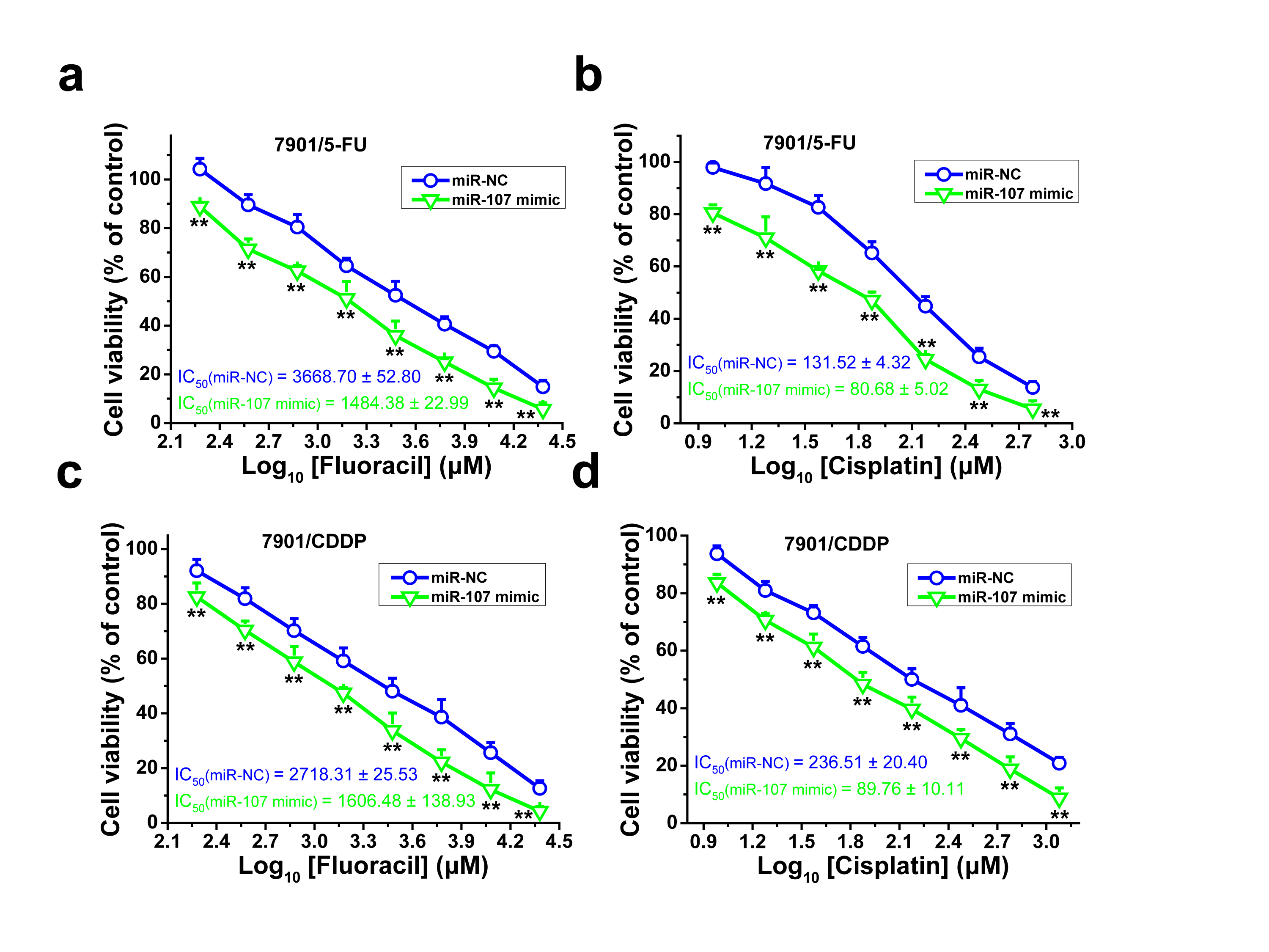


**Fig. S2** miR-107 overexpression increased drug sensitivity of SGC-7901/5-FU and SGC-7901/CDDP cells. The cell viability of SGC-7901/5-FU (**a** and **b**) and SGC-7901/CDDP (**c** and **d**) cells was determined after cells were transfected with miR-107 mimic or NC and treated with 5-FU (**a** and **c**) or CDDP (**b** and **d**) for 48 or 24 h, respectively. Cell viability was determined by MTT assay. Cells treated with vehicle serve as a blank control. All experiments were conducted in quintuplicates and data were expressed as the mean ± SD (n = 5). Statistical significances were determined using one-way ANOVA followed by Dunnett’s test. *^**^P < 0.01*, compared with the control group.

**Additional file 1: Figure S3**

**
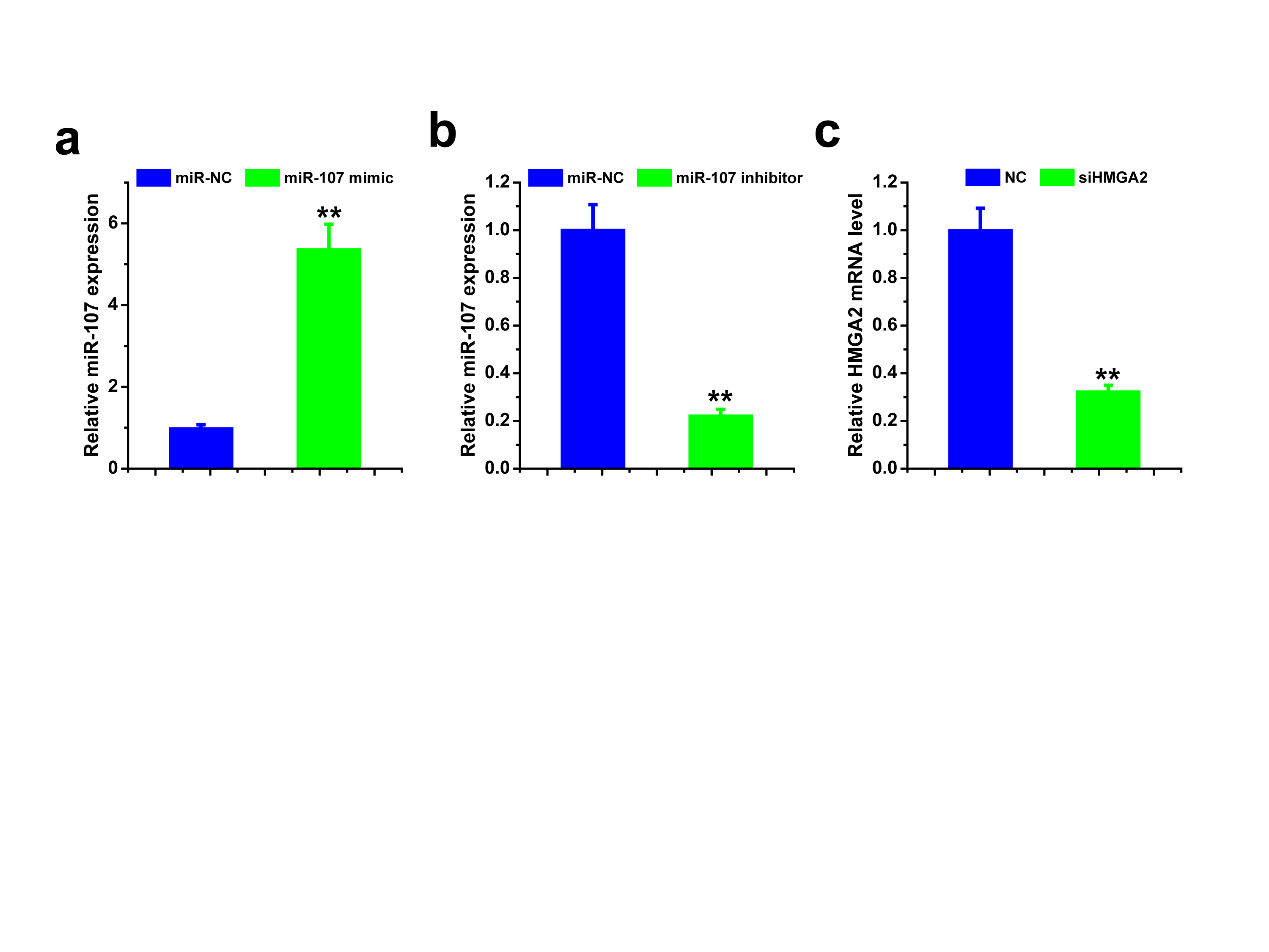
**

**Fig. S3** The expression levels of miR-107 and HMGA2 mRNA in 293T and SGC-7901 cells were detected. **a** The expression level of miR-107 in 293T cells transfected with miR-107 mimic and miR-NC. **b** The expression level of miR-107 in SGC-7901 cells transfected with miR-107 inhibitor and miR-NC. **c** The mRNA level of HMGA2 in SGC-7901 cells transfected with siHMGA2 and NC. mRNA and miRNA levels were determined by qPCR using GAPDH and U6 as the internal control, respectively. Data were expressed as the mean ± SD (n = 3). Statistical significances were determined using one-way ANOVA followed by Dunnett’s test. *^**^P < 0.01*, compared with the respective controls.

**Additional file 1: Figure S4**


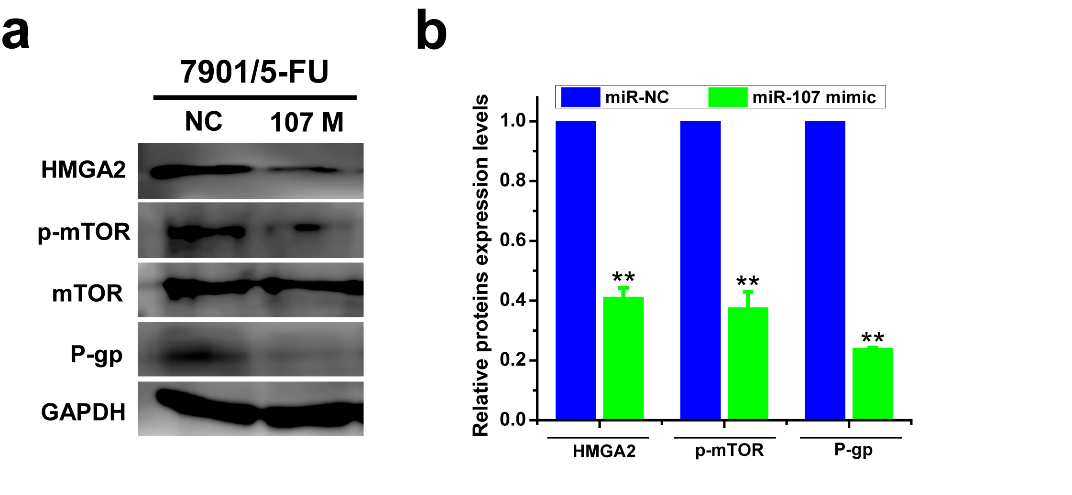


**Fig. S4** miR-107 overexpression downregulated HMGA2/mTOR/P-gp pathway. The protein expression levels of HMGA2, p-mTOR/mTOR, P-gp (**a**) and corresponding quantitative analysis (**b**) in SCG7901/5-FU cells transfected with miR-107 mimic or NC were determined. The protein expression levels were detected by western blotting analysis using GAPDH as internal control. Cells transfected with control miRNA serve as control. Statistical significances in **b** were determined using one-way ANOVA followed by Dunnett’s test. *^**^P < 0.01*, compared with the control.

**Additional file 1: Figure S5**

**
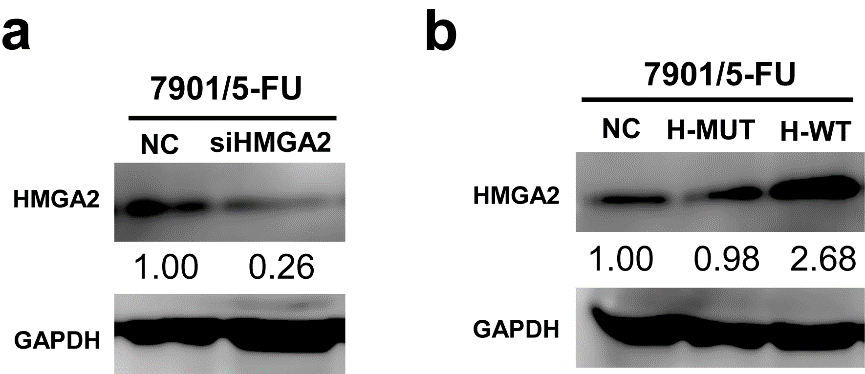
**

**Fig. S5** The expression level of HMGA2 in SGC-7901/5-FU cells. **a** The expression level of HMGA2 in SGC-7901/5-FU cells transfected with siRNA target for HMGA2. **b** The expression level of HMGA2 in SGC-7901/5-FU cells transfected with HMGA2-MUT43, HMGA2-WT vectors. The protein expression levels were detected by western blotting analysis using GAPDH as internal control. Abbreviations: NC: negative control; H-MUT, pEX-HMGA2-MUT43; H-WT, pEX-HMGA2-WT. The data represents the density of bands.
