# Supplementary material for: Exosomal microRNA-107 reverses chemotherapeutic drug resistance of gastric cancer cells through HMGA2/mTOR/P-gp pathway
Source: BMC Cancer. 2021 Dec 2;21:1290. doi: 10.1186/s12885-021-09020-y (PMC8638432; doi:10.1186/s12885-021-09020-y)

**Fig1 b-S/exo**


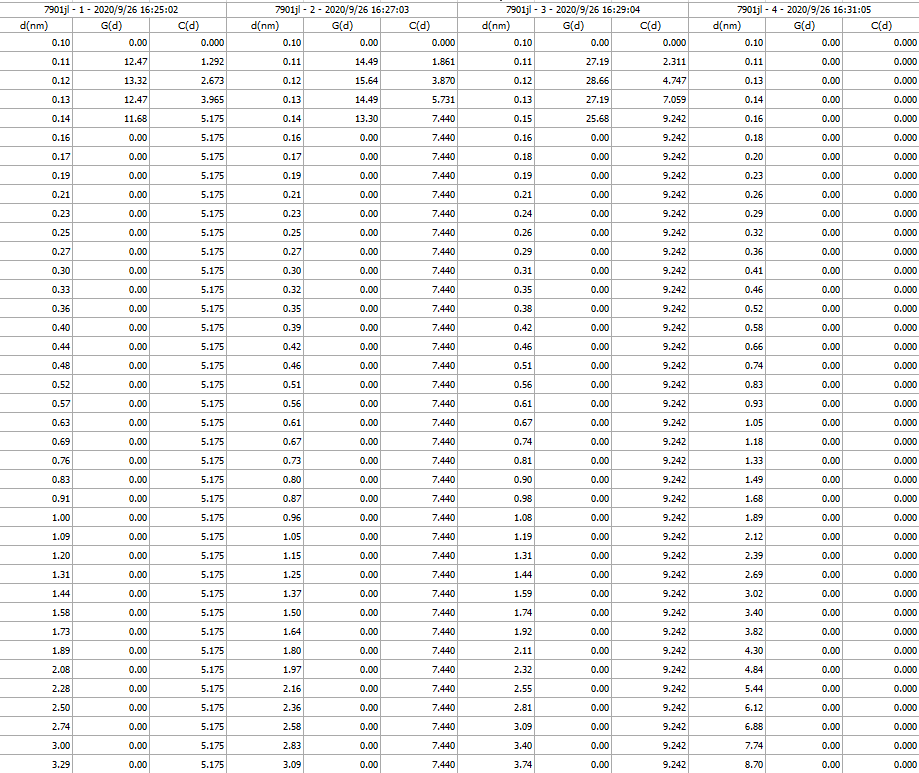


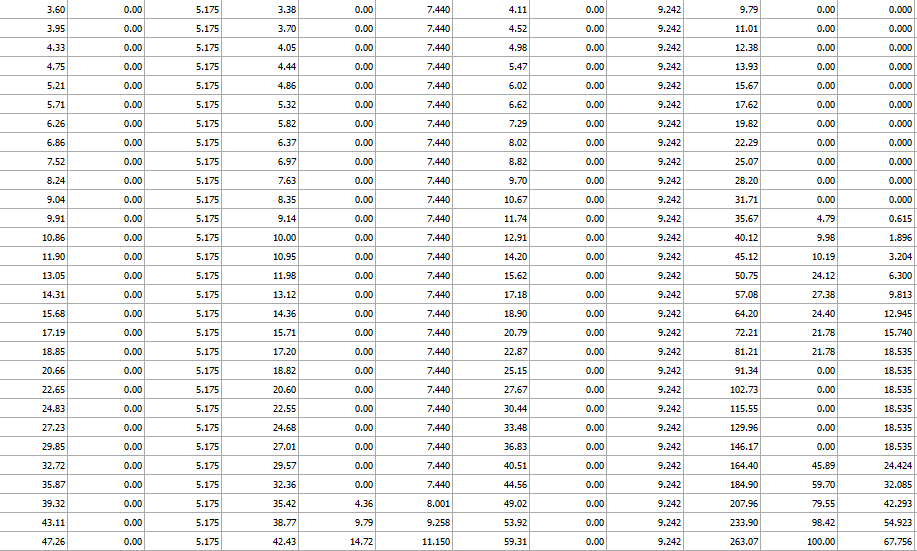


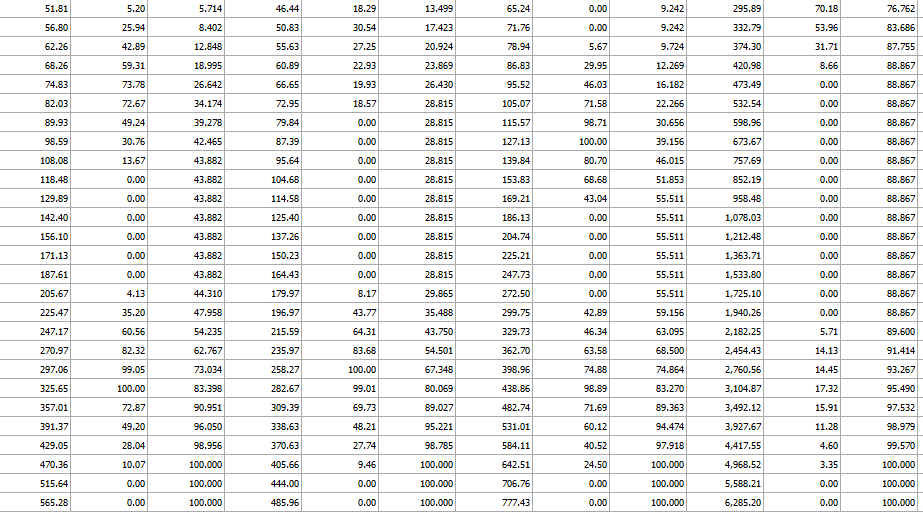


**Fig1 b-R/exo**


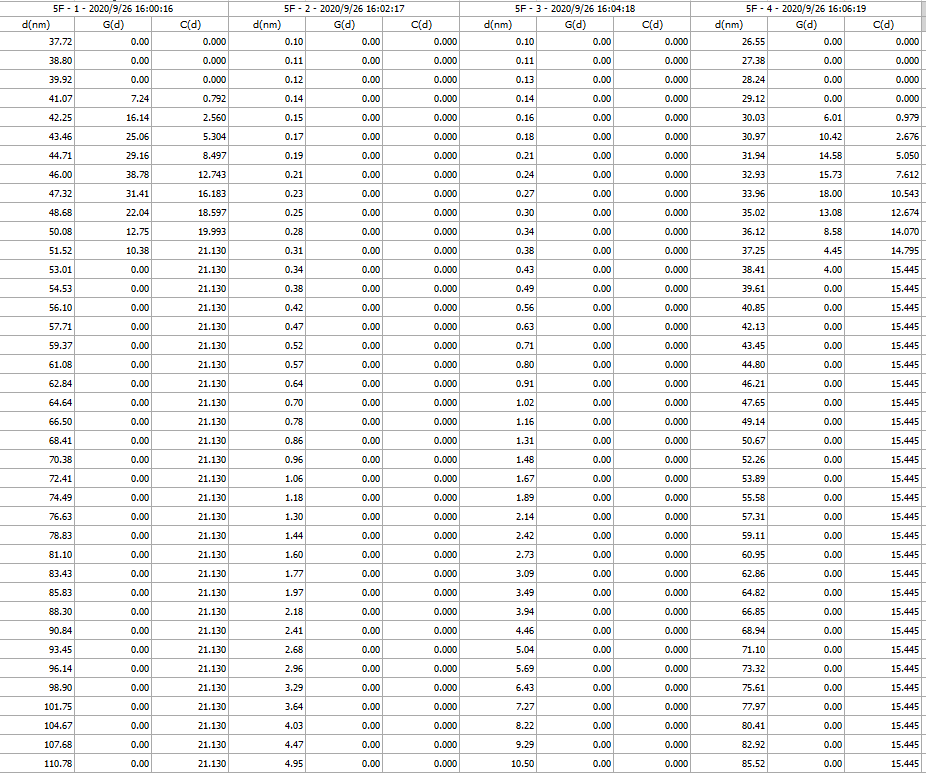


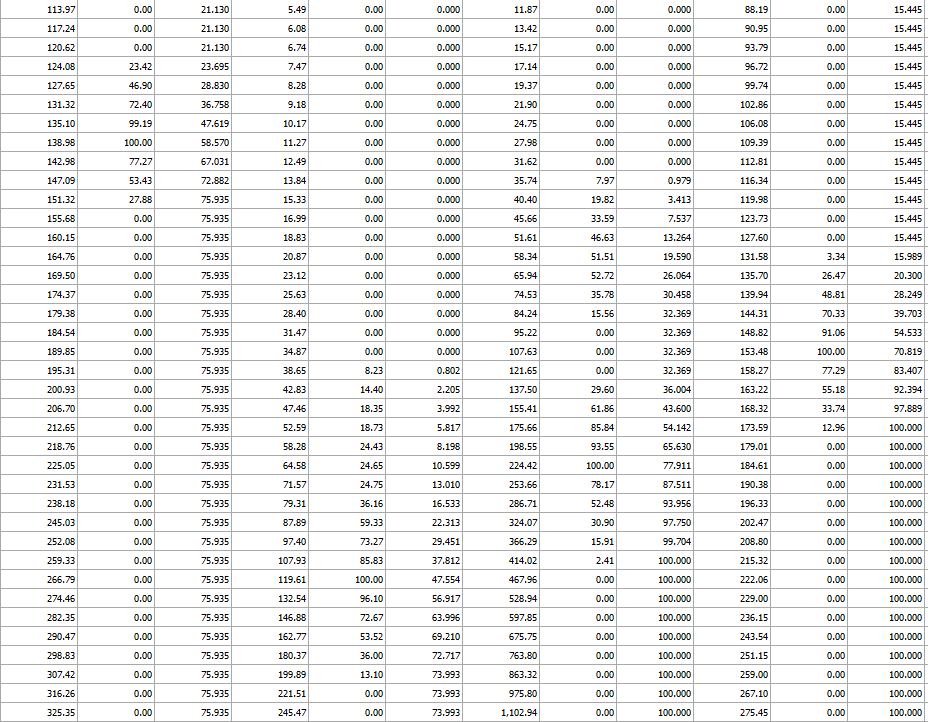


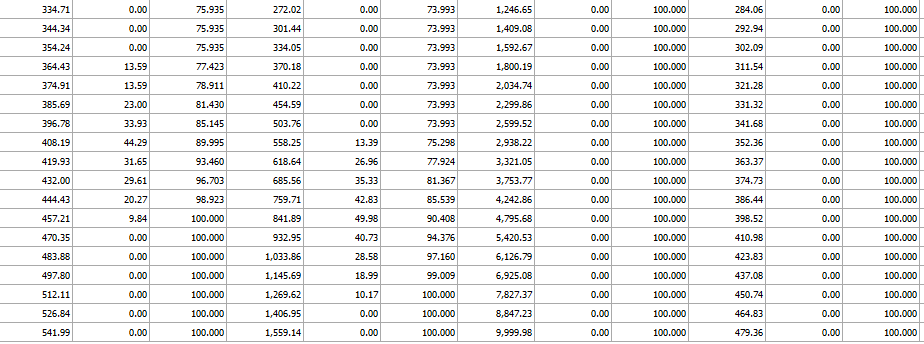


**Fig2 a**


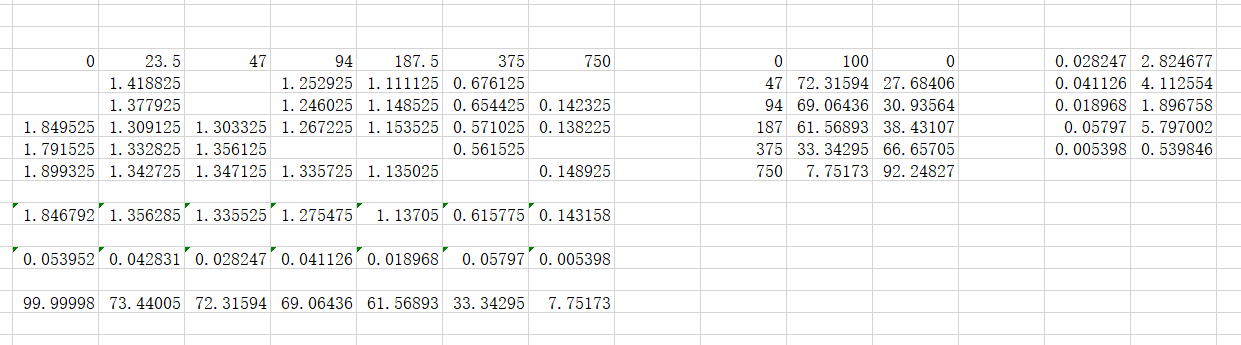


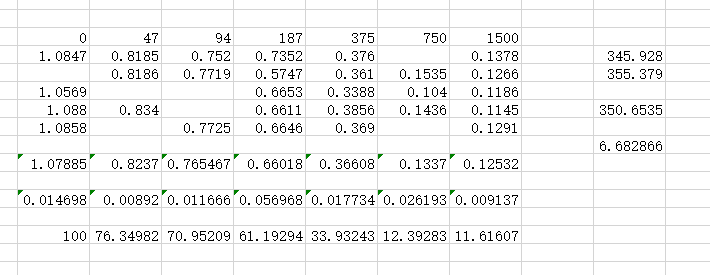


**Fig2 b**


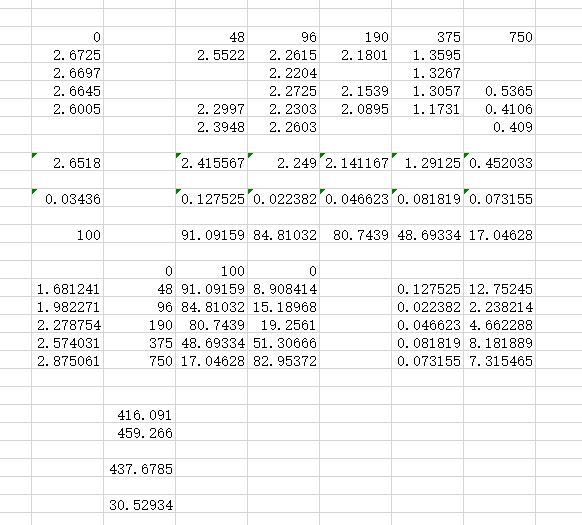


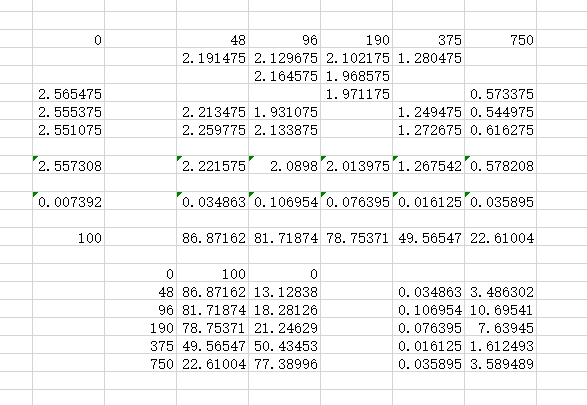


**Fig2 c**


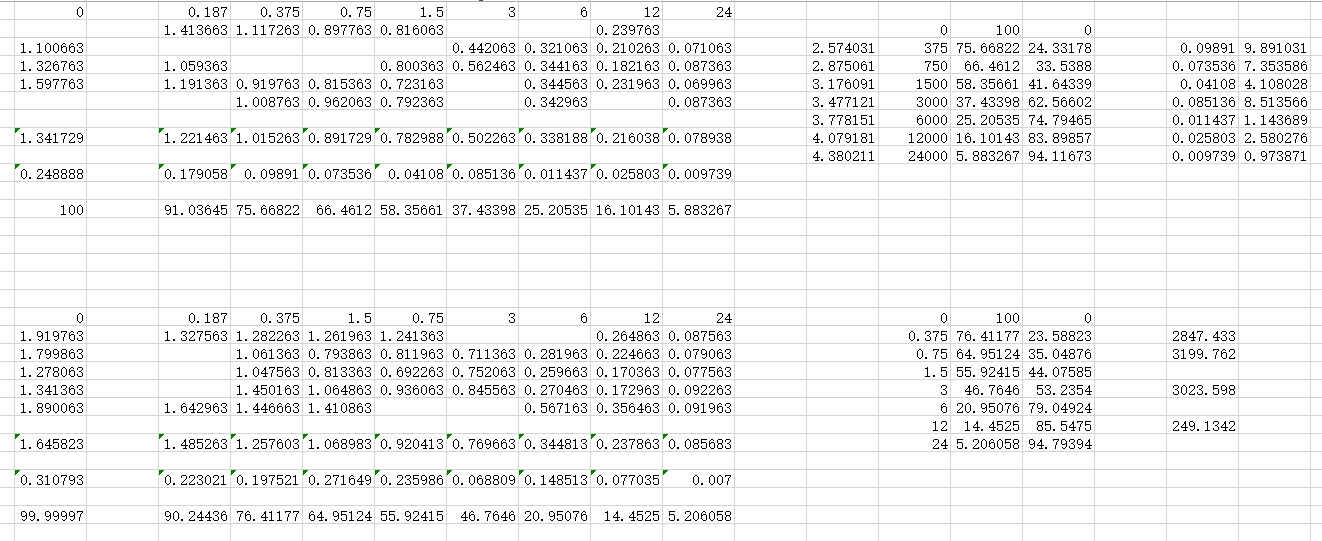


**Fig2 d**


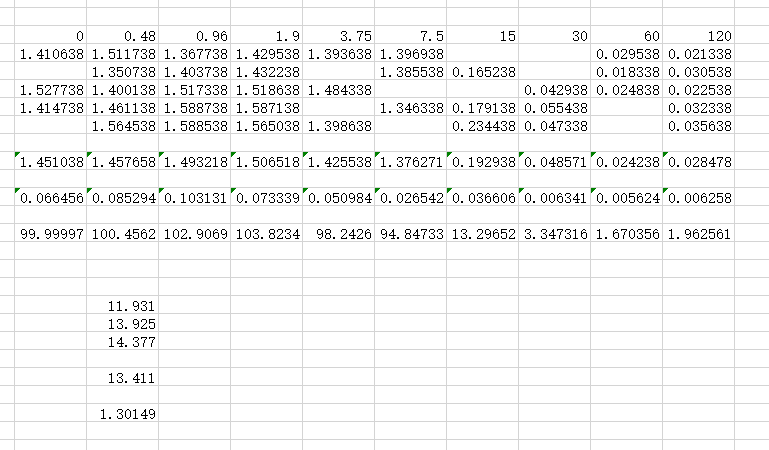


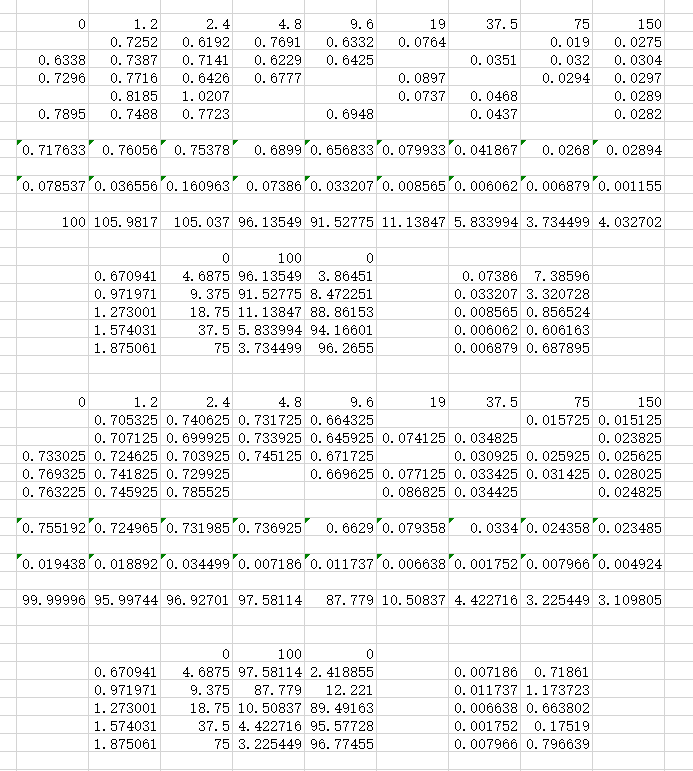


**Fig2 e**


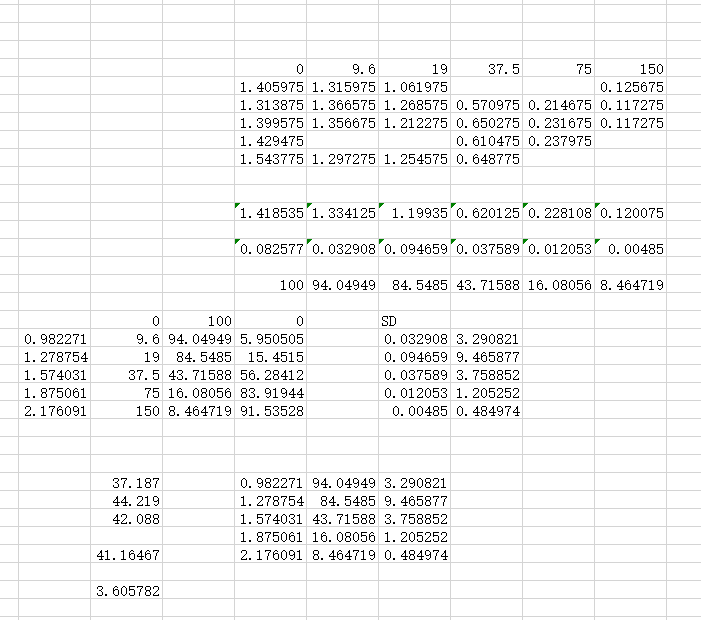


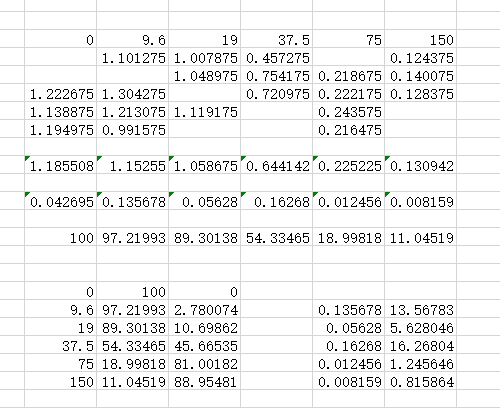


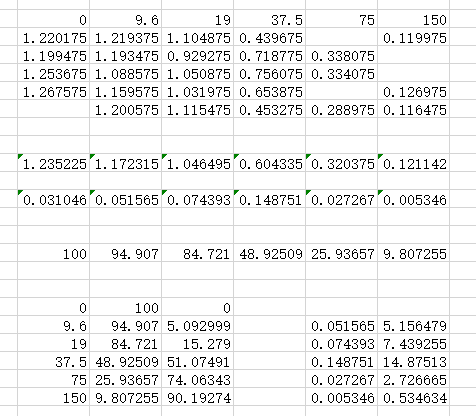


**Fig2 f**


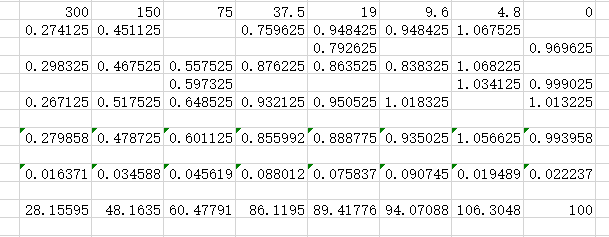


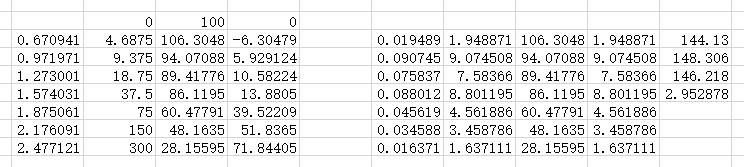


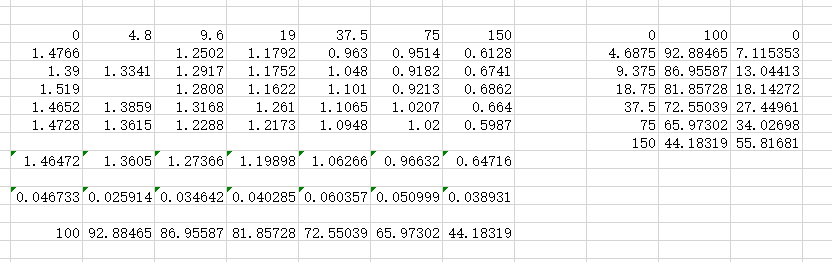


**Fig3 a-PBS**


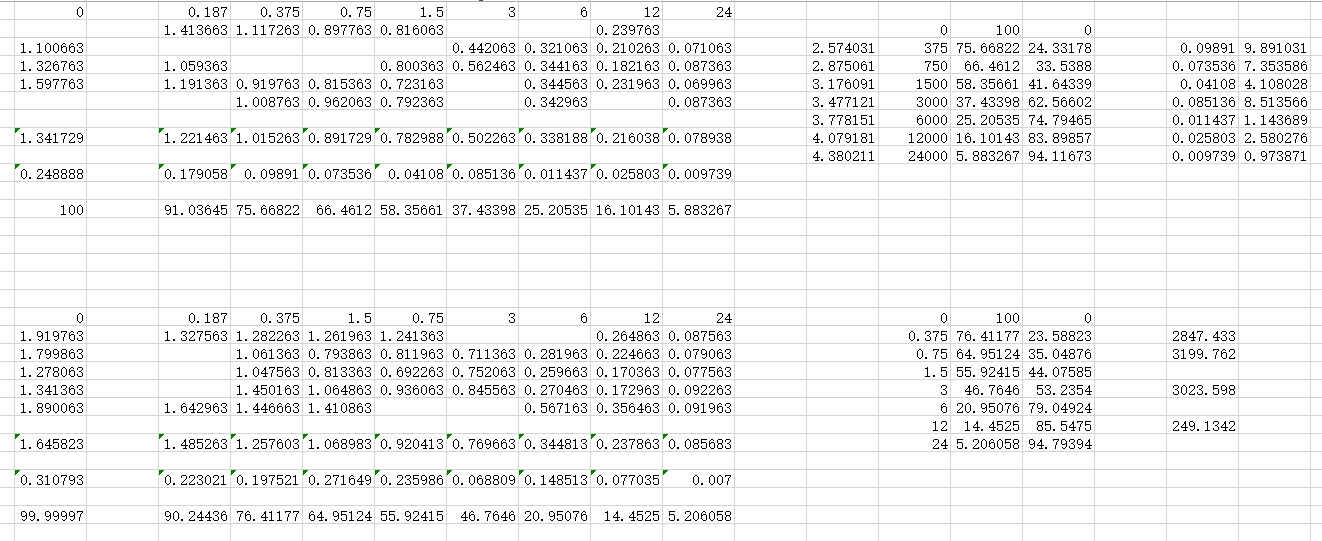


**Fig3 a- 7901/exo**


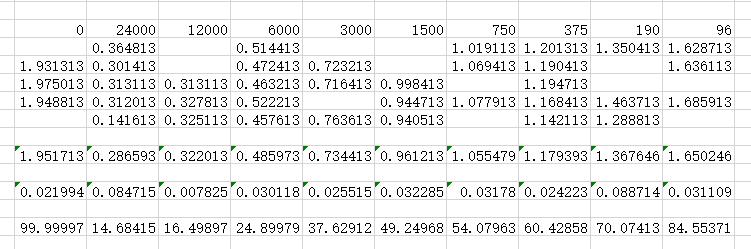


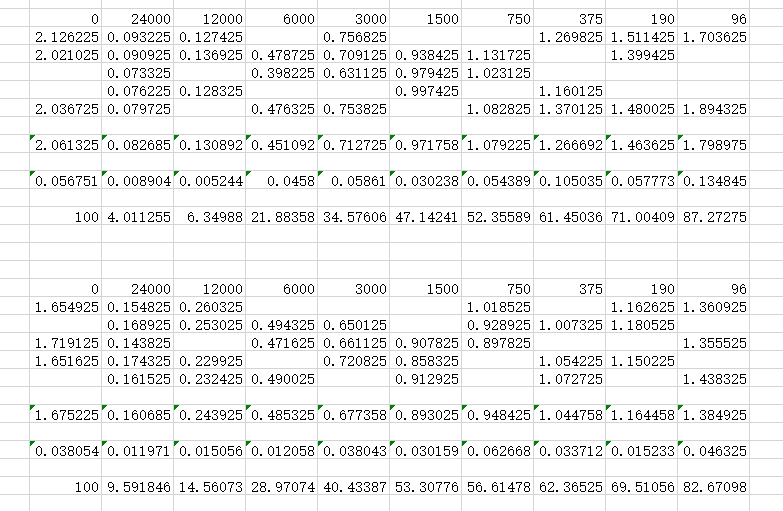


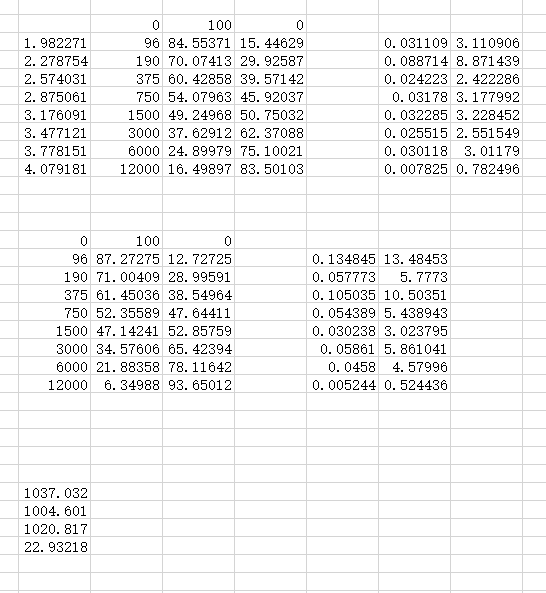


**Fig3 a-803/exo**


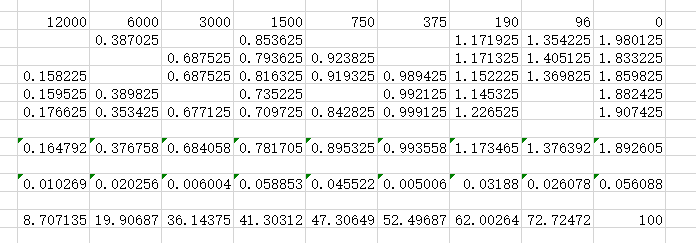


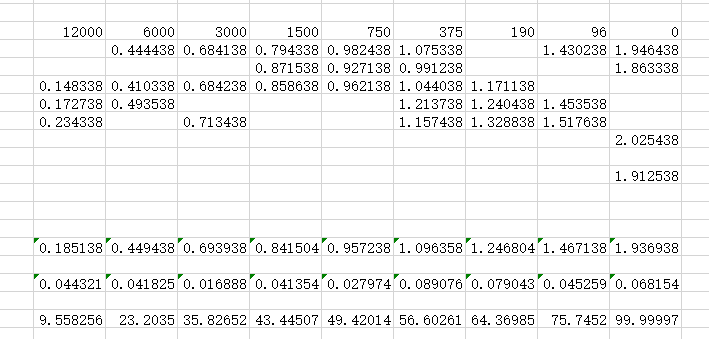


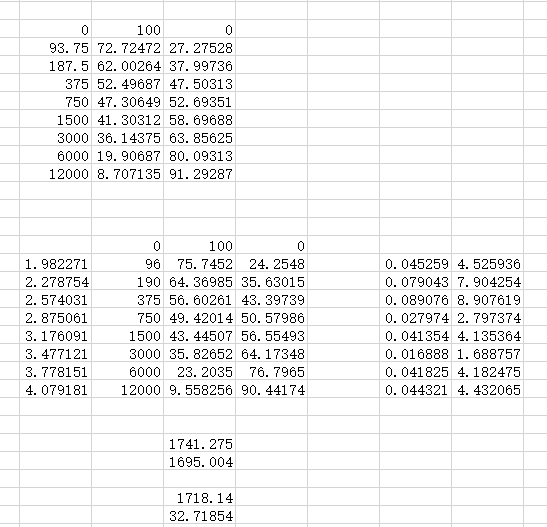


**Fig3b-PBS**


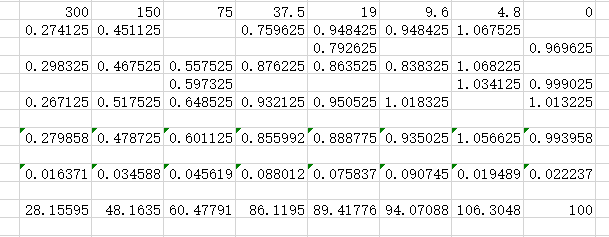


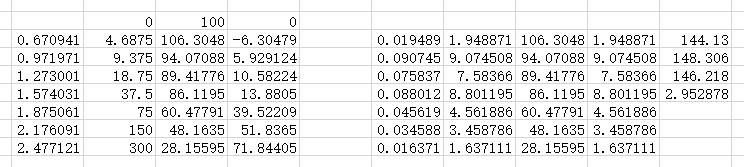


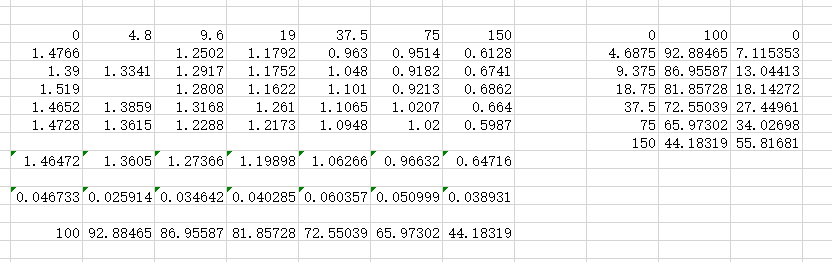


**Fig3 b- 7901/exo**


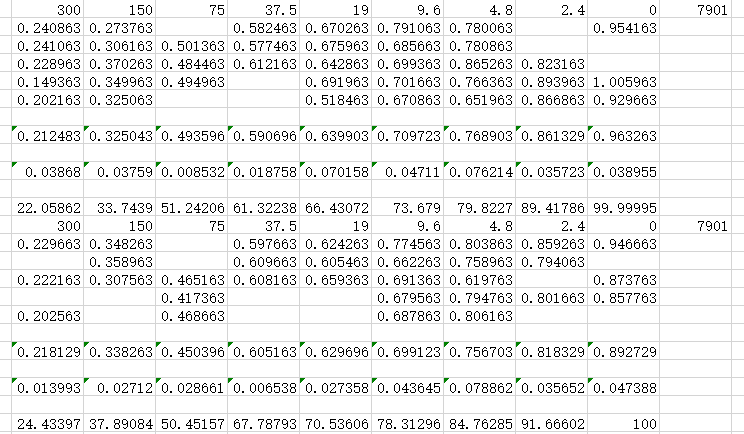


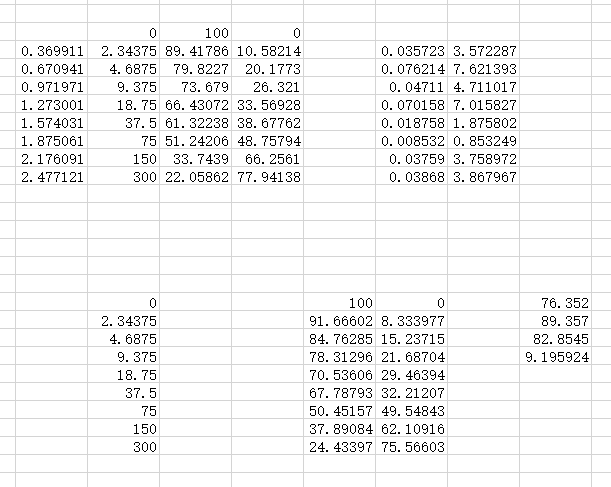


**Fig3 b- 803/exo**


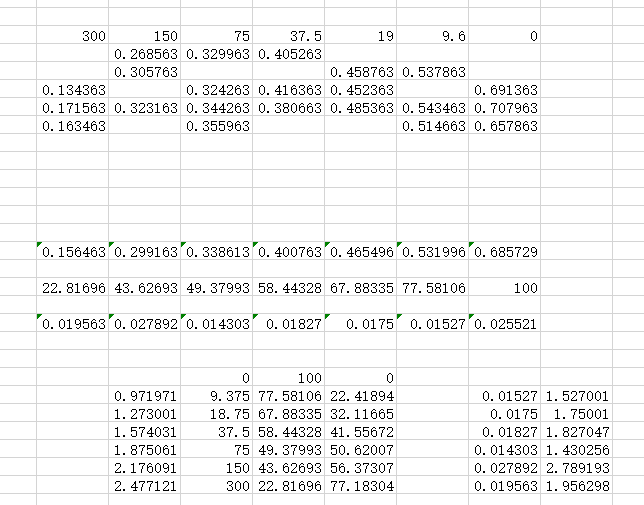


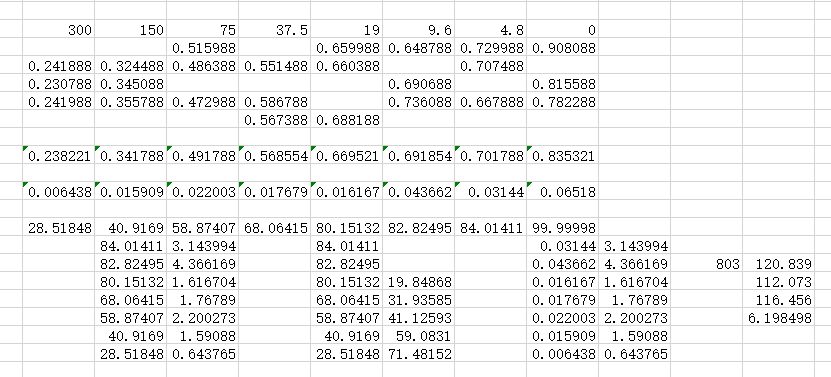


**Fig5 a**


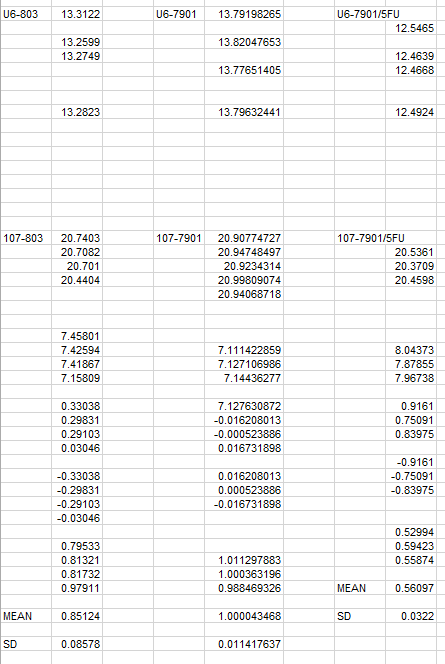


**Fig5 b**


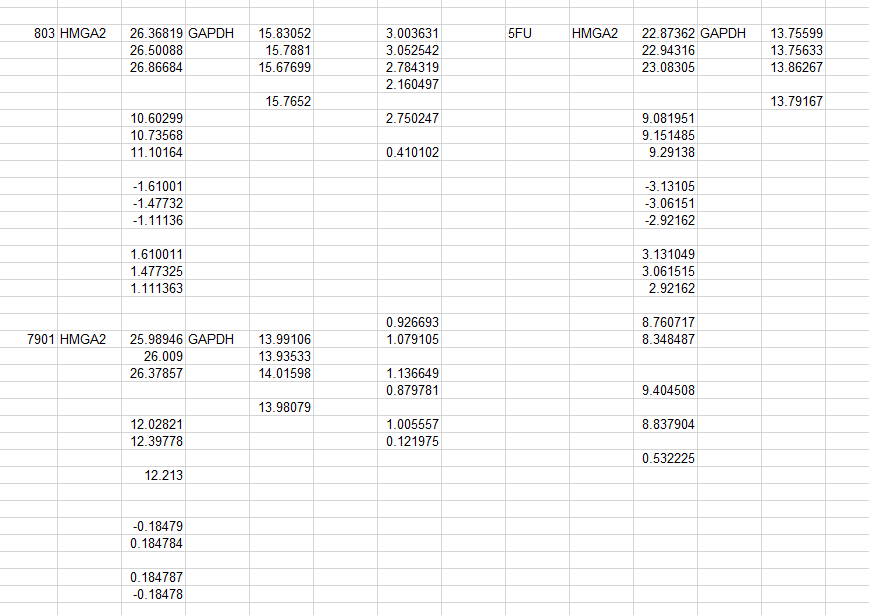


**Fig5 c**


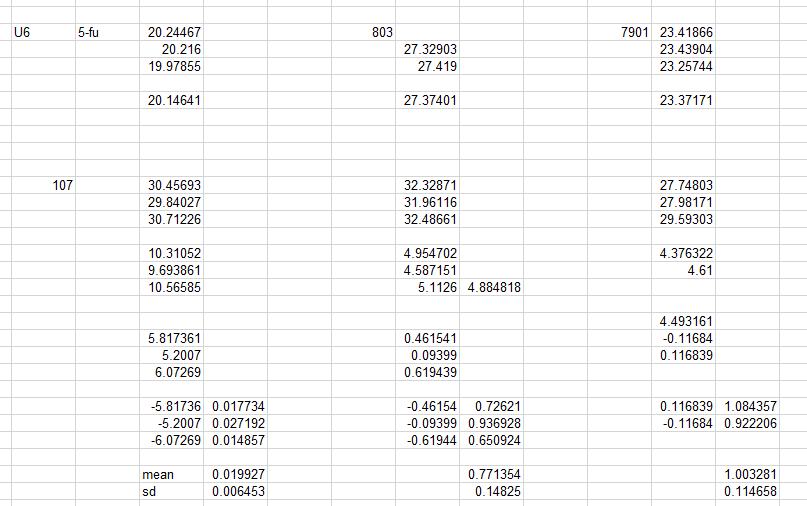


**Fig6 b**


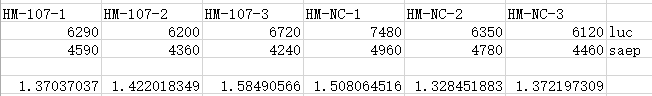


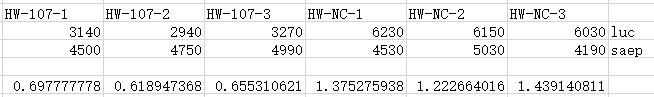


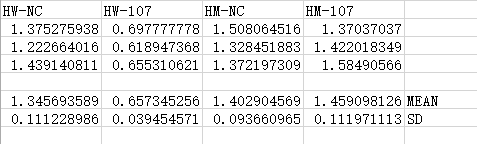


**Fig7 a-DMSO/Exo**


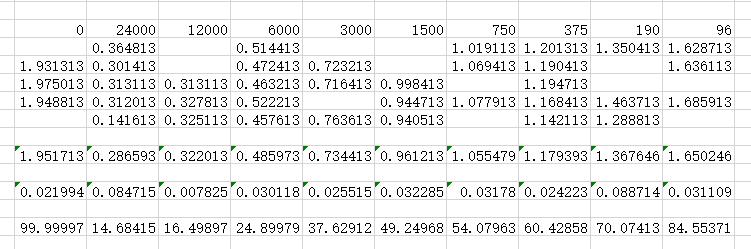


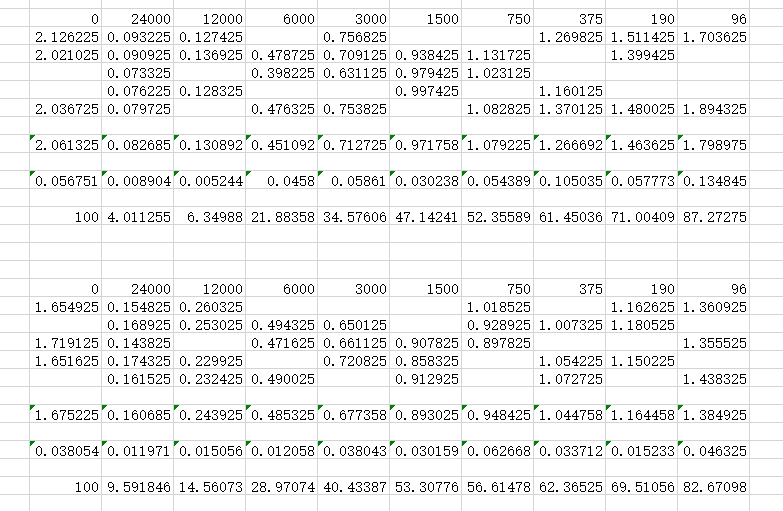


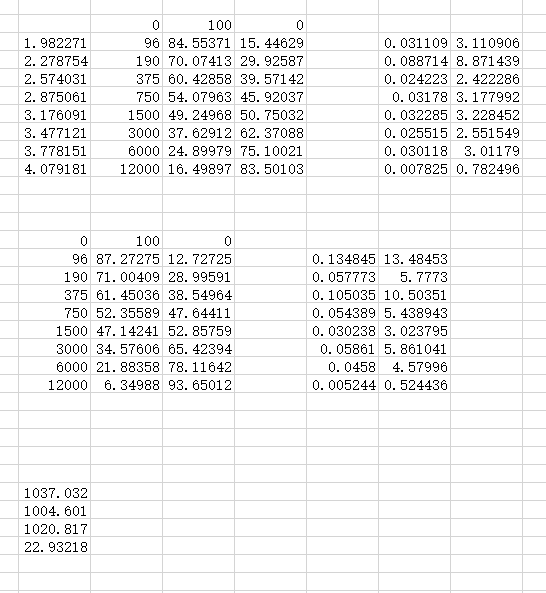


**Fig7 a-GW4849/Exo**


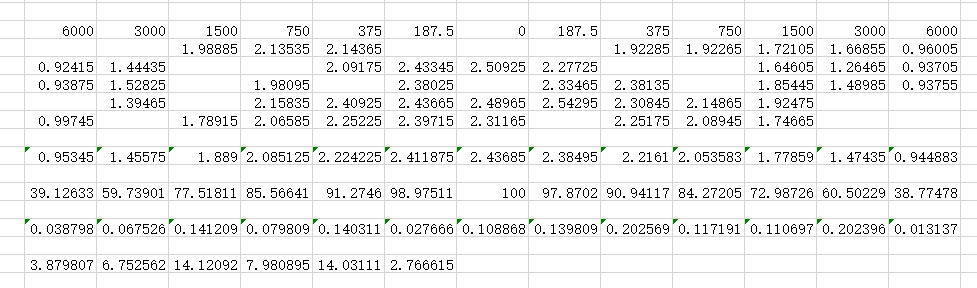


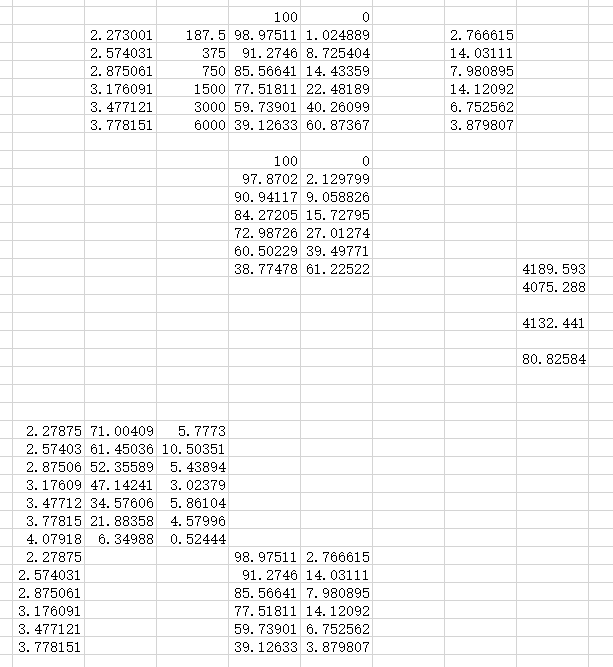


**Fig7b-DMSO/exo**


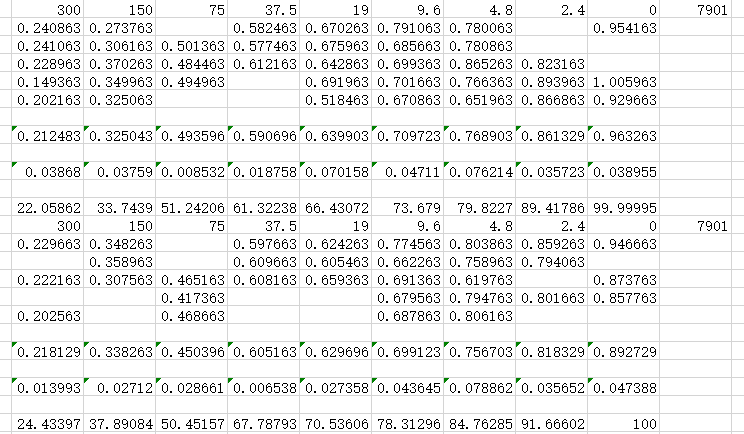


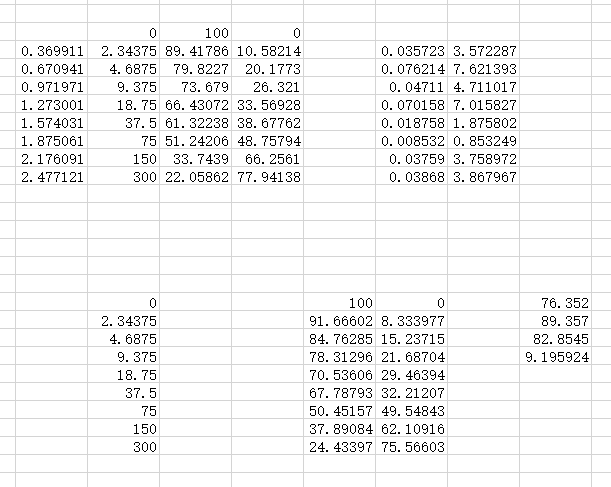


**Fig7b-GW4869/exo**


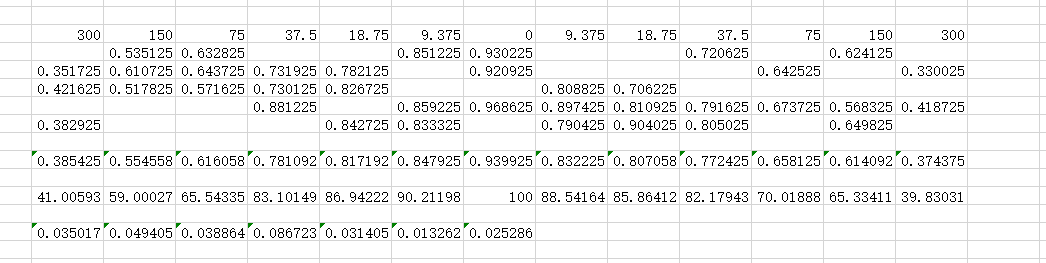


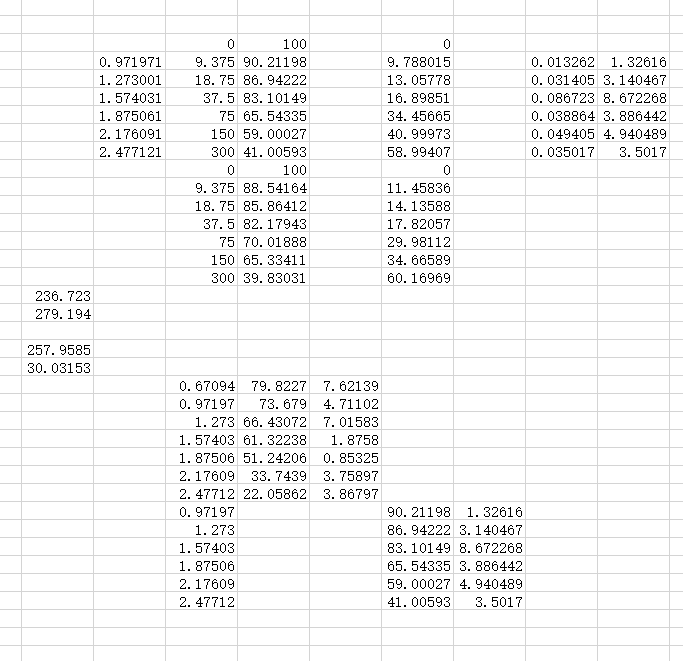


**Fig8 a**


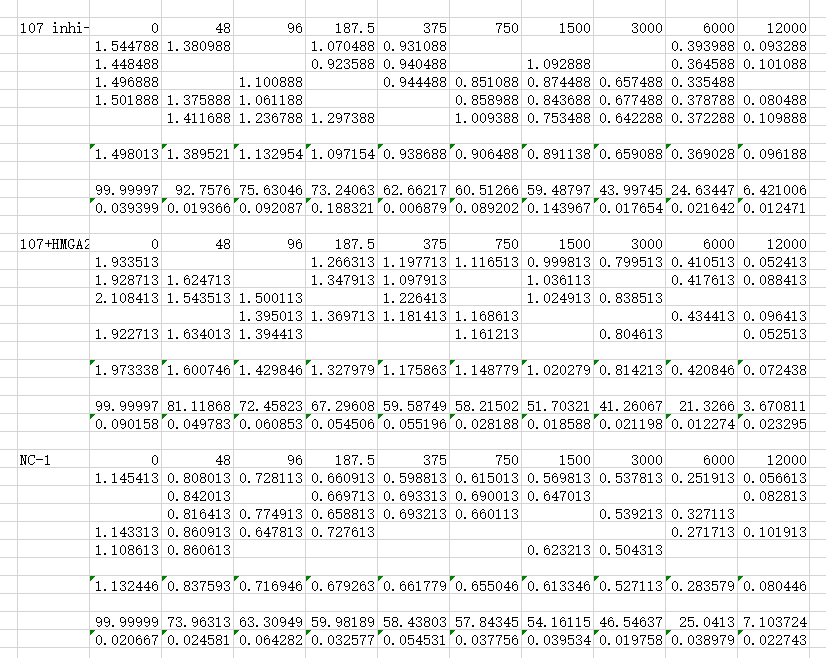


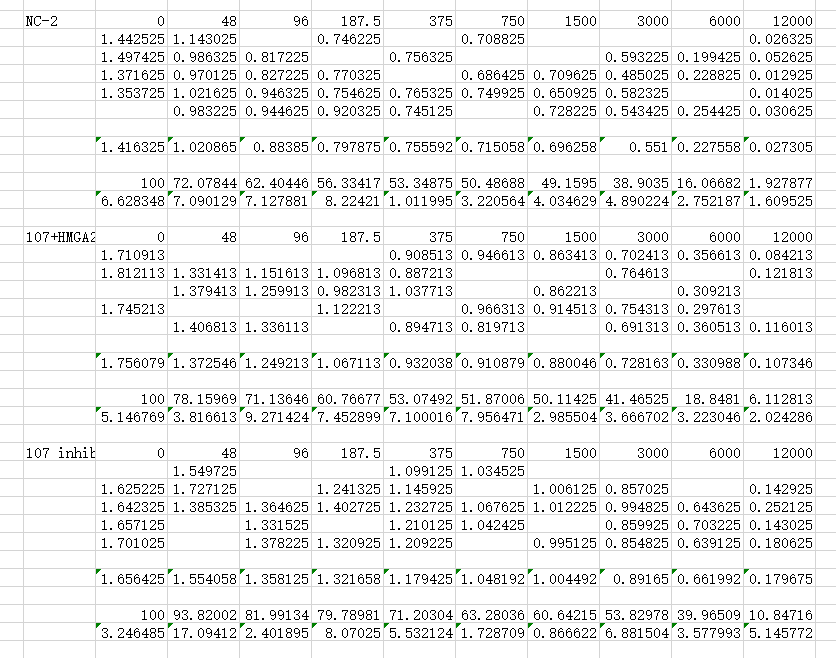


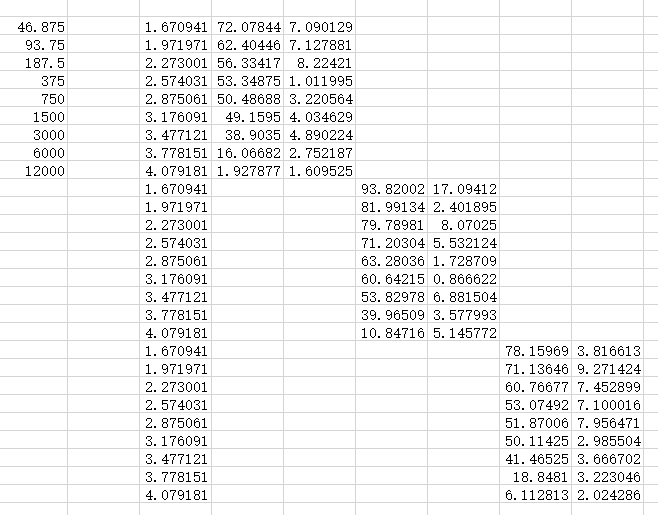


**Fig8 b**


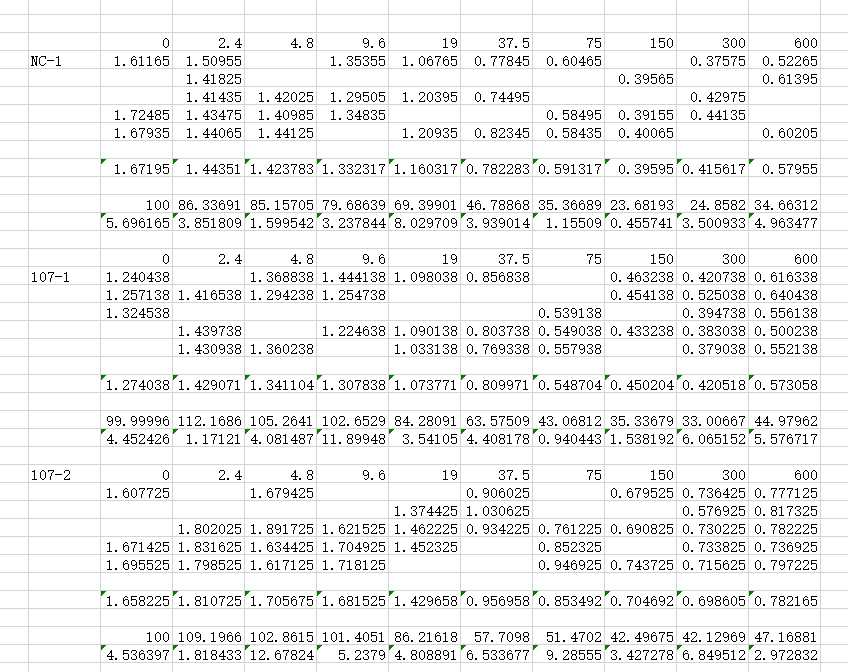


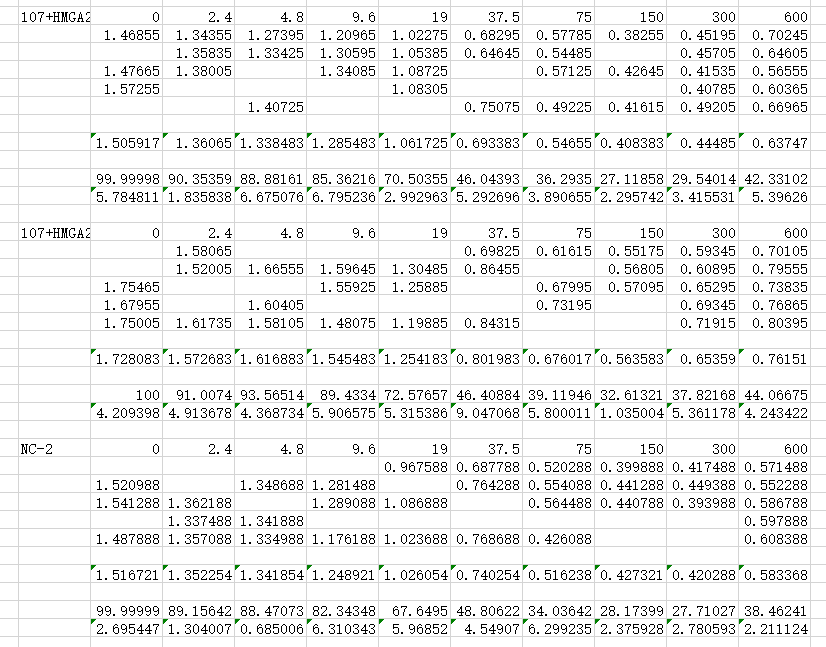


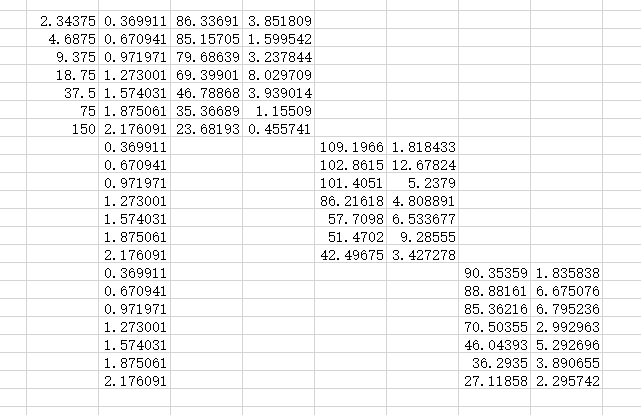


**Fig9 d-f**


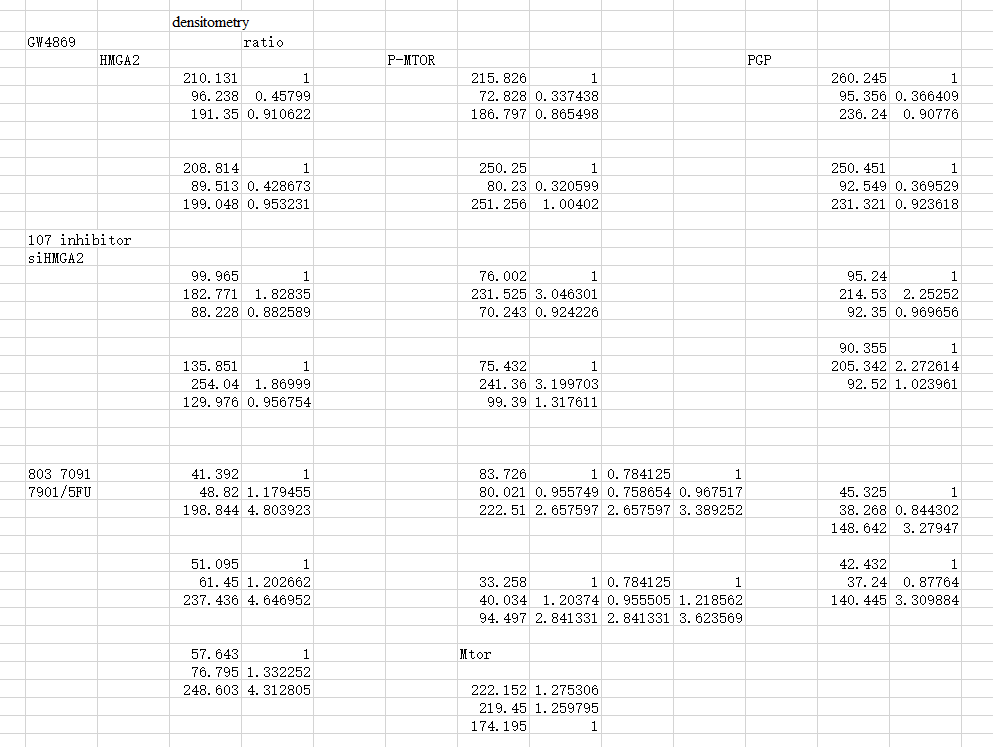


**Fig 10 a and b**


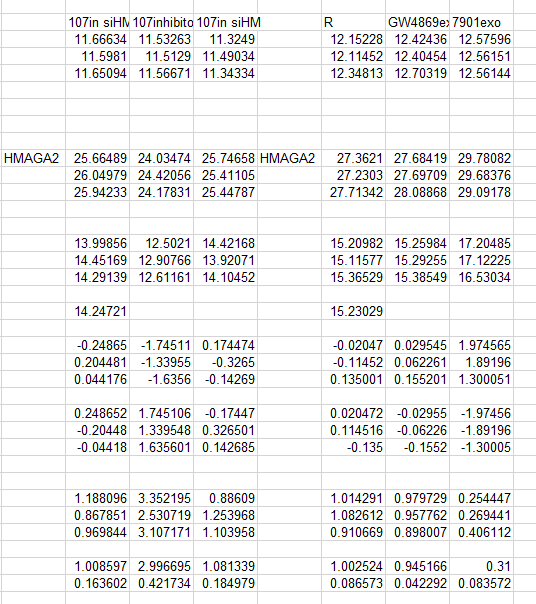


**Fig 11 a-d**

**Fig 11 a**


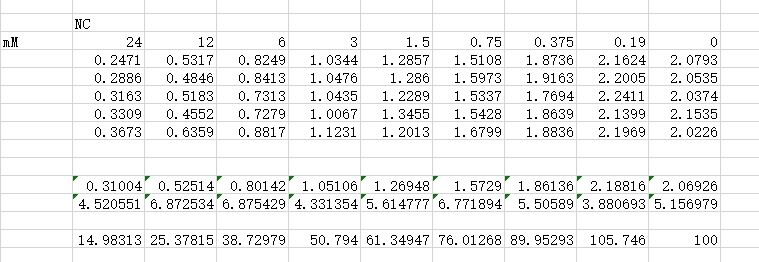


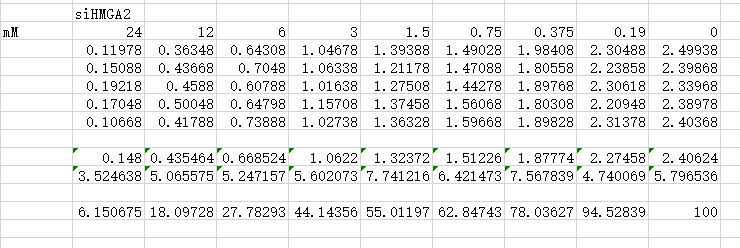


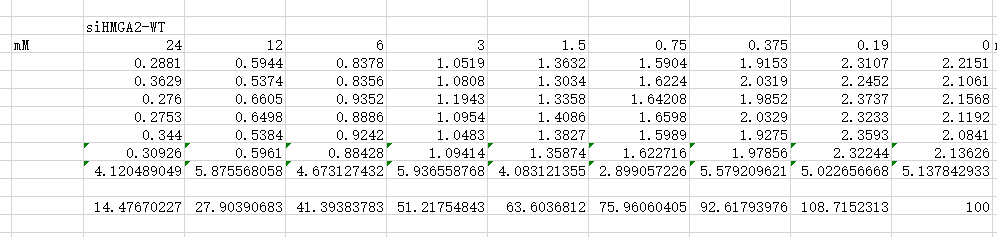


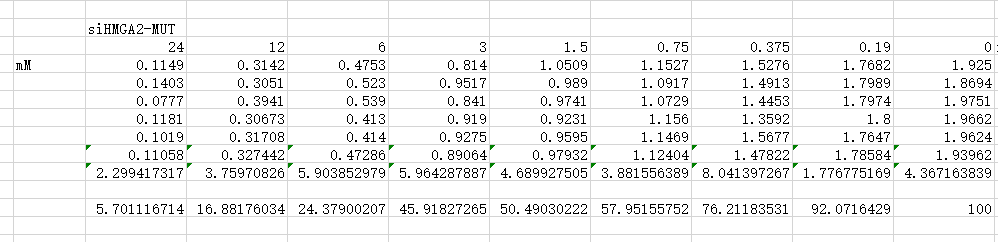


**Fig 11 b**


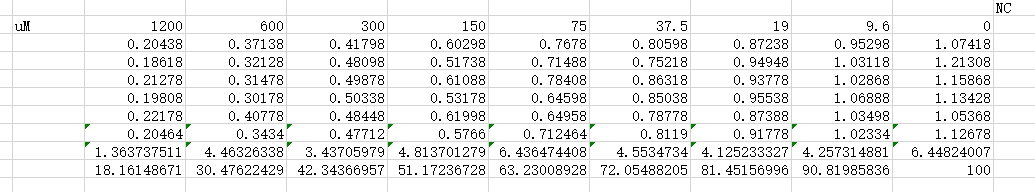


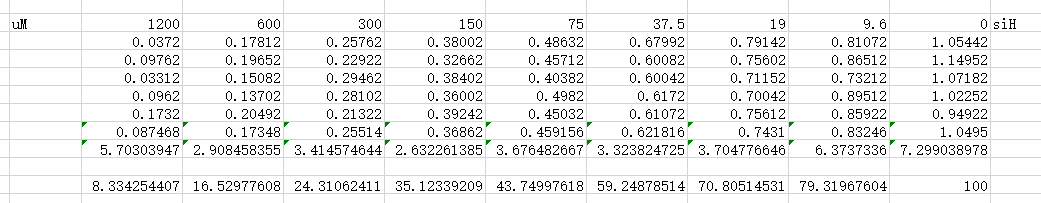


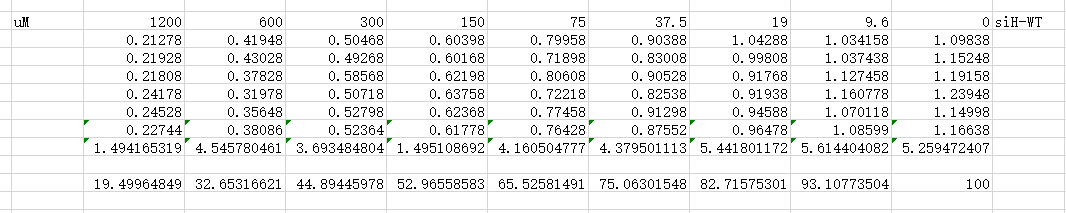


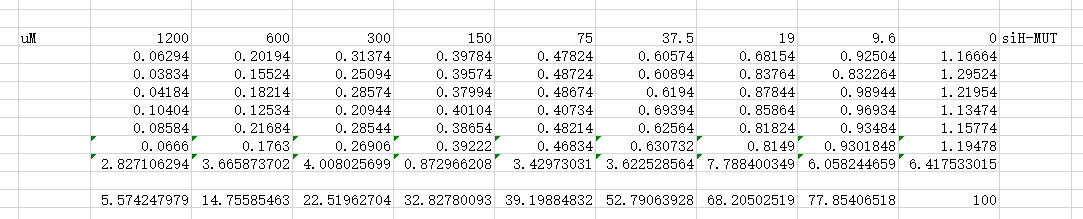


**Fig 11 c**


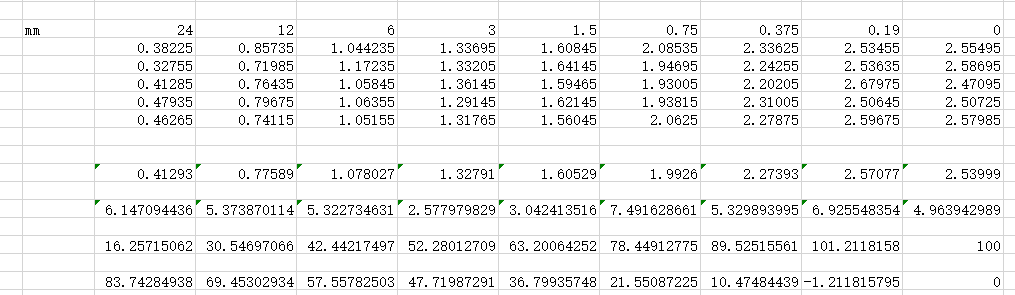


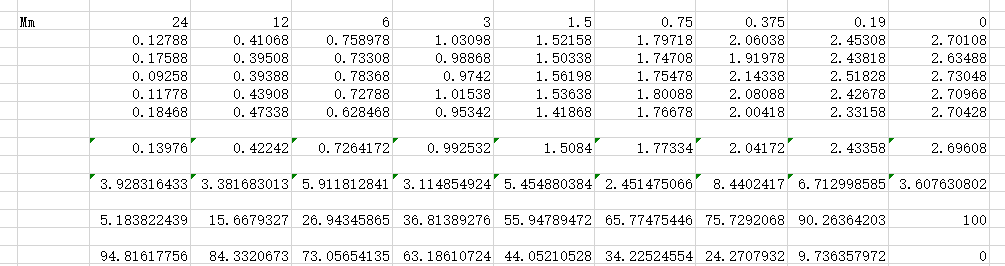


**Fig 11 d**


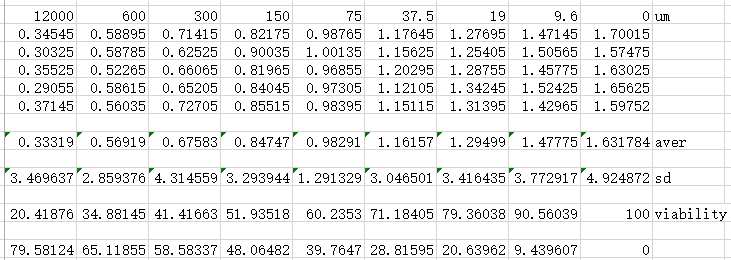


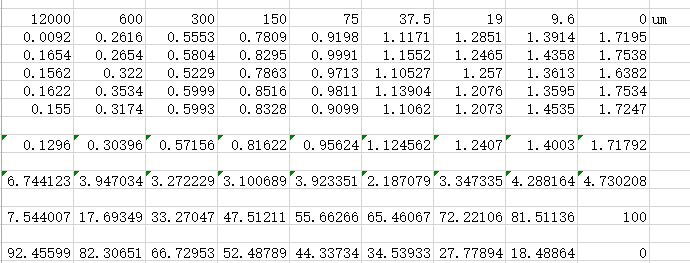


**Additional file 1: figure S1**


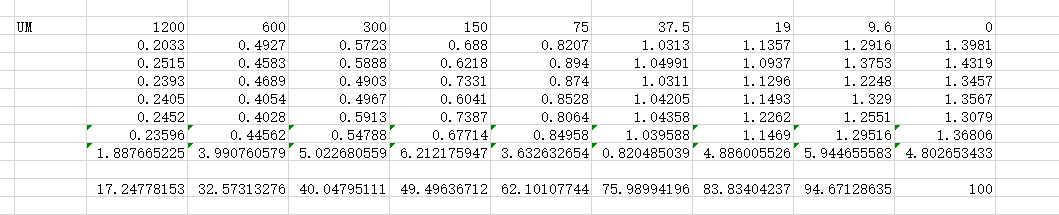


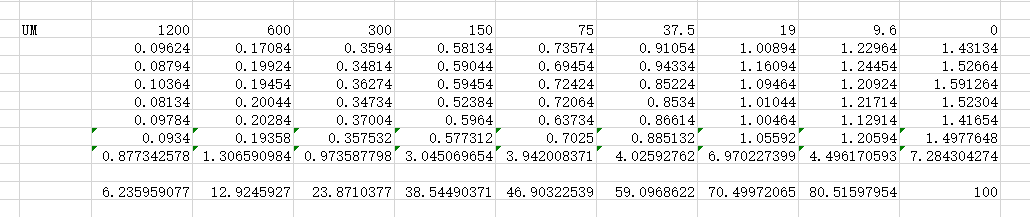


**Additional file 1: figure S2 a-d**

**S2 a**


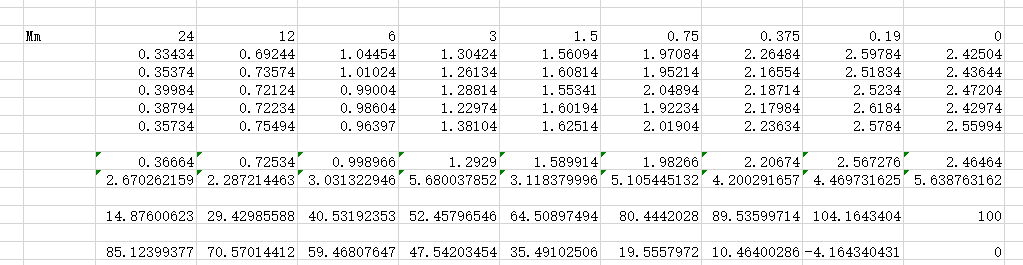


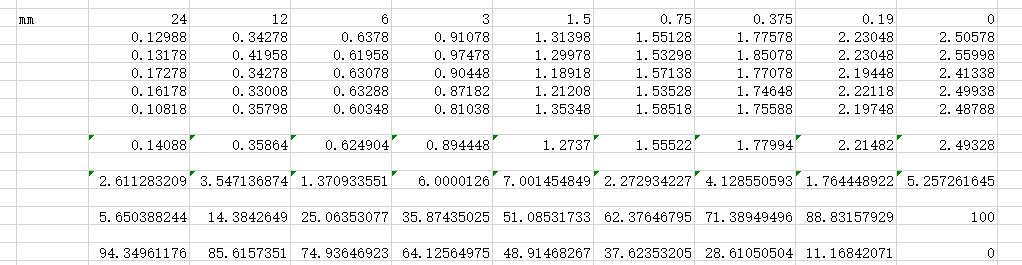


**S2 b**


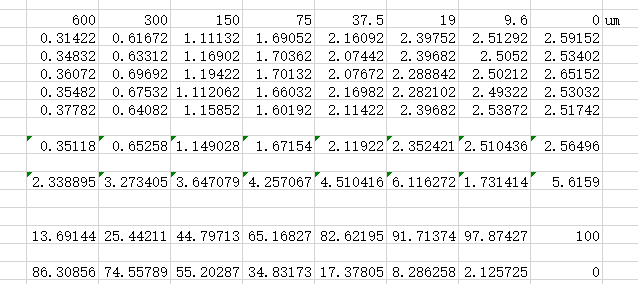


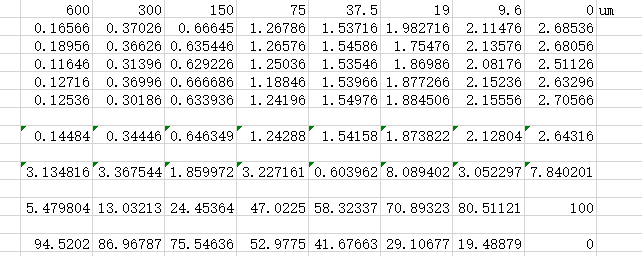


**S2 c**


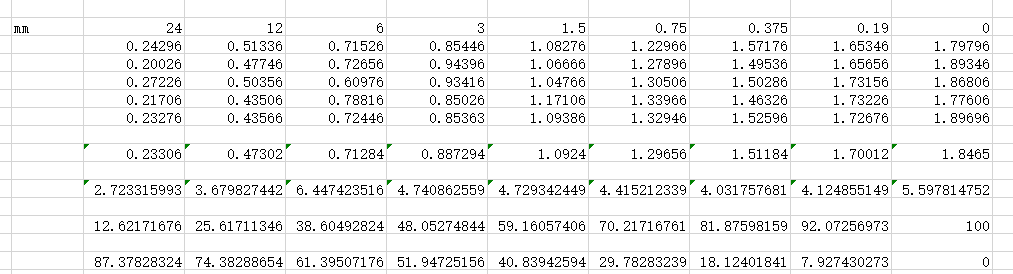


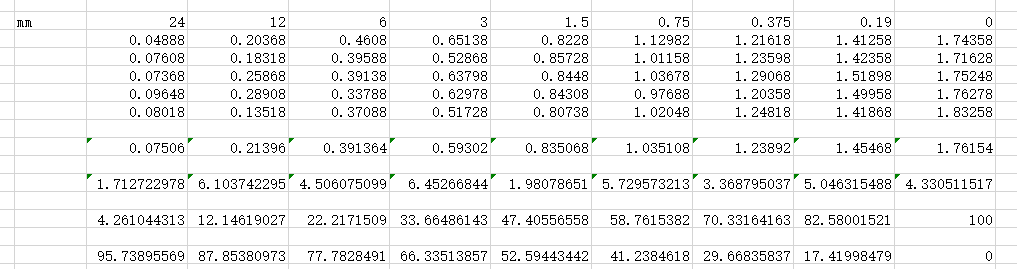


**S2 d**


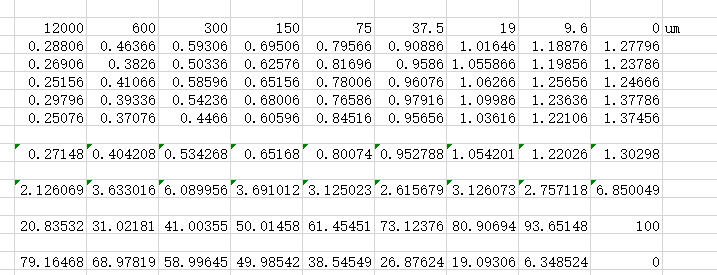


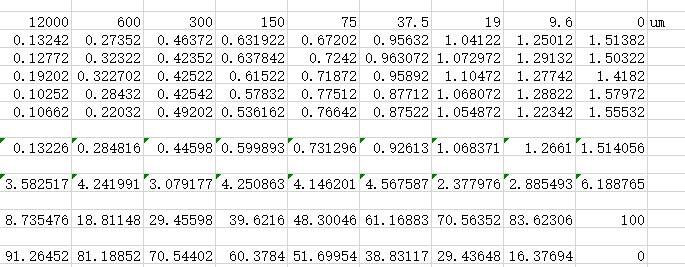


**Additional file 1: figure S3 a-c**


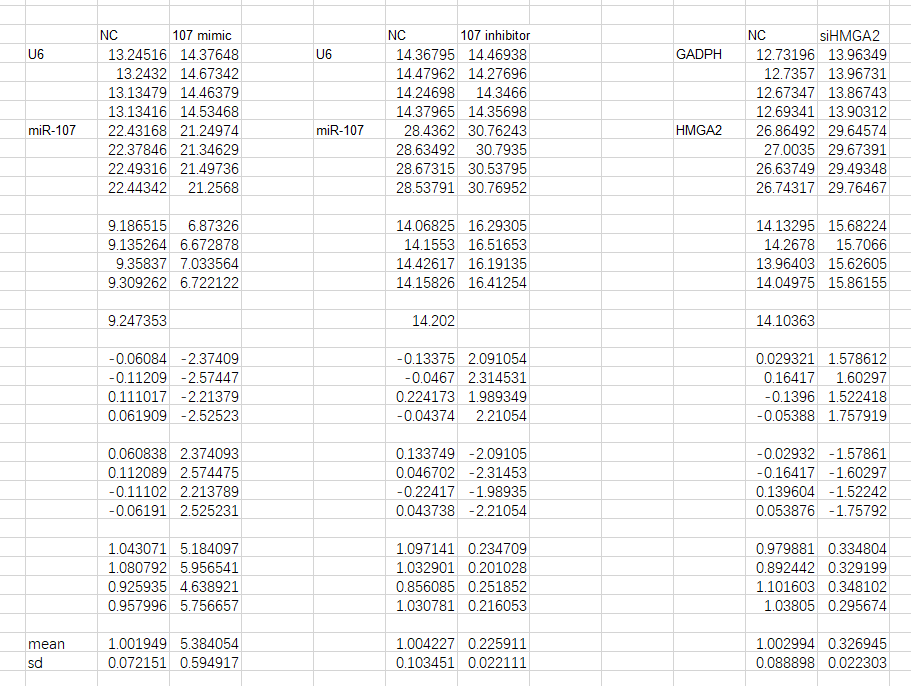


**Additional file 1: figure S4 b**


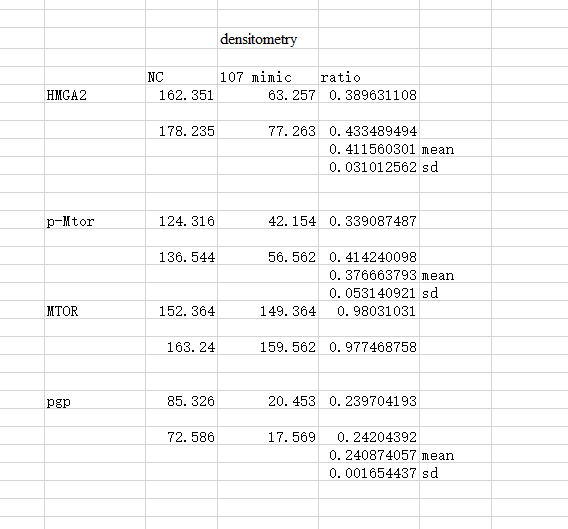


**Additional file 1: figure S5**


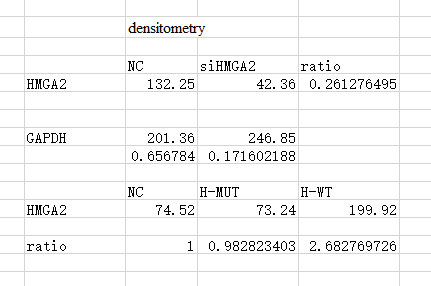

Supplement: Supplementary file 2 — Additional file 2. [file 12885_2021_9020_MOESM2_ESM.doc]
