# Supplementary material for: Exosomal microRNA-107 reverses chemotherapeutic drug resistance of gastric cancer cells through HMGA2/mTOR/P-gp pathway
Source: BMC Cancer. 2021 Dec 2;21:1290. doi: 10.1186/s12885-021-09020-y (PMC8638432; doi:10.1186/s12885-021-09020-y)
Supplement: Supplementary file 3 — Additional file 3. [file 12885_2021_9020_MOESM3_ESM.doc]

**Western blotting**

**Fig 1 c**


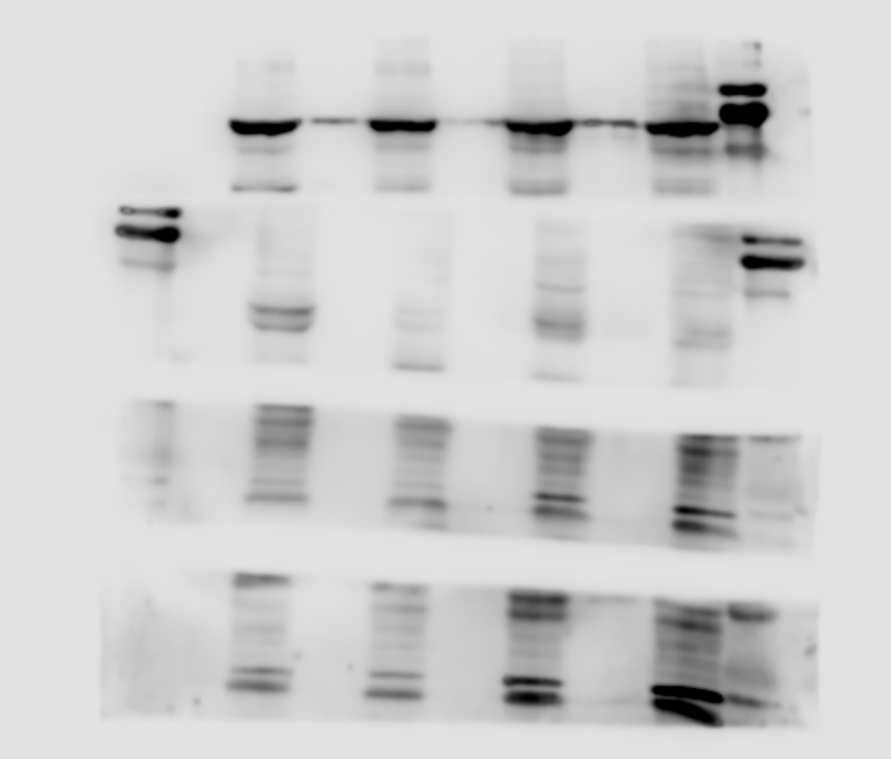


HSP70


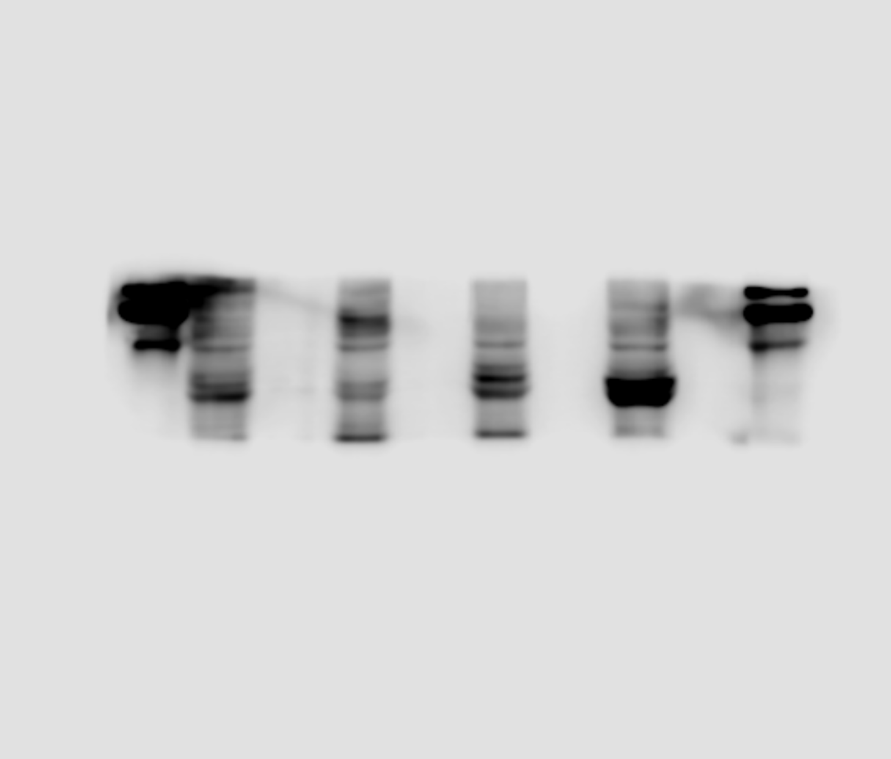


Lamin B1


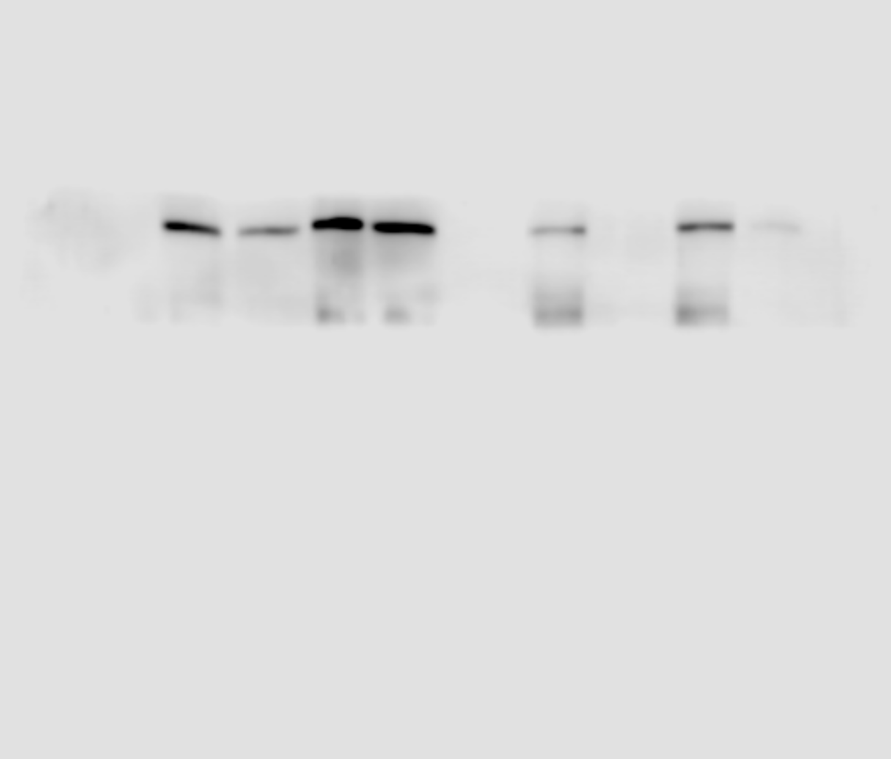


CD63

68 kDa

72 kDa

26 kDa

**Fig 9 a**


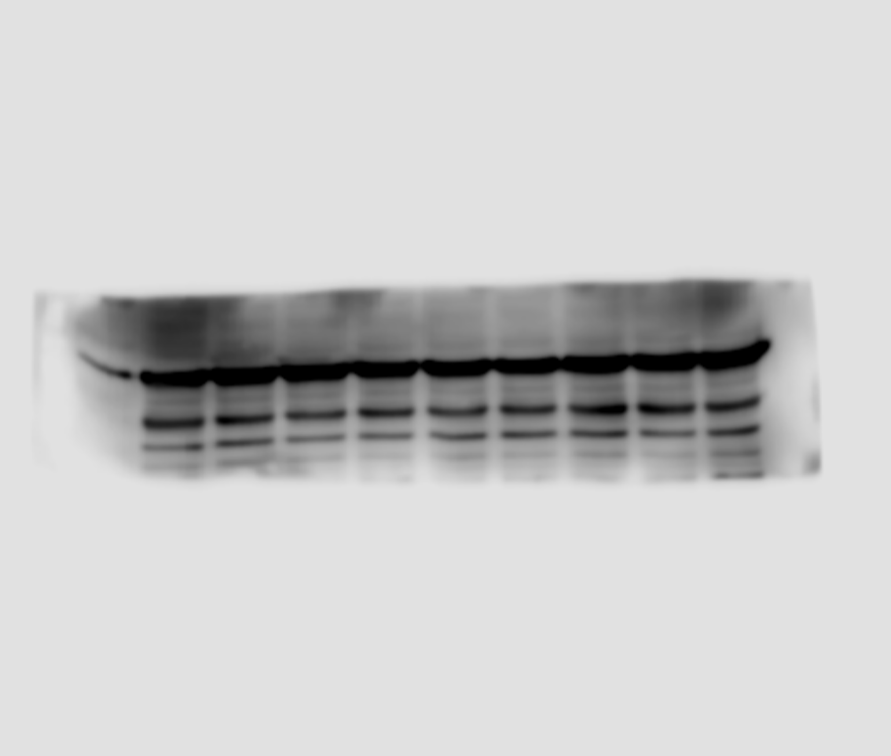


GAPDH

P-gp


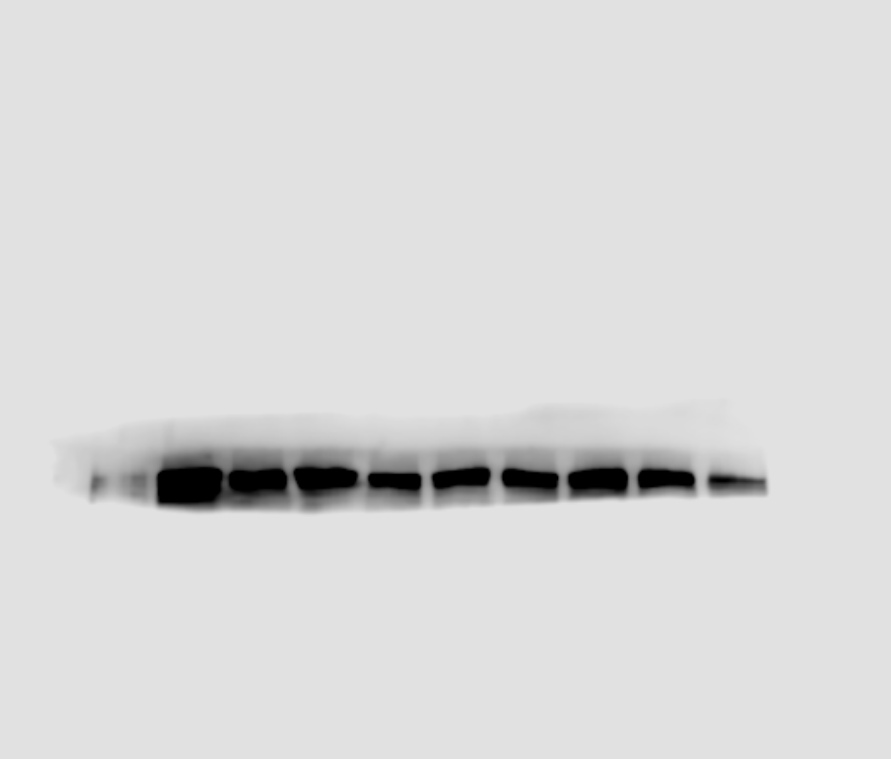


mTOR


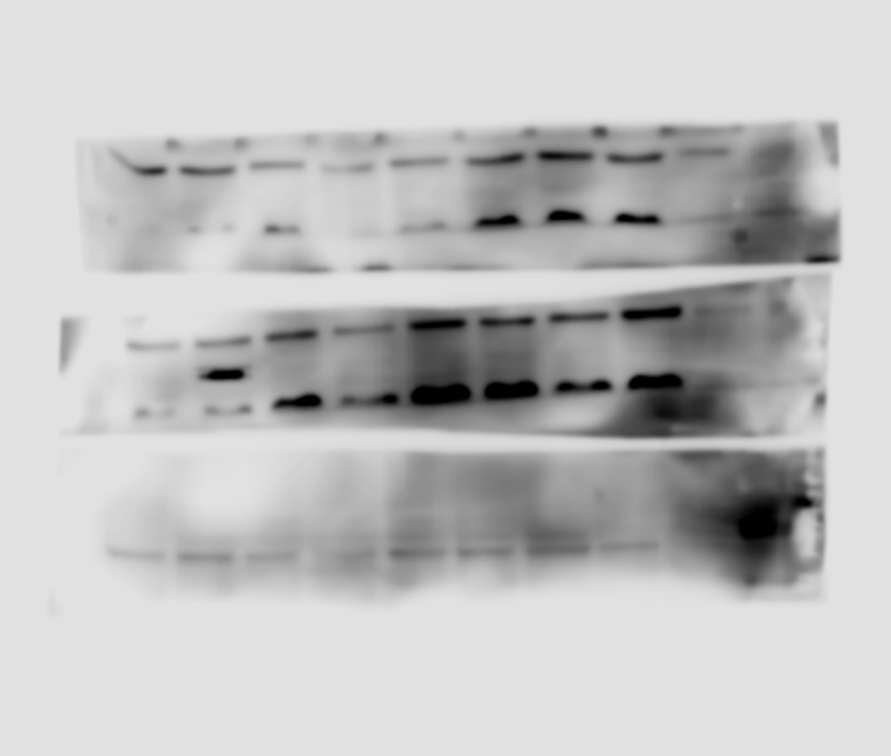


HMGA2


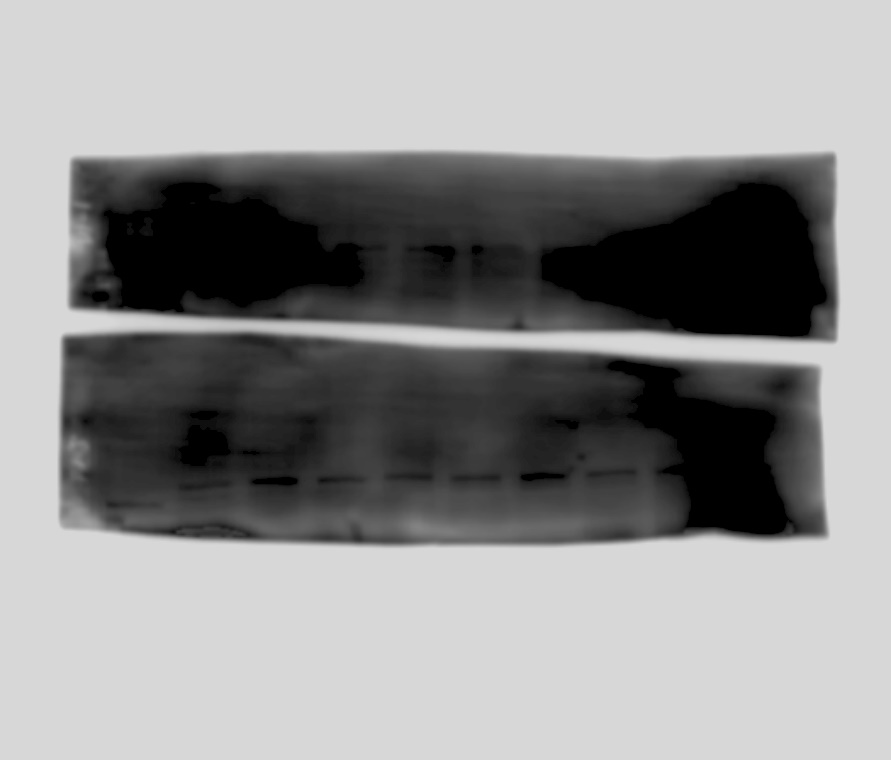


p-mTOR

12 kDa

289 kDa

289 kDa

36 kDa


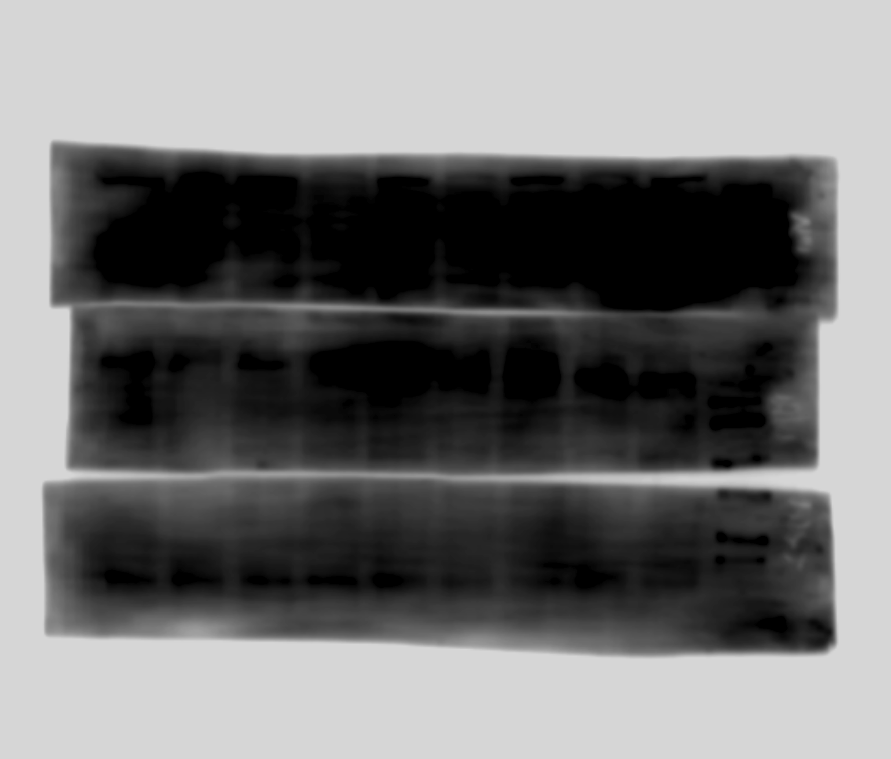


170 kDa

**Fig 9 b**


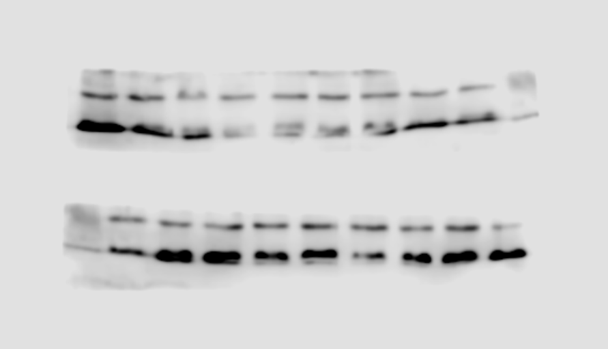


HMGA2


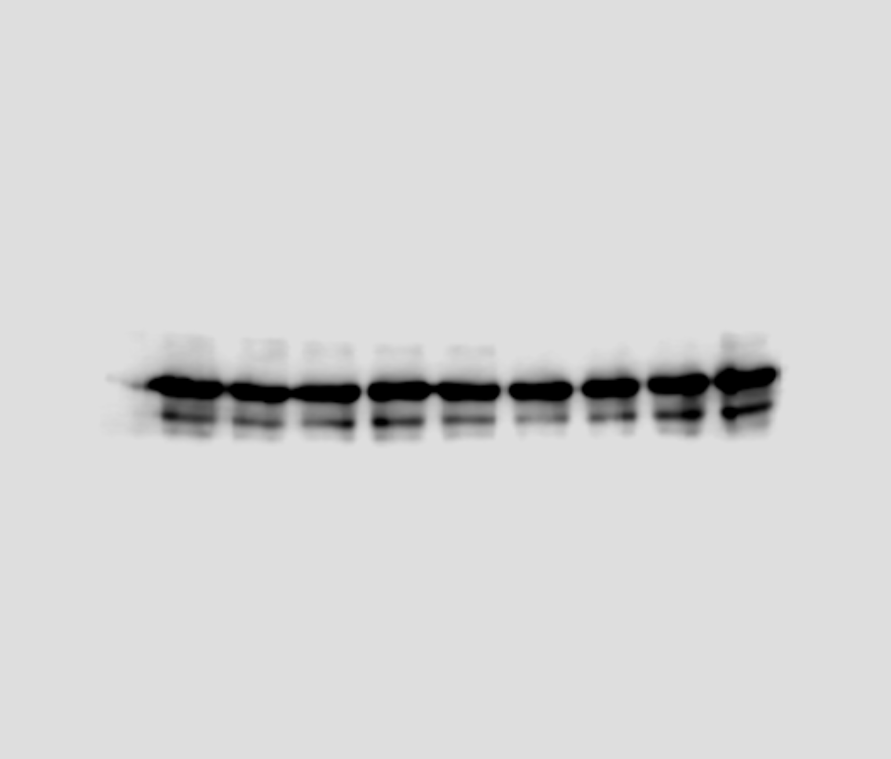


GAPDH


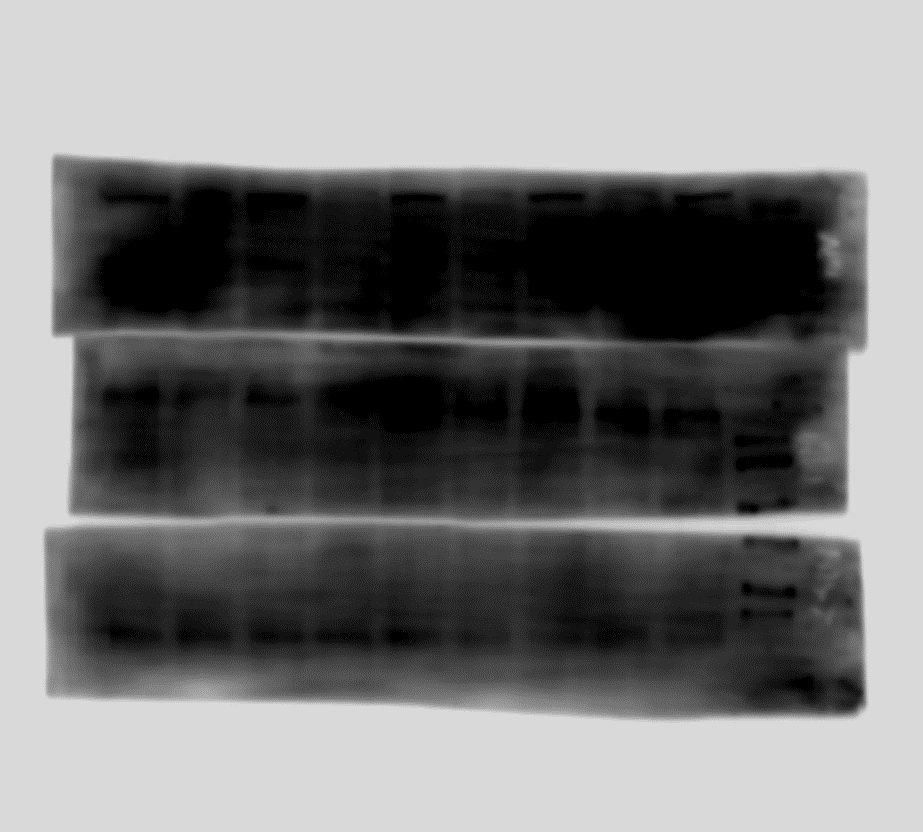


P-gp


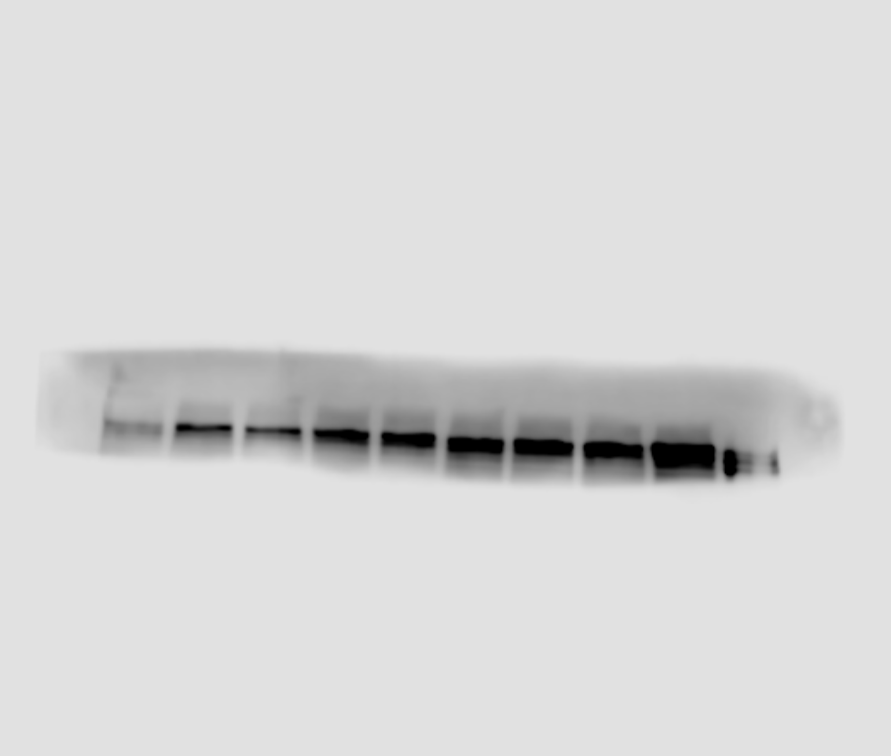


mTOR

12 kDa

289 kDa

170 kDa

36 kDa

p-mTOR


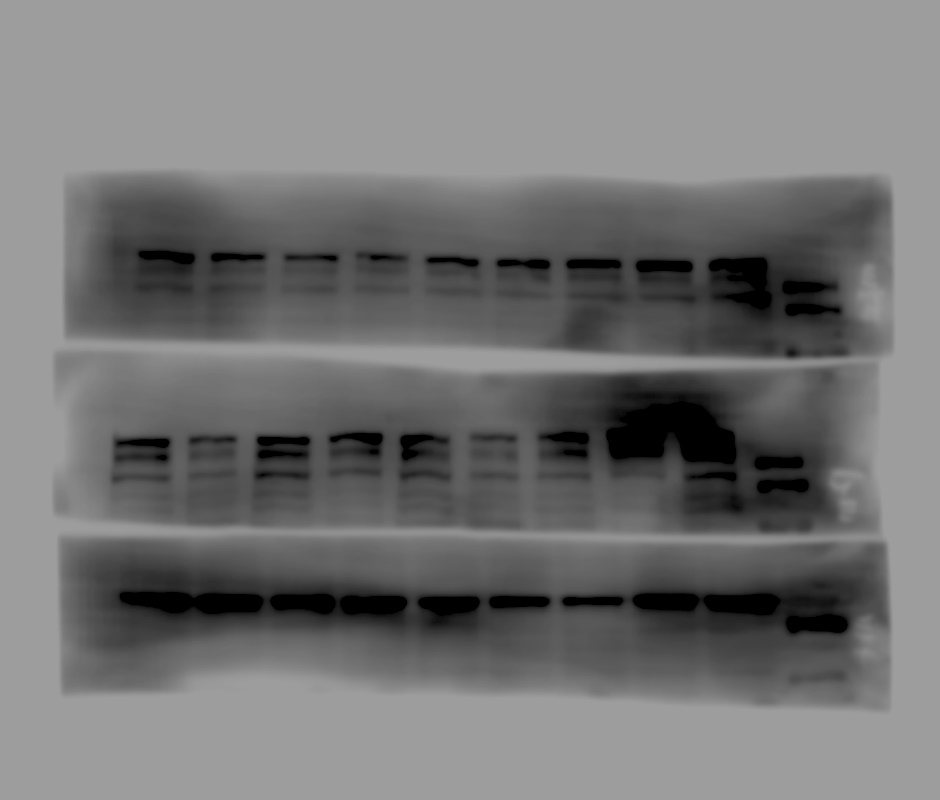


289 kDa

**Fig 9 c**

p-mTOR


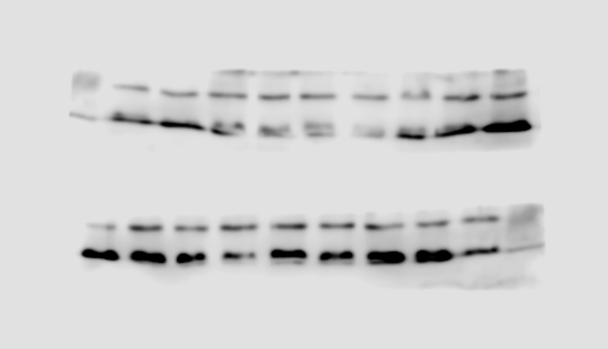


HMGA2


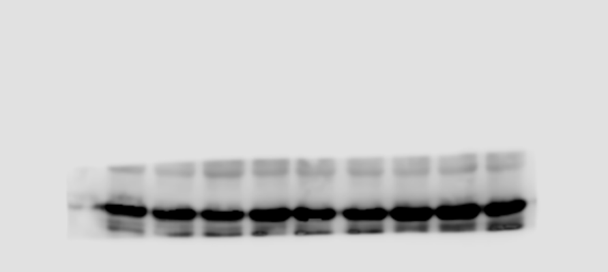


GAPDH


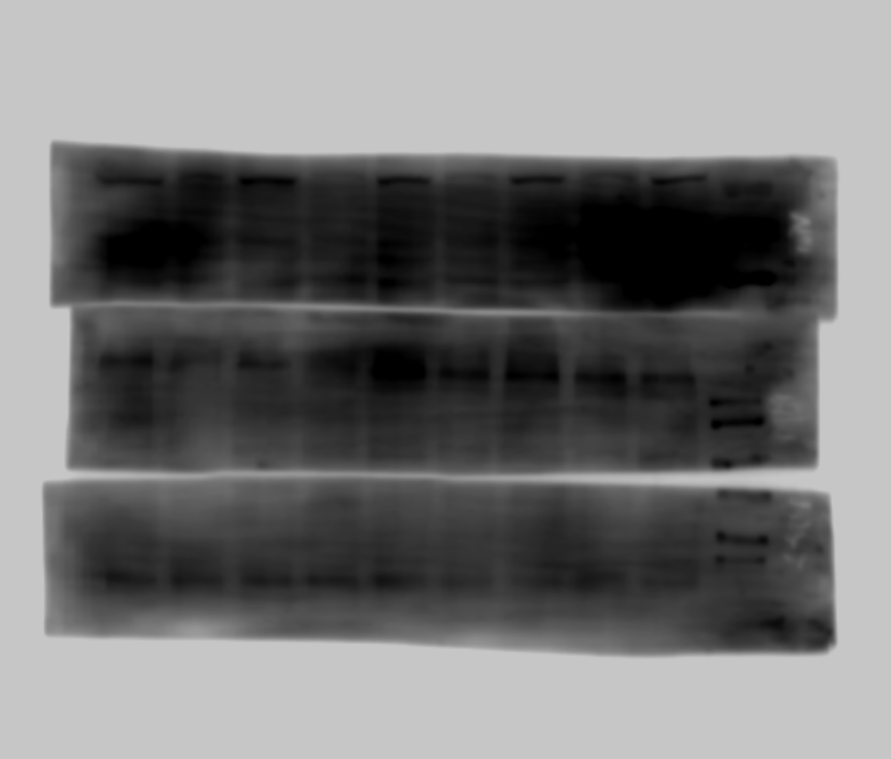


P-gp


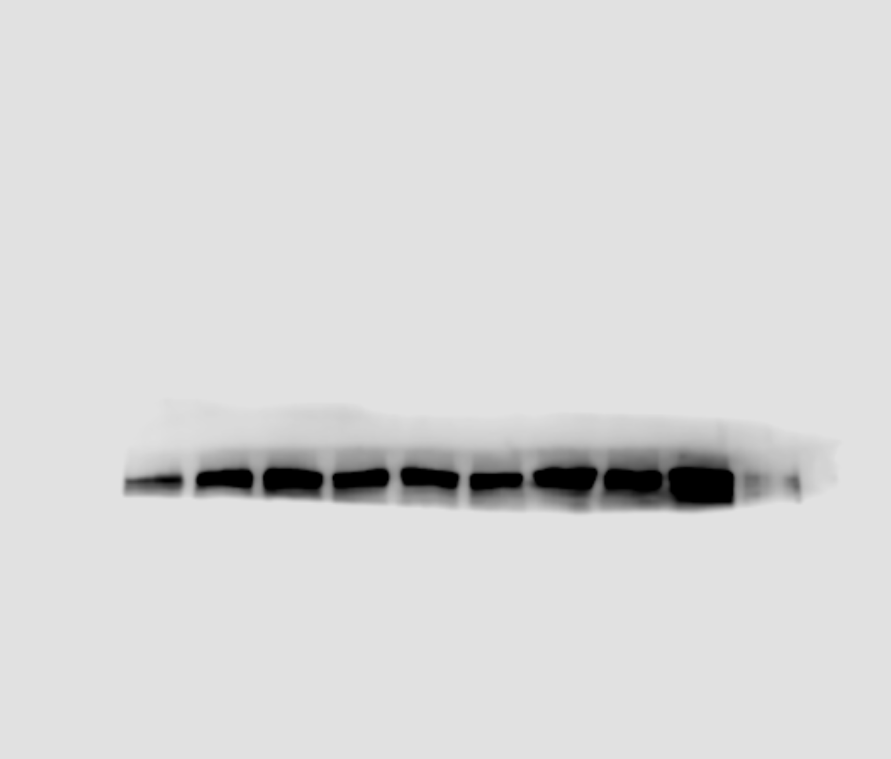


mTOR


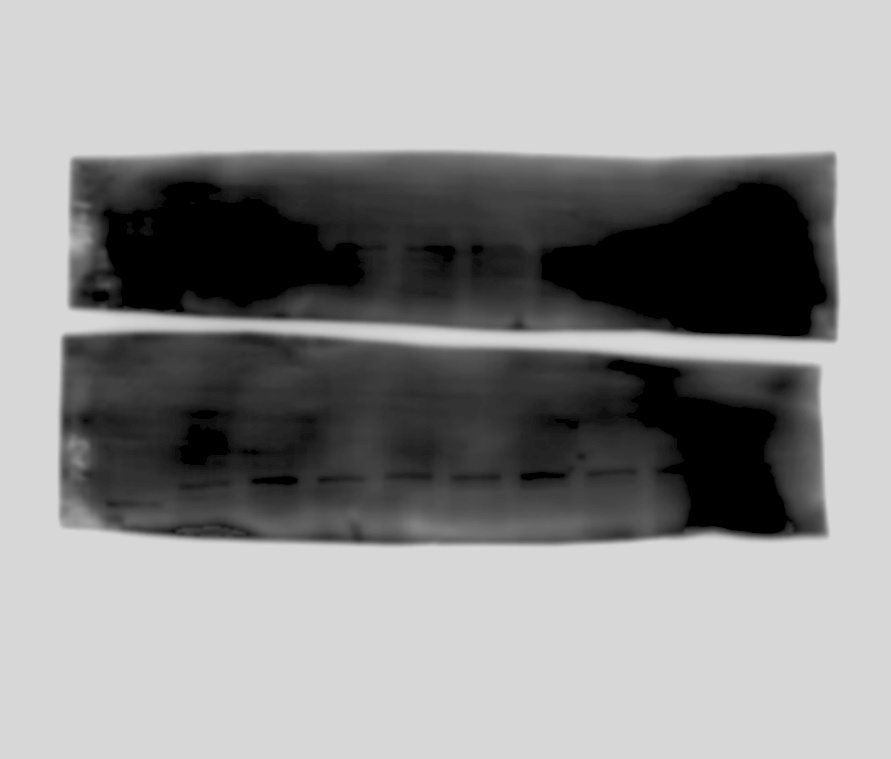


12 kDa

289 kDa

289 kDa

170 kDa

36 kDa

**Fig. S4 a**

GAPDH

P-gp

mTOR

HMGA2

p-mTOR


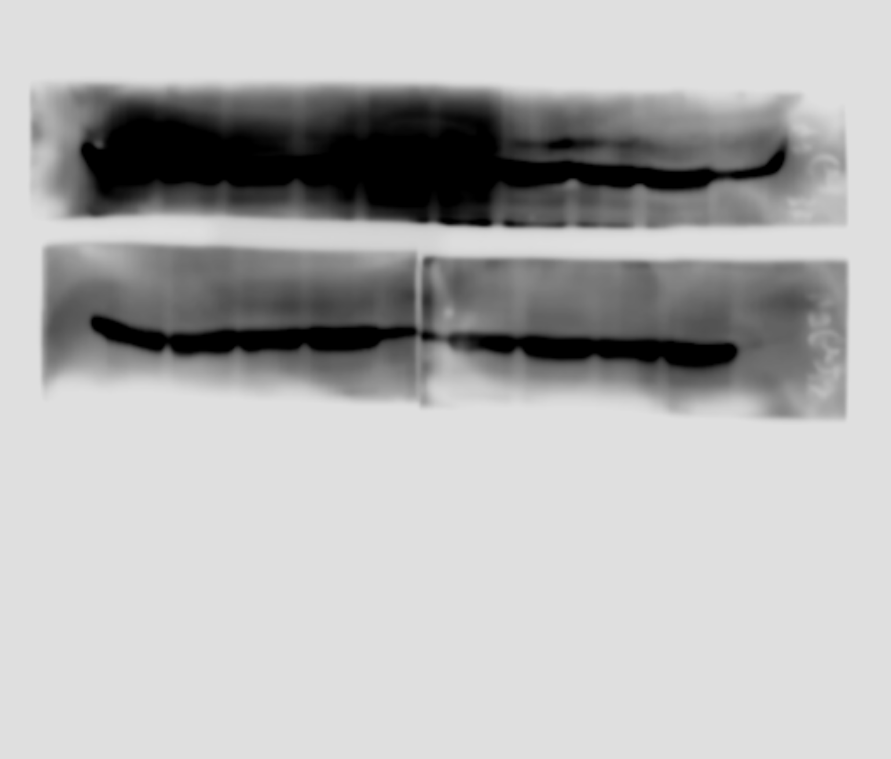


36 kDa


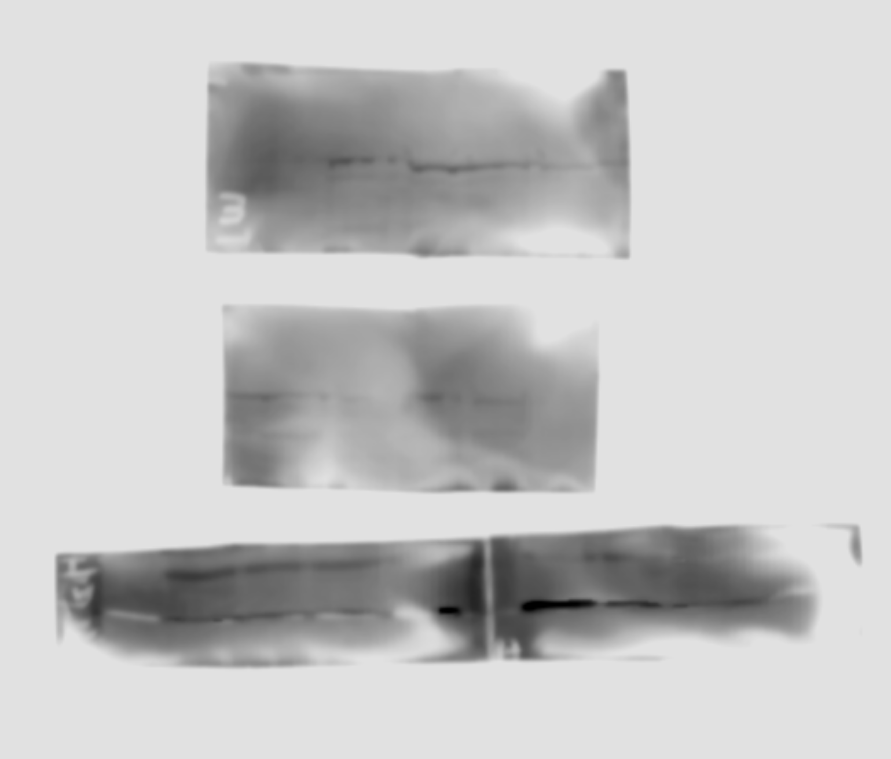


12 kDa


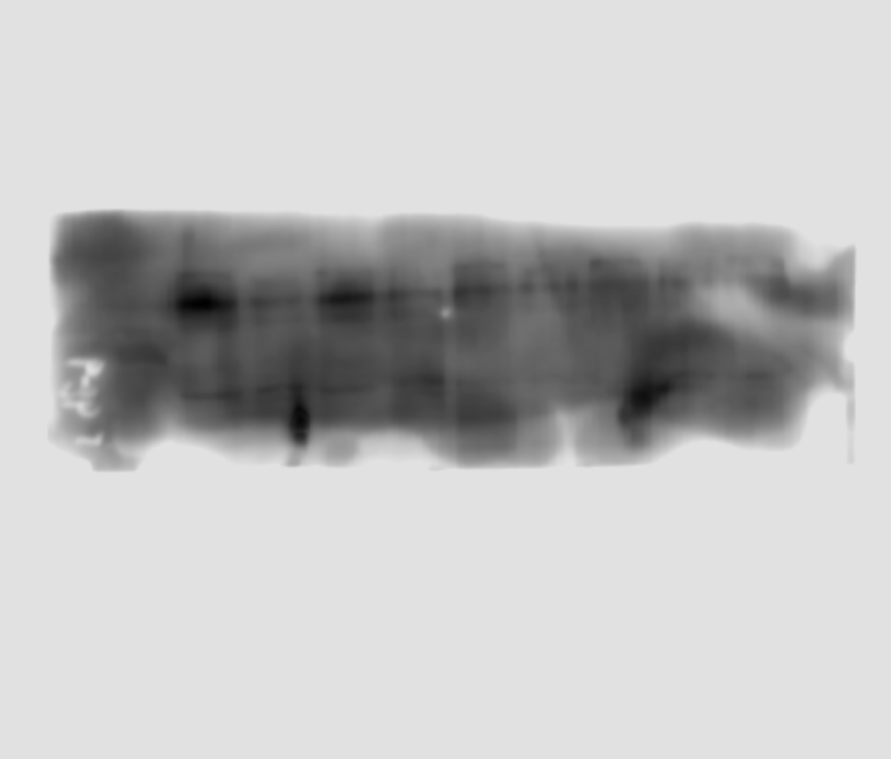


170 kDa


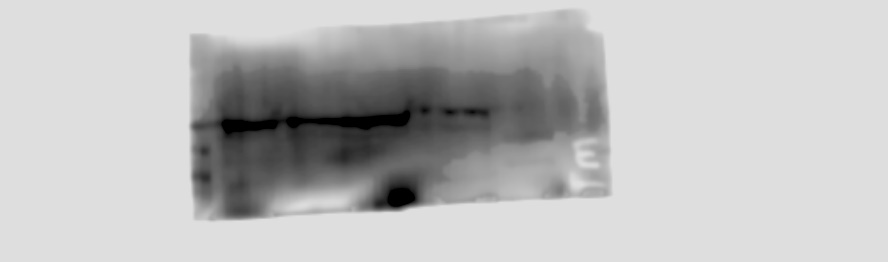


289 kDa


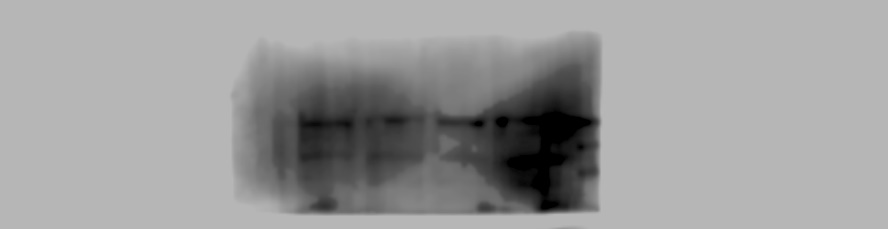


289 kDa

**Fig. S5 a**


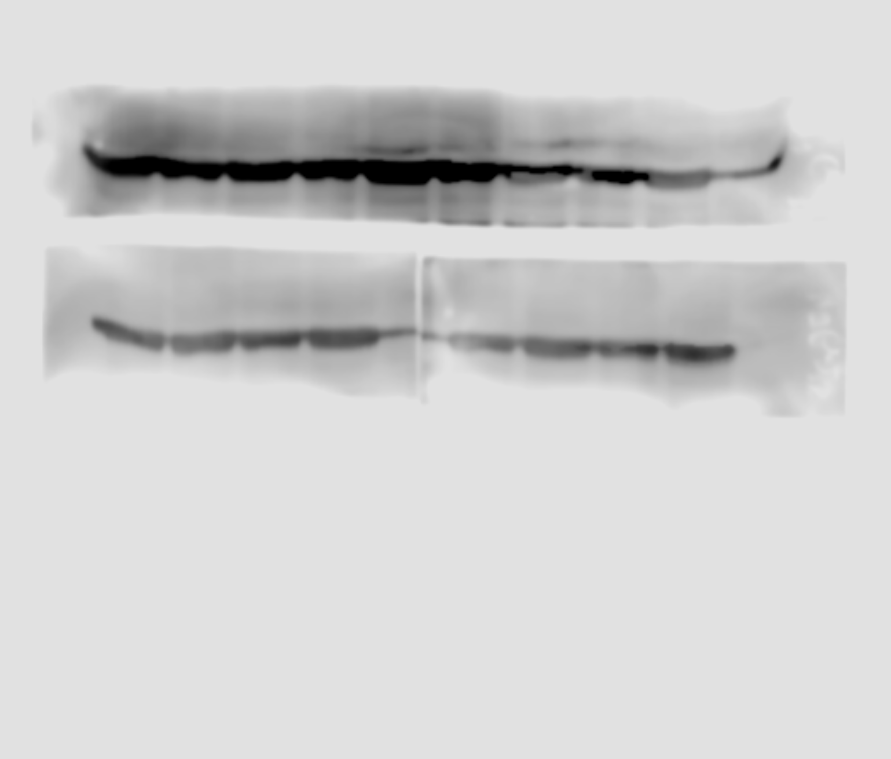


GAPDH

36 kDa

HMGA2


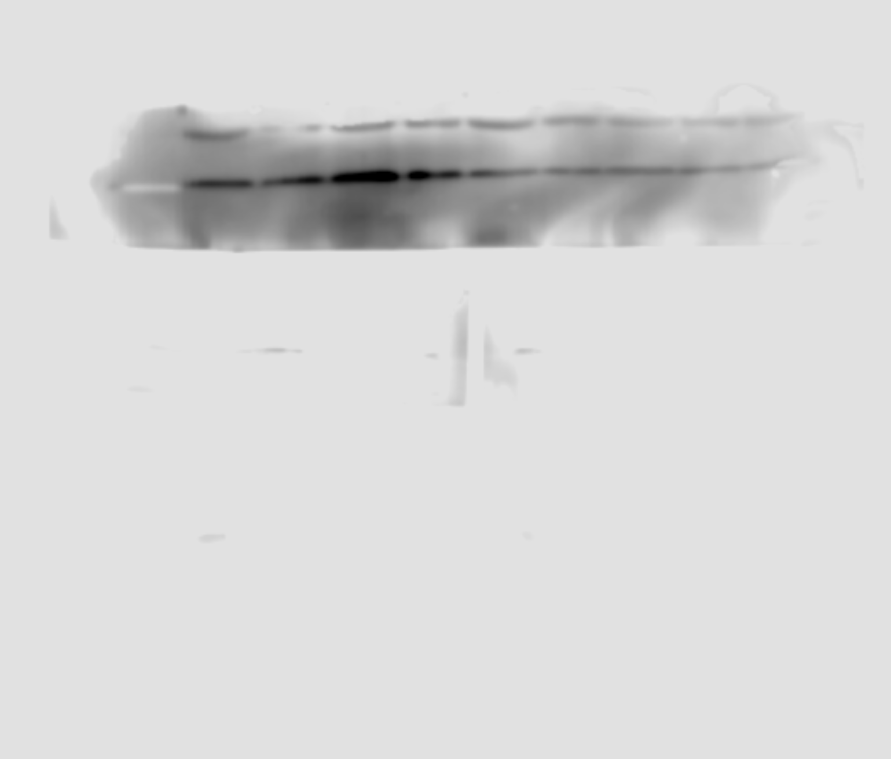


12 kDa

**Fig. S5 b**


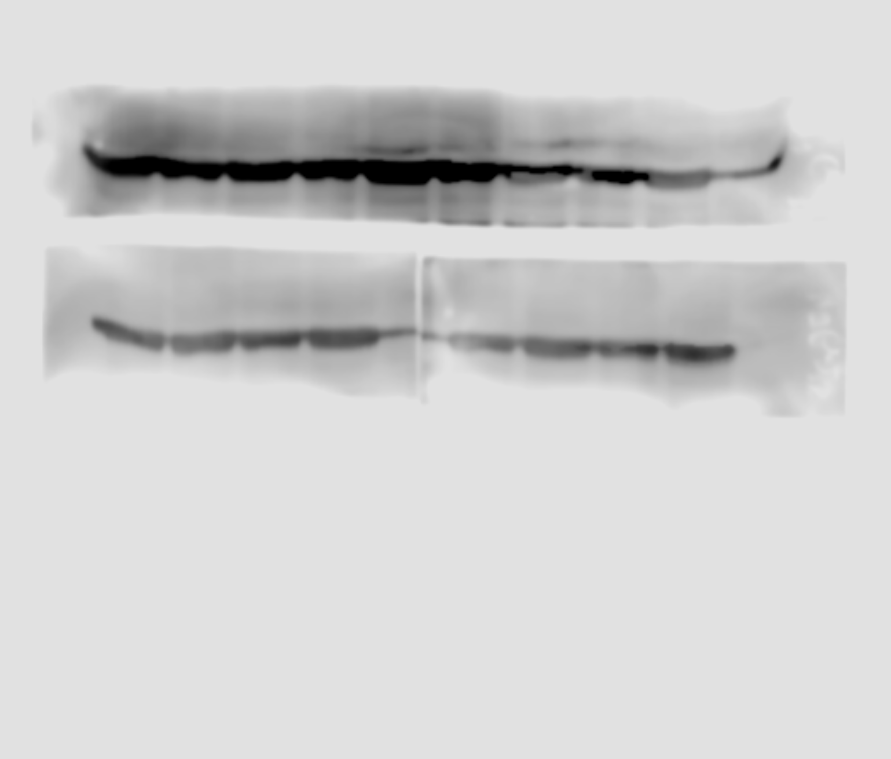


GAPDH

36 kDa


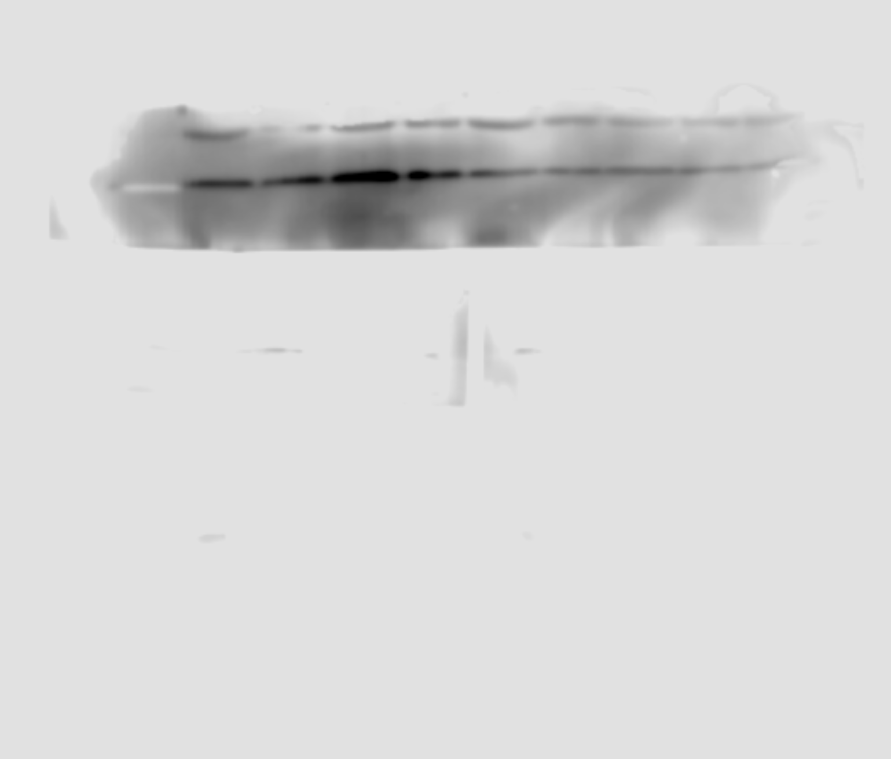


HMGA2

12 kDa
